# A new species of *Epicrionops* (Amphibia: Gymnophiona: Rhinatrematidae) from Ecuador, with integrative morphological and phylogenetic insights into Ecuadorian *Epicrionops* Boulenger, 1883 and *Amazops* Wilkinson, Reynolds & Jacobs, 2021

**DOI:** 10.7717/peerj.21786

**Published:** 2026-11-10

**Authors:** Juan C. Sánchez-Nivicela, Diego F. Cisneros-Heredia, Mario H. Yánez-Muñoz, Claudia Koch, Santiago R. Ron

**Affiliations:** 1 Unidad de Investigación, Instituto Nacional de Biodiversidad (INABIO), Quito, Pichincha, Ecuador; 2 Grupo de Investigación Evolución y Ecología de Fauna Neotropical, Facultad de Ciencias, Universidad Nacional de Colombia, Bogotá, Cundinamarca, Colombia; 3 Museo de Zoología, Laboratorio de Zoología Terrestre, Instituto de Biodiversidad Tropical IBIOTROP, Colegio de Ciencias Biológicas y Ambientales, Universidad San Francisco de Quito, Quito, Pichincha, Ecuador; 4 Department of Geography, King’s College London, University of London, London, United Kingdom; 5 Museum Koenig, Leibniz Institute for the Analysis of Biodiversity Change, Bonn, Germany; 6 Escuela de Biología, Museo de Zoología, Pontificia Universidad Católica del Ecuador, Quito, Pichincha, Ecuador

**Keywords:** Phylogenetic hypothesis, eastern Andean slopes, CT scanning, Cranial osteology, External morphology

## Abstract

The Neotropical tailed caecilians of the genera *Epicrionops* and *Amazops* (Rhinatrematidae) remain poorly known. Here, we examined their morphology, distribution, and molecular phylogenetic relationships using specimens from Ecuador. Our review of museum collections expands the empirical basis for understanding variation in these genera and reveals broader geographic and morphological diversity than previously recognised. Morphological and µCT-based analyses identified consistent diagnostic differences between *Amazops* and *Epicrionops*, particularly in annular counts and cranial bone configurations. We describe a new species of *Epicrionops* from the montane forests of southeastern Ecuador, characterized by a relatively small body size in fully metamorphosed individuals (174–317 mm); a maxillopalatine enclosing the orbit; a long spatulate posterodorsal process of the squamosal; an articular facet of the quadrate contacting the posterior end of the maxillopalatine but not the squamosal; a retroarticular process of the pseudoangular that is dorsally concave and ventrally convex; frontals with slightly notched anterior and posterior margins and curved lateral edges; and an os basale with a narrowing cultriform process and wavy, rounded margins. The pterygoid is large, attached to the maxillopalatine, and barely contacts the quadrate. Phylogenetic analyses of mitochondrial genes (12S, 16S, ND1) recovered the new species in a clade with *E. marmoratus*-*bicolor*, the only congeners for which molecular data are currently available. We report, for the first time, the phylogenetic position of the genus *Amazops* and also expand the known distribution of *Amazops amazops*. Our molecular data suggest that the genus is more diverse than currently recognized, identifying additional potentially undescribed species. These findings provide new integrative evidence on the morphological and phylogenetic diversity of Rhinatrematidae and highlight the need for broader sampling and integrative analyses in the tropical Andes.

## Introduction

The family Rhinatrematidae Nussbaum, 1977 includes 15 species assigned to three genera *Amazops* Wilkinson, Reynolds & Jacobs, 2021, *Epicrionops* Boulenger, 1883 and *Rhinatrema* Duméril & Bibron, 1841, distributed throughout northern South America (Wilkinson & Bittencourt-Silva, 2025; Frost, 2026). Rhinatrematids are diagnosed from other caecilians by lacking fangs and having the orbit entirely enclosed by the maxillopalatine; they are also characterized by open skull roofs, primary jaw adductor muscles extending through the upper temporal fenestra to the mid-dorsum of the cranium, and the absence of an elongate truncus arteriosus (Nussbaum, 1977; Wilkinson, 1996; Wilkinson & Nussbaum, 1996; Wilkinson & Bittencourt-Silva, 2025). Rhinatrematidae is considered the sister group to all other extant caecilians (Wilkinson & Nussbaum, 1996; San Mauro et al., 2014).

*Amazops* is a monotypic genus known from a single locality in montane forest in northeastern Ecuador at 1,000 m above sea level (Wilkinson, Reynolds & Jacobs, 2021). *Epicrionops* includes seven species occurring in lowland and montane forests in Colombia, Ecuador, and Peru, at elevations between approximately 400 and 2,100 m (Maciel et al., 2018; Acosta Galvis, 2023; Frost, 2026). Species of *Epicrionops* and *Amazops* are not easily diagnosed using external morphology because they share similar patterns, annular counts, and body size (Wilkinson, Reynolds & Jacobs, 2021). Additionally, *Epicrionops bicolor* (*E. b. bicolor* and *E. b. subcaudalis*), and *E. petersi* (*E. p. petersi* and *E. p. noblei*) have historically been divided into subspecies (Taylor, 1968).

*Epicrionops* and *Amazops* remain poorly studied genera, largely because the scarcity of specimens in scientific collections has precluded comprehensive taxonomic and systematic reviews (Taylor, 1968; Wilkinson, 1992; Reiss, 1996; Maddin, Russell & Anderson, 2012; Müller, 2020; Wilkinson, Reynolds & Jacobs, 2021). Morphological and molecular studies to date remain insufficient to fully resolve their phylogenetic relationships within and between these genera (Nussbaum, 1977; Wilkinson et al., 2011; Wilkinson, Reynolds & Jacobs, 2021). Recent species descriptions have also not fully clarified synapomorphies within these clades (Wilkinson, 1992; Gower et al., 2010; Wilkinson & Gower, 2010; Wilkinson et al., 2011; Maddin, Russell & Anderson, 2012; Wilkinson, Reynolds & Jacobs, 2021). Despite advances, the taxonomy and evolutionary relationships of *Epicrionops* and *Amazops* remain poorly resolved due to limited specimen availability, morphological similarity among taxa, and incomplete molecular sampling.

Three species of *Epicrionops* have been reported from Ecuador: *Epicrionops bicolor*, *E. marmoratus*, and *E. petersi* (Frost, 2026; Ron, Merino-Viteri & Ortiz, 2024). *Epicrionops bicolor* and *E. marmoratus* occur in the Pacific lowlands and western Andean slopes, whereas *E. petersi* has been reported from Amazonian lowlands and eastern Andean slopes (Taylor, 1968; Taylor & Peters, 1974; Ron, Merino-Viteri & Ortiz, 2024). Field surveys over the last decades have produced additional records of *E. bicolor* and *E. petersi* (Cisneros-Heredia, 2004; Yánez-Muñoz et al., 2007; Yánez-Muñoz et al., 2013; Yánez-Muñoz et al., 2020; Arteaga, Bustamante-Enríquez & Guayasamin, 2013; Yánez-Muñoz, Bejarano-Muñoz & Sánchez-Nivicela, 2019; Camper et al., 2021; Chaves-Portilla, López-Ordóñez & Fernández-Roldán, 2021; Ron, Merino-Viteri & Ortiz, 2024). However, *E. bicolor* has been reported on both sides of the Andes (Taylor, 1968; Yánez-Muñoz et al., 2007; Yánez-Muñoz, Bejarano-Muñoz & Sánchez-Nivicela, 2019), a distribution pattern that is uncommon among amphibians (Caminer & Ron, 2020; Ron, Merino-Viteri & Ortiz, 2024).

A fourth nominal species, *E. lativittatus*, was cited in error by Taylor (1968: Fig. 93–96) as occurring in “eastern Ecuador”. This error was corrected in the errata sheet by Taylor (1968), and the species is therefore known only from its holotype collected in “eastern Perú” (Frost, 2026). The name “*Epicrionops lativittatus*” has also been used in the catalogue of the Muséum d’ Histoire Naturelle de Genève for specimens collected in northwestern Ecuador (MHNG, 2026). However, these records are not supported by published taxonomic studies and originate from the western slopes of Ecuador, where *E. bicolor* and *E. marmoratus* occur and may be confused due to external morphological similarity. We therefore consider these occurrences to most likely represent misidentifications.

Recent field expeditions in the southeastern Andes of Ecuador resulted in the collection of specimens of *Epicrionops* that differ morphologically from all previously described taxa. Using morphological, osteological, and molecular data, we describe these specimens as a new species. We also present an updated comparative assessment of Ecuadorian *Epicrionops* and *Amazops*, provide a preliminary molecular phylogeny for both genera, and discuss the taxonomic implications of our findings.

## Materials & Methods

**Ethics statement.** This study was conducted in accordance with the ethical guidelines for the use of live amphibians and reptiles in field research (Beaupre et al., 2004). Fieldwork was carried out under authorization No 019-2018-IC-FAU-DNB/MAE and within the framework contract for genetic resources access No MAE-DNB-CM-2018-0106, issued by the Ministerio del Ambiente, Agua y Transición Ecológica de Ecuador. ZSFQ specimens are kept under patent number 0018-AD-2016-FAU-DPAP-MA.

**Species concept.** Species were considered as independently evolving metapopulation lineages that can be operationally recognized when isolation from their putative sister lineages can be inferred (De Queiroz, 2007).

**Fieldwork.** Fieldwork was conducted in Río Negro-Sopladora National Park, located along the boundary between the provinces of Morona-Santiago and Azuay in the southeastern Andes of Ecuador. The study area encompassed paramo grasslands, montane, and foothill evergreen forests, spanning an elevational range from 1,000 to 3,400 m in the River Paute basin. Nocturnal surveys were conducted from 19:00 to 23:00. Geographic coordinates and elevations were recorded using a Garmin handheld GPS device (WGS84 datum). Natural history data were extracted from field notes. Individuals were photographed alive and subsequently maintained in ventilated containers with controlled humidity for up to 12 h after collection. Specimens were euthanized using a 2% lidocaine spray solution. Muscle tissue samples were preserved in 95% ethanol, and whole specimens were fixed in 10% formalin and later stored in 70% ethanol. Ecosystem classification followed the Ministerio de Ambiente del Ecuador (MAE) (2013) proposal.

**Taxonomic sampling.** In addition to the five specimens collected during fieldwork, we examined 50 rhinatrematid specimens deposited in the following museums: The Natural History Museum, London (BMNH); Instituto Nacional de Biodiversidad del Ecuador, Quito (DHMECN); Museo de Ciencias Naturales “Gustavo Orcés”, Escuela Politécnica Nacional del Ecuador, Quito (MEPN); Museo de Zoología, Universidad Técnica Particular de Loja, Loja (MUTPL); Museo de Zoología, Universidad del Azuay, Cuenca (MZUA); Museo de Zoología, Universidad Tecnológica Indoamérica, Quito (MZUTI); Museo de Zoología, Pontificia Universidad Católica del Ecuador, Quito (QCAZ); and, Museo de Zoología, Universidad San Francisco de Quito, Quito (ZSFQ). Examined specimens are listed in Appendix I. This sampling, to our knowledge, represents the most comprehensive set of *Epicrionops* and *Amazops* specimens currently available in Ecuadorian collections.

Information on an additional 36 specimens from different localities used for comparative diagnoses was obtained from the literature (Taylor, 1968; Wilkinson, Reynolds & Jacobs, 2021; Chaves-Portilla, López-Ordóñez & Fernández-Roldán, 2021; Fernández-Roldán, Lynch & Medina-Rangel, 2022). We also compared specimens from Colombia, Ecuador and Peru using photographs available in BioWeb Ecuador (https://bioweb.bio/faunaweb/amphibiaweb), Batrachia (http://www.batrachia.com) and iNaturalist (https://www.inaturalist.org).

**Identification of comparative material.** Assignments of examined specimens to species of *Epicrionops* and *Amazops* were based on concordance of external morphology, meristic data, osteological features observed in µCT scans, and geographic provenance with information from original descriptions and subsequent taxonomic literature (especially Taylor, 1968; Wilkinson, 1992; Wilkinson et al., 2011; Wilkinson, Reynolds & Jacobs, 2021). We did not examine name-bearing type specimens of all previously described species and therefore acknowledge that some identifications should be regarded as provisional. In cases of taxonomic ambiguity, particularly within the *E. bicolor–marmoratus* complex and populations historically assigned to *E. petersi*, identifications were treated conservatively and interpreted within a comparative framework rather than as definitive species assignments. This approach minimizes circularity in species diagnosis and avoids reliance on potentially unstable historical identifications. This uncertainty reflects the well-known taxonomic complexity of rhinatrematid caecilians and the historical limitations of available comparative material. Importantly, the recognition and diagnosis of the new species described herein do not rely on any single comparative taxon, but on a unique combination of external, osteological, and molecular characters that consistently differentiate it from all congeners examined.

The names of new species reported herein constitute a published work according to the International Commission on Zoological Nomenclature (ICZN) and are effectively published under that Code from the electronic edition alone; the nomenclatural acts it contains have been recorded in ZooBank, the online registration system for the ICZN. The ZooBank LSIDs (Life Science Identifiers) can be found by appending the LSID to the prefix http://zoobank.org/.

**Morphology and colouration.** (Fig. 1) Morphological definitions and terminology follow Taylor (1968) and Lynch (1999), as refined by Wilkinson, Reynolds & Jacobs (2021). Identification of larval and developmental stages using external characteristics was based on Dünker, Wake & Olson (2000), Kupfer, Nabhitabhata & Himstedt (2005a), Kupfer, Nabhitabhata & Himstedt (2005b) and Müller (2020). Sex and maturity were determined by observation of protruded sexual organs (phallus) or by abdominal incision to inspect the gonads. Measurements were taken with a digital calliper (0.05 mm accuracy, rounded to the nearest 0.1 mm) under a stereomicroscope, reported as a range, mean, and standard deviation. Measurements and counts of external morphological traits (Table 1) follow Wei et al. (2025). Scale morphology was examined by extracting scales from the anterior, middle, and posterior regions of the body of specimen ZSFQ 4138. The largest of three scales extracted from each of these regions was measured and compared with the images and measurements in Taylor (1972). The individual measurements are presented in Appendix II.

**Figure 1:**
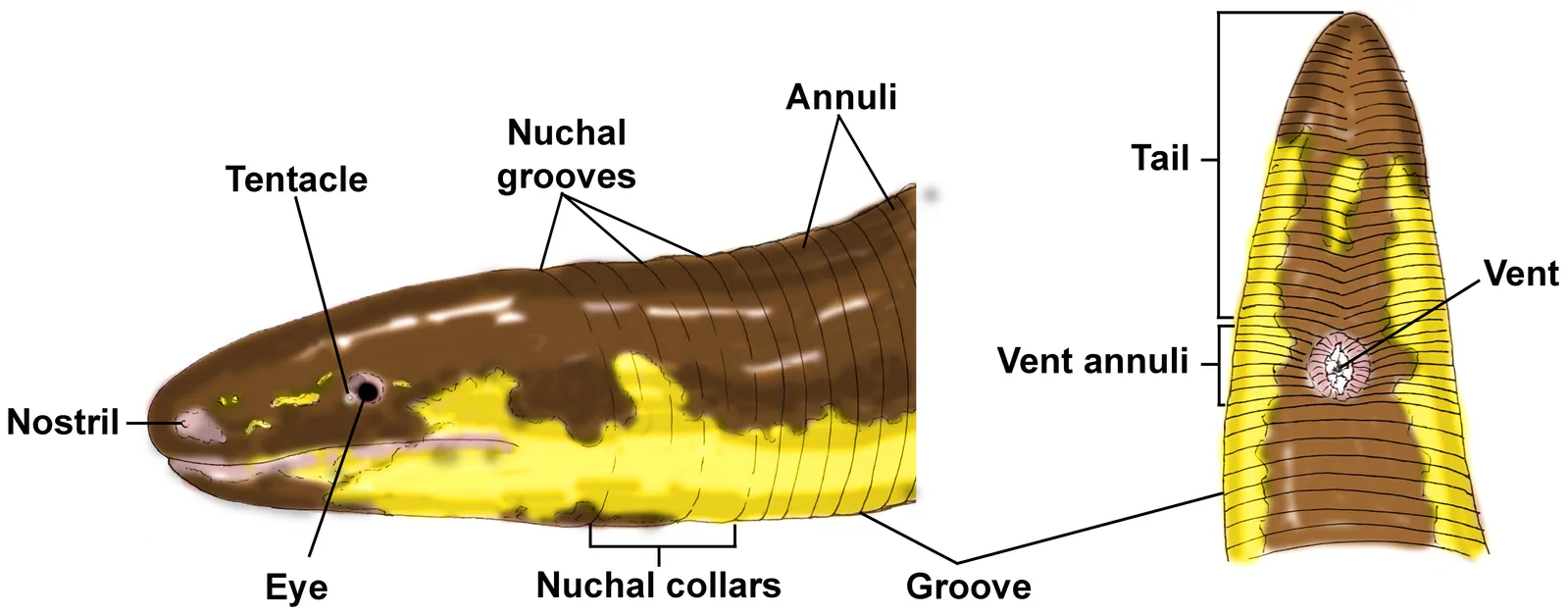
Morphological characters of external anatomy of the genera *Epicrionops* and *Amazops*. Illustrations reference ZSFQ 4136 (*Epicrionops*), by Juan C. Sánchez-Nivicela.

**Table 1:** Measurements or counts used for the recognition of morphological traits of Rhinatrematidae. Codes, names and definitions follow Wei et al. (2025).

**Code**	**Name**	**Description of measurement or count**	**Code**	**Name**	**Description of measurement or count**
**TOL**	Total length	Distance from tip of snout to tail tip.	**NS**	Nostril-snout distance	Distance between nostril and tip of snout.
**SVL**	Snout-vent length	Distance from tip of snout to posterior margin of vent.	**NJ**	Nostril-jaw distance	Distance between naris and jaw angle.
**TRL**	Trunk length	Distance from first collar groove to posterior end of vent.	**ND**	Naris diameter	Diameter of naris.
**TL**	Tail length	Distance from posterior edge of vent to tail tip.	**NLP**	Naris to margin of lip distance	Distance between naris and margin of lip.
**BW1**	Body width 1	Body width measured at first nuchal groove.	**NFNG**	Naris to nuchal groove distance	Distance between naris and first nuchal groove.
**BW2**	Body width 2	Body width measured in middle of body.	**SJ**	Snout-jaw distance	Distance between tip of snout and jaw angle.
**BW3**	Body width 3	Body width measured at anterior edge of vent disc.	**LN**	Lower jaw to nuchal groove	Distance between tip of lower jaw and first nuchal groove.
**BW4**	Body width 4	Body width measured at second nuchal groove.	**LJL**	Lower jaw length	Distance from tip of lower jaw to jaw angle.
**BH**	Body height	Body height measured at midbody.	**JNG**	Jaw to nuchal groove distance	Distance between jaw angle and first nuchal groove.
**HL**	Head length	Distance from tip of snout to posterior edge of skull.	**TAW**	Tail width at posterior vent	Tail width measured at posterior vent.
**HW1**	Head width 1	Head width measured at jaw angle.	**VA**	Vent annuli	Annuli interrupted by vent.
**HW2**	Head width 2	Head width measured at lateral edge of first nuchal groove.	**ADD**	Anal disc diameter	Diameter of anal disc.
**HH1**	Head height 1	Head height measured at lateral edge of first nuchal groove.	**C1**	1st collar length	Length of first collar measured laterally.
**HH2**	Head height 2	Head height measured at jaw angle.	**C2**	2nd collar length	Length of second collar measured laterally.
**STFG**	Snout to 1st collar groove	Distance from tip of snout to 1st collar groove.	**CM**	Circumference at midbody	Circumference at midbody.
**STSG**	Snout to 2nd collar groove	Distance from tip of snout to 2nd collar groove.	**TA1**	Total annuli 1	Total number of annuli in dorsal count.
**STTG**	Snout to 3rd collar groove	Distance from tip of snout to 3rd collar groove.	**TA2**	Total annuli 2	Total number of annuli in ventral count.
**IOD**	Interorbital distance	Distance between medial margins of each eye.	**PVA**	Post-vent annuli	Total number of annuli counted from posterior edge of the vent.
**ED**	Eye diameter	Horizontal distance across eye.	**PMT**	Premaxillary- maxillary teeth	Total number or formula of remaxillary-maxillary teeth.
**EN**	Eye-nostril distance	Distance between eye and nostril.	**PPT**	Prevomers-palatines teeth	Total number or formula of prevomers-palatines teeth.
**EJ**	Eye-jaw distance	Distance between eye and jaw angle.	**DT**	Dentary teeth	Total number or formula of dentary teeth.
**SE**	Snout-eye distance	Distance from tip of snout to eye.	**ST**	Splenial teeth	Total number or formula of splenial teeth.
**TLP**	Tentacle-lip distance	Distance between tentacle and margin of lip.	**VN**	Vertebrae number	Number of vertebrae.
**IND**	Internostril distance	Distance between medial margins of nares.			

Colour patterns observed in live and preserved specimens were classified into the following categories: (a) uniform, (b) striped, (c) mottled, (d) speckled, and (e) splashed with middorsal line (Fig. 2). Life colour descriptions were based on photographs taken in the field by Juan C. Sánchez-Nivicela (JSN), Santiago R. Ron (SRR), Mario H. Yánez-Muñoz (MYM), José M. Falcón (JMF), Juan D. Fernández-Roldán (ICN 58514), and literature references (Yánez-Muñoz et al., 2007; Chaves-Portilla, López-Ordóñez & Fernández-Roldán, 2021; Wilkinson, Reynolds & Jacobs, 2021; Fernández-Roldán, Lynch & Medina-Rangel, 2022). CT-scan images were reconstructed and edited by Claudia Koch (CK) and JSN.

**Figure 2:**
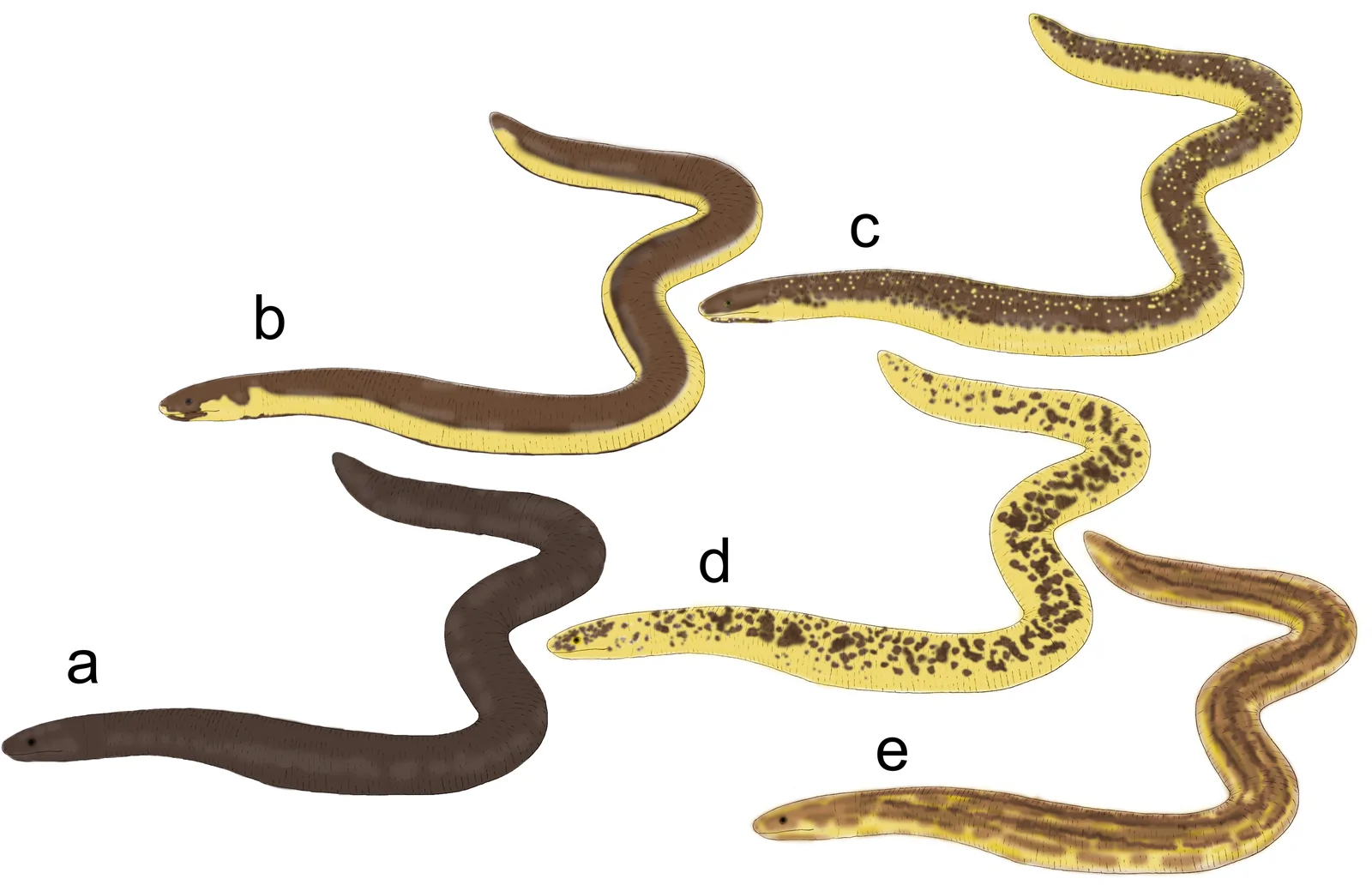
Colour patterns in the *Epicrionops* and *Amazops* species. (A) Uniform (USNM 159794, *E. petersi*), (B) striped (ZSFQ 4136, *E. jameskenti* sp. nov.), (C) mottled (QCAZ-A 11861, *E. bicolor-marmoratus* complex), (D) speckled (BMNH 1956.1.15.87, *E. marmoratus*), (E) splashed with middorsal line (BMNH 1946.9.5.61, *E. parkeri*). Illustrations by Juan C. Sánchez-Nivicela.

**Digital osteology.** Data on skulls and mandibles were obtained from 10 specimens (seven specimens of the genus *Epicrionops* and three *Amazops*) with the aid of high-resolution micro-computed tomography (µCT) scanning procedures using a desktop device (Bruker SkyScan 1173, Kontich, Belgium) at the Leibniz Institute for the Analysis of Biodiversity Change/Museum Koenig (LIB, Bonn, Germany). The scans were conducted using an X-ray beam with a 43 kV source voltage and 116 µA current, without a filter. Rotation steps of 0.25–0.3° were used with a frame averaging of 3–5, recorded over a 180° (DHMECN 04600, 09311, 10828, 15867, QCAZ-A 74837, 01979, ZSFQ 01354, 04136) rotation or 360° (ZSFQ 02817, 02818), resulting in 800 (DHMECN 04600, 09311, 10828, 15867, ZSFQ 01354, 04136), 960 (QCAZ-A 01979, 74837) or 1200 (ZSFQ 02817, 02818) projections of 500 ms exposure time each. The magnification setup generated data with an isotropic voxel size of 8.2–19.9 µm. We used CT-Vox for Windows, 64-bit version 2.6 (Bruker, µCT), to visualise the skulls. Regular radiographs were obtained with a digital X-ray system (Inspex 20i). Osteological observations derived from µCT scans were used primarily for qualitative comparisons and proportional analyses, and not to infer character polarity or homology beyond what is supported by previous anatomical studies of rhinatrematid caecilians. Recognition of bones, terminology, and morphology follows Nussbaum (1977) and Wilkinson, Reynolds & Jacobs (2021). Tooth counts (left–right) follow Wei et al. (2025) (Table 1, Fig. 3). Although numerous osteological characters were described, only a subset showed consistent differences between genera and species. Other observed variations should be interpreted cautiously, as they may reflect ontogenetic or intraspecific variation.

**Figure 3:**
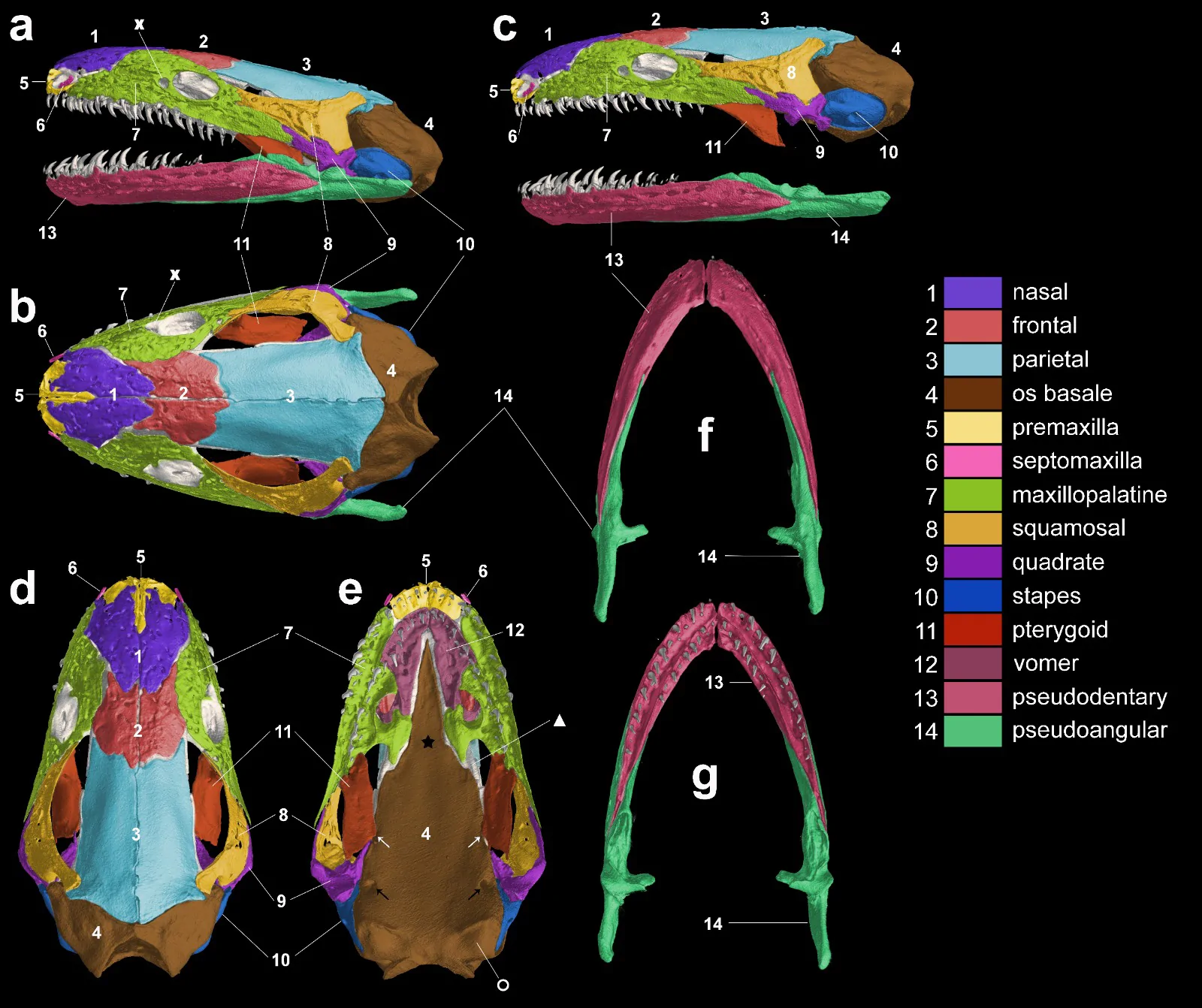
Skull and mandible bones of the holotype ZSFQ 4136 of *Epicrionops jameskenti* sp. nov. (A) Lateral and (B) dorsal views of the articulated skull and mandible, (C) lateral view of the skull and mandible dislocated, (D) skull dorsal view, (E) skull ventral view, (F) mandible ventral view, (G) mandible dorsal view. White triangle = sphenethmoid, white X = foramen for tentacular ducts, black star = cultriform process, white arrow = basipterygoid process, black arrow = carotid foramen, white ring = otic capsule.

**Phylogenetic analyses.** To estimate the phylogenetic relationships of *Epicrionops* and *Amazops*, we sequenced three mitochondrial genes: 12S rRNA (12S; complete or nearly complete sequence), 16S rRNA (16S; partial sequence), NADH dehydrogenase subunit 1 (ND1; complete sequence), and their flanking tRNAs. We extracted genomic DNA from liver samples preserved in 96% ethanol from six individuals across six localities in Ecuador (Table 2; data in Appendix I). DNA was extracted using standard guanidine thiocyanate extraction protocols. We used a polymerase chain reaction (PCR) to amplify DNA fragments. Primers for 12S were obtained from Goebel, Donnelly & Atz (1999) and Pauly, Hillis & Cannatella (2004), for 16S from Heinicke, Duellman & Hedges (2007), and for ND1 from San Mauro et al. (2004) and Wiens et al. (2005). PCR amplification was performed following standard protocols, and sequencing was performed by the Macrogen Sequencing Team (Macrogen Inc., Seoul, Korea).

**Table 2:** Genbank accession numbers for newly generated DNA sequences used in the phylogenetic analyses. Specimen ZSFQ 4136 also has a tissue number QCAZ-A 77398. Collection locality is provided in Appendix I.

Species	Voucher	12S	16S	ND1
*Amazops amazops*	QCAZ-A 42405	(PQ461843)	(PQ461838)	(PQ462301)
*A. amazops*	QCAZ-A 56870	(PQ461844)	(PQ461839)	(PQ462300)
*A. amazops*	QCAZ-A 70228	(PQ461845)	(PQ461840)	(PQ462298)
*A.* aff. *amazops*	QCAZ-A 74837	(PQ461846)	(PQ461841)	(PQ462299)
*Epicrionops bicolor- marmotratus* complex	QCAZ-A 11861	(PQ461847)	(PQ461842)	–
*E. jameskenti* sp. nov	ZSFQ 4136	(PQ461848)	(PQ461837)	(PQ462302)

The new sequences were assembled in Geneious 9.0 (Kearse et al., 2012) and then exported to Mesquite 3.81 (Maddison & Maddison, 2023). GenBank accession numbers for newly generated sequences are available in Table 2. Our matrix also included Rhinatrematidae sequences of the 12S, 16S, ND1, and Cytochrome oxidase I (COI) genes available in GenBank (*Rhinatrema* used as the outgroup). GenBank sequences were published by Feller & Hedges (1998), San Mauro et al. (2004), Frost et al. (2006), Zhang & Wake (2009), Maciel et al. (2018), and Acosta-Galvis, Torres & Pulido-Santacruz (2019). Each DNA fragment was aligned using MAFFT 7.511 (Katoh & Standley, 2013).

Phylogenetic relationships were inferred using a concatenated dataset under a maximum likelihood (ML) framework as an optimality criterion. The final DNA matrix had 4995 bp (available at http://zenodo.org under DOI: 10.5281/zenodo.15962824). The degree of missing data was 0.322; 1100 sites were parsimony informative, 543 were singleton sites, and 3352 were invariant sites. We partitioned the matrix by gene and codon position (nine partitions in total), and each partition was analysed under the GTR + R + I model of evolution. Phylogenetic search was conducted with the multicore version of IQ-TREE 2.2.0 (Nguyen et al., 2015). To assess branch support, we performed 1000 ultrafast bootstrap searches (-bb 1000) and 1000 replicates for the SH-like approximate likelihood ratio test (-alrt 1000) (Guindon et al., 2010). We considered branches with bootstrap values > 95 and SH-aLRT values > 80 strongly supported. To calculate uncorrected p-distances of 16S, we used the package Ape 5.0 (Paradis & Schliep, 2019) in the R version 4.5.2 (R Core Team, 2025) and only compared fragments overlapping by more than 500 bp. Our phylogenetic results should be interpreted as hypothesis-generating rather than definitive, given the limited taxon sampling and reliance on mitochondrial markers only.

## Results

### Phylogenetic analyses

Our phylogenetic tree (Fig. 4) recovered two basally diverging clades within *Epicrionops*. The first is strongly supported and corresponds to species assignable to *Amazops.* The other has weak support and is composed of *E. parkeri* and a strongly supported clade composed of *E. bicolor, E. marmoratus* and the new species, *E. jameskenti* sp. nov.

**Figure 4:**
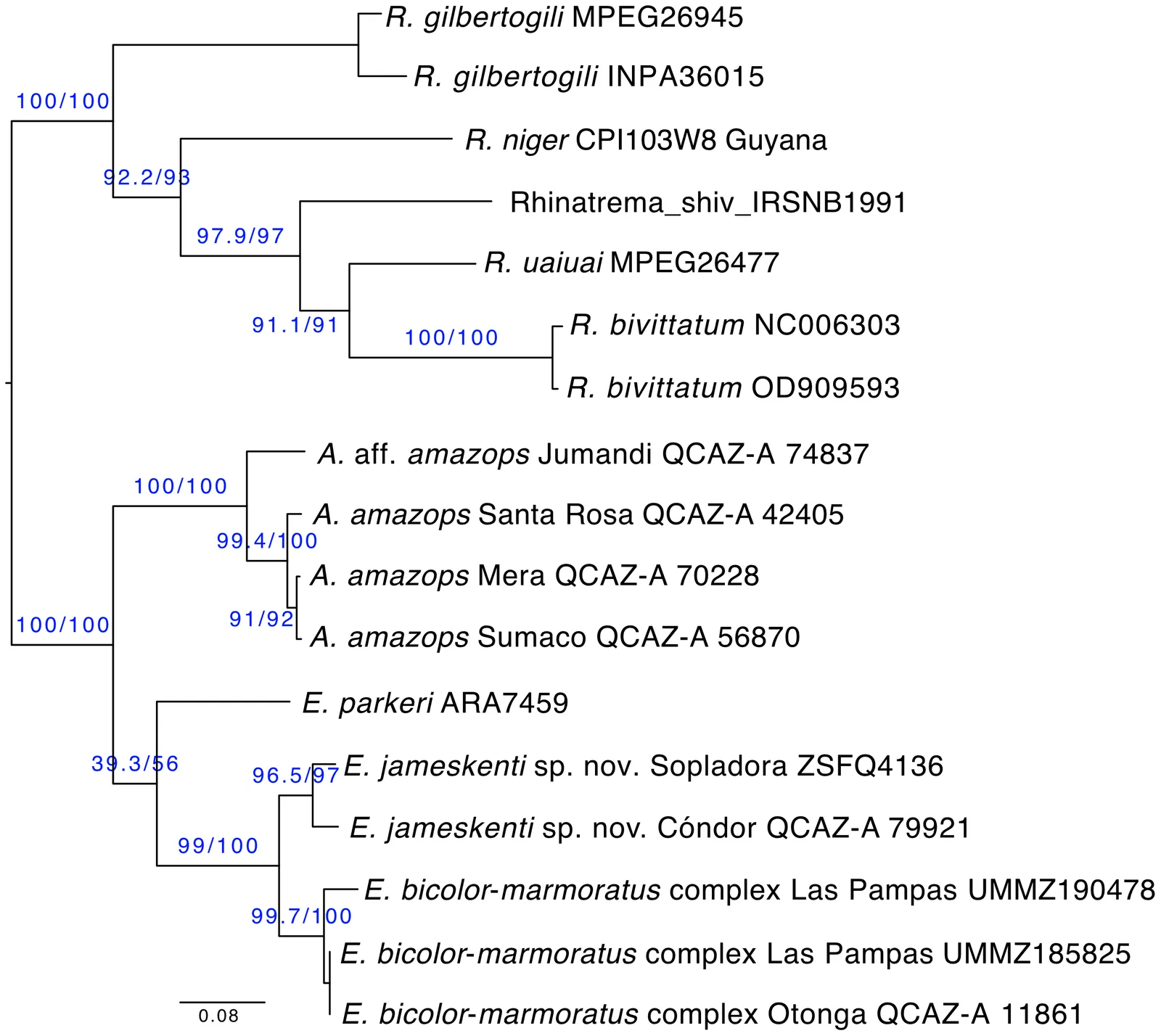
Phylogenetic relationships of Rhinatrematidae. Maximum likelihood tree obtained for genes 16S-rRNA, 12S-rRNA, ND1, and CO1 from a matrix of 4995 bp for 17 specimens. Branch support (in blue) are aLRT values before the fraction line and bootstrap after the fraction line. For each specimen, the voucher number is shown, or in its absence, the GenBank accession number. Tree rooted using *Rhinatrema* as outgroup.

The specimen of *Amazops* from Jumandi (QCAZ-A 74837) showed a genetic distance of 5.6% to 6.6% (16S rRNA) from *Amazops amazops* and may represent an undescribed species. However, additional sampling is required to assess intraspecific variation in both morphological and molecular characters. The specimens from Río Negro-Sopladora (ZSFQ 4136) and Cordillera del Cóndor (QCAZ-A 79921) formed a clade sister to specimens assigned to the *E. bicolor*-*marmoratus* complex (see species-level analyses below). Uncorrected *p*-genetic distances for 16S genes among specimens of the *E. bicolor-marmoratus* complex ranged from 2.36 to 3.03%. Genetic distances between *E. jameskenti* sp. nov. and those of the *E. bicolor-marmoratus* complex ranged 5.02–6.53%. Genetic distance between both populations of the new species was 1.95%.

### Genus-level analyses

**Comparative external morphology.** All adult and fully metamorphosed specimens examined (*Amazops*: ♀ = 3, ♂ = 6; *Epicrionops*: ♀ = 11, ♂ = 15, sex unknown = 2) measured less than 389 mm in **TOL** and had cylindrical bodies (**TOL** 14–27 times **BW2**) with orthoplicate grooves on the body and tail, consistent with the conditions described by Nussbaum (1977). *Amazops* and *Epicrionops* were similar in general appearance, but *Amazops* showed shorter total length and fewer venter and post-vent annuli (**TOL** 184–295 mm, **VA** 3–5, **PVA** 10–14) compared with *Epicrionops* (**TOL** 214–389 mm, 4–8 **VA**, 14–21 **PVA**; Table 3). These differences were consistent across the samples examined and supported the morphological distinction between the two genera.

**Table 3:** External morphology characters from species of the genera *Amazops* and *Epicrionops*. Value before slash (/) is the count or measurement of the holotypes. Values inside parentheses (in bold): $\bar {x}$ = mean, ±  = standard deviation.

	***Amazops***	***E. bicolor-marmoratus*** ** complex**	***E. petersi***	***E. jameskenti*** ** sp. nov**
** **	***n* = 9**	***n* = 22**	***n* = 2**	***n* = 5**
Total length	173/184–295	*E. bicolor*: 234, *E. marmoratus*: 316/214–389	283/300	211/174–317
	**(x¯229.9, ±34.5)**	**(x¯286.4, ±43.9)**		**(x¯225.4, ±53.8)**
Snout-vent length	167–287	180–335	264	157–287
	**(x¯215.7, ±35.3)**	**(x¯243.4, ±43.6)**		**(x¯205.0, ±49.2)**
Trunk length	159–264	177–320	258	140–276
	**(x¯201.7, ±32.3)**	**(x¯238.2, ±42.3)**		**(x¯187.8, ±51.7)**
Tail length	7.6/5.6–10.8	*E. bicolor*: 8.3, *E. marmoratus*: 18.9/3.3–17.4	12.3/14.5	10.8/9.1–16.5
	**(x¯8.2, ±1.6)**	**(x¯12.4, ±4.1)**		**(x¯12.2, ±2.8)**
Body width 1	5.8–11.2	7.5–15.2	11.2	6.7–10.6
	**(x¯7.9, ±1.7)**	**(x¯10.2, ±1.8)**		**(x¯8.4, ±1.4)**
Body width 2	8.5–12.7	9.8–18.6	14.9	7.6–21.9
	**(x¯9.9, ±1.4)**	**(x¯14.7, ±2.3)**		**(x¯12.1, ±5.6)**
Body width 3	4.9–8.1	7.1–13.5	10.5	5.8–12.0
	**(x¯6.2, ±1.1)**	**(x¯10.6, ±1.9)**		**(x¯8.3, ±2.3)**
Body width 4	6–11.3	7.5–15.5	11.4	6.9–11.9
	**(x¯8.2, ±1.7)**	**(x¯10.8, ±1.9)**		**(x¯9.0, ±1.8)**
Body height	3.9–11.2	6.4–18.8	11.7	6.7–14.8
	**(x¯7.2, ±2.3)**	**(x¯11.8, ±3.0)**		**(x¯9.1, ±3.3)**
Head length	8–14.1	10.1–19.2	12.2	10.1–13.2
	**(x¯10.9, ±2)**	**(x¯13.4, ±2.0)**		**(x¯11.0, ±1.2)**
Head width 1	5.8–9.9	7.0–14.6	10.8	6.1–10.2
	**(x¯7.5, ±1.3)**	**(x¯9.7, ±1.8)**		**(x¯7.8, ±1.5)**
Head width 2	6.1–11.1	8.0–15.4	11.2	6.6–10.4
	**(x¯7.9, ±1.6)**	**(x¯10.2, ±1.8)**		**(x¯8.4, ±1.4)**
Head height 1	3.7–7.8	5.5–13.3	8.3	4.8–8.4
	**(x¯5.4, ±1.3)**	**(x¯7.5, ±1.9)**		**(x¯6.1, ±1.4)**
Head height 2	3.5–7.1	5.0–11.9	7.5	4.6–6.6
	**(x¯5, ±1.2)**	**(x¯6.8, ±1.7)**		**(x¯5.6, ±0.8)**
Snout to 1st collar groove	9.5–14.1	10.2–20.8	12.7	10–13.5
	**(x¯11.3, ±1.7)**	**(x¯13.7, ±2.4)**		**(x¯11.2, ±1.4)**
Snout to 2nd collar groove	10.9–17.4	12.2–23.0	14.7	11.4–15.6
	**(x¯13.2, ±2.3)**	**(x¯16.0, ±2.6)**		**(x¯12.9, ±1.7)**
Snout to 3rd collar groove	14.7–23.9	15.8–31.6	20.2	14.8–21.5
	**(x¯18.1, ±3.4)**	**(x¯21.7, ±3.6)**		**(x¯17.3, ±2.5)**
Interorbital distance	3.6–5.7	4.2–7.6	5.3	4.0–5.3
	**(x¯4.5, ±0.7)**	**(x¯5.2, ±0.9)**		**(x¯4.6, ±0.4)**
Eye diameter	0.4–0.7	0.4–0.7	0.5	0.5–0.6
	**(x¯0.5, ±0.1)**	**(x¯0.6, ±0.1)**		**(x¯0.6, ±0)**
Eye-nostril distance	3.2–4.5	3.6–6.1	4.1	3.2–4.6
	**(x¯3.7, ±0.4)**	**(x¯4.6, ±0.6)**		**(x¯3.8, ±0.6)**
Eye-jaw distance	3.7–6.1	3.1–9.3	6.9	3.8–6.2
	**(x¯5, ±0.9)**	**(x¯6.2, ±1.7)**		**(x¯4.6, ±0.9)**
Snout-eye distance	4.1–5.2	3.7–7.8	5.3	3.7–5.5
	**(x¯4.6, ±0.4)**	**(x¯5.5, ±0.9)**		**(x¯4.7, ±0.7)**
Tentacle-lip distance	0.6–1.1	0.9–2.2	1.6	0.7–1.5
	**(x¯0.9, ±0.1)**	**(x¯1.3, ±0.3)**		**(x¯1.2, ±0.3)**
Internostril distance	1.4–2.3	1.2–2.5	1.5	1.4–2.0
	**(x¯1.7, ±0.3)**	**(x¯1.6, ±0.4)**		**(x¯1.7, ±0.2)**
Nostril-snout distance	0.7–1	0.5–1.6	0.7	0.6–1.4
	**(x¯0.8, ±0.1)**	**(x¯0.9, ±0.3)**		**(x¯0.8, ±0.3)**
Nostril-jaw distance	6.6–10.9	7.6–14.9	10.8	7.3–11.8
	**(x¯8.9, ±1.5)**	**(x¯11.0, ±2.1)**		**(x¯8.9, ±1.8)**
Naris diameter	0.1–0.4	0.1–0.4	0.3	0.1–0.3
	**(x¯0.2, ±0.1)**	**(x¯0.2, ±0.1)**		**(x¯0.2, ±0.1)**
Naris to margin of lip distance	0.5–1.1	0.5–1.4	1.1	0.6–0.9
	**(x¯0.8, ±0.2)**	**(x¯0.9, ±0.3)**		**(x¯0.7, ±0.1)**
Naris to nuchal groove distance	8.3–31.5	9.9–19.1	11.9	9–12.3
	**(x¯12.6, ±7.2)**	**(x¯13.4, ±2.5)**		**(x¯10.4, ±1.2)**
Snout-jaw distance	7.7–12	8.2–17.0	12.3	7.5–12.5
	**(x¯9.9, ±1.4)**	**(x¯12.1, ±2.3)**		**(x¯9.4, ±1.9)**
Lower jaw to nuchal groove	9.6–15.8	10.2–19.9	14.9	9.1–13.3
	**(x¯11.5, ±2)**	**(x¯13.9, ±2.4)**		**(x¯11.6, ±1.7)**
Lower jaw length	8.2–11.8	7.4–15.6	12.0	7.5–11.6
	**(x¯9.5, ±1.2)**	**(x¯11.4, ±2.1)**		**(x¯9.2, ±1.6)**
Jaw to nuchal groove distance	1.5–4.7	1.8–11.1	3.4	1.3–3.8
	**(x¯2.5, ±1.2)**	**(x¯3.1, ±2.0)**		**(x¯2.5, ±0.9)**
Tail width at posterior vent	4.2–7.1	6.3–12.5	9.6	4.9–10.1
	**(x¯5.2, ±1)**	**(x¯9.2, ±1.7)**		**(x¯6.8, ±1.9)**
Vent annuli	3–5	4.0–8.0	5	5–7
	**(x¯4.1, ±0.6)**	**(x¯5.8, ±0.9)**		**(x¯6, ±1)**
Anal disc diameter	1.4–3.7	2.3–5.2	4.2	1.6–4.2
	**(x¯2.4, ±0.8)**	**(x¯3.7, ±0.8)**		**(x¯2.7, ±1.1)**
1st collar length	1.5–2.5	1.5–4.0	2.4	1.4–2.2
	**(x¯1.8, ±0.3)**	**(x¯2.5, ±0.6)**		**(x¯1.8, ±0.3)**
2nd collar length	3.6–7.5	4.2–8.4	7.6	4–6.7
	**(x¯5.5, ±1.2)**	**(x¯6.0, ±1.0)**		**(x¯5.1, ±1.1)**
Circumference at midbody	23–44	31.0–68.0	45	25–65
	**(x¯31.6, ±6.3)**	**(x¯46.8, ±8.6)**		**(x¯37, ±16)**
Total annuli 1	<275/261–350	*E. bicolor*: 260, *E. marmoratus*: 341/254–335	282/282	291/258–291
	**(x¯292.9, ±27.6)**	**(x¯295.7, ±22.4)**		**(x¯274.6, ±15.5)**
Total annuli 2	249–347	239–326	270	257–290
	**(x¯286.6, ±28.8)**	**(x¯282.9, ±23.1)**		**(x¯269.8, ±13.3)**
Post-vent annuli	10/10–14	*E. bicolor*: 11, *E. marmoratus*: 23/14–21	16/16	19/16–20
	**(x¯11.7, ±1.3)**	**(x¯17.9, ±2.0)**		**(x¯17.8, ±1.6)**

Proportional morphometric analyses revealed clear differences between *Amazops* and Ecuadorian species of *Epicrionops* (Table 4). *Amazops* exhibited a relatively slender body (**BW2/TOL** = 3.7–4.9; $\bar {x}$ = 4.3, SD = 0.4) and shorter tail (**TL/TOL** = 2.4–4.6; $\bar {x}$ = 3.6, SD= 0.7) compared to *Epicrionops*, in which are these radios were consistently higher (**BW2/TOL** = 4.3–6.9; $\bar {x}$ = 5.1, SD = 0.6, **TL/TOL** = 1.4–6.1; $\bar {x}$ = 4.3, SD = 1.6). Head proportions also differed notably: *Amazops* showed a narrower head (**HW1/HL** = 63.1–82.4%; $\bar {x}$ = 69.7, SD = 5.8) than *Epicrionops* species (**HW1/HL** = 59.0–88.4%; $\bar {x}$ = 72.0, SD = 6.2). Tail width at the posterior vent to body width (**TAW/BW3**) remained relatively constant across species ($\bar {x}$ = 0.8–0.9). Head height and width relationships (**HW1/HH2, HW1/HW2, HH2/HH1**) were also relatively conserved across taxa. Finally, the ratios between body width and interorbital distance (**HW1/IND**), and interorbital and internostril distances (**IOD/IND**) were markedly greater in *Epicrionops* (**HW1/IND** = 4.3–8.5%; $\bar {x}$ = 5.8, SD = 1.1, **IOD/IND** = 2.5–4.7%; $\bar {x}$ = 3.2, SD = 0.5) than in *Amazops* species (**HW1/IND** = 4.0–5.8%; $\bar {x}$ = 4.7, SD = 0.7, **IOD/IND** = 2.3–3.3%; $\bar {x}$ = 2.8, SD = 0.4), evidencing a distinct cranial distinction between *Amazops* and *Epicrionops*. Despite some overlapping among variables, the combined morphometric patterns consistently differentiated *Amazops* from *Epicrionops*.

**Table 4:** Relative sizes rank (% to percentages and ∝ to ratios) from species of the genera *Amazops* and *Epicrionops*. Values inside: x¯ = mean, ± = standard deviation.

	*Amazops*	*E. bicolor-marmoratus* complex	*E. jameskenti* sp.nov	*E. petersi*
**BW2/TOL (%)**	3.7–4.9	4.3–6.3	4.4–6.9	5.0
	**x¯**=4.3, ± = 0.4	**x¯**=5.1, ± = 0.6	**x¯**=5.2, ± = 1.0	
**TL/TOL (%)**	2.4–4.6	1.4–6.0	5.1–6.1	4.8
	**x¯**=3.6, ± = 0.7	**x¯**=4.4, ± = 1.5	**x¯**=5.4, ± = 0.4	
**HW1/HL (%)**	63.1–82.4	59.0–86.0	60.9–77.5	88.4
	**x¯**=69.7, ± = 5.8	**x¯**=71.6, ± = 5.3	**x¯**=70.6, ± = 6.1	
**EN/HL (%)**	28.8–45.9	29.3–37.8	31.8–42.0	33.4
	**x¯**=34.9, ± = 5.4	**x¯**=34.2, ± = 2.0	**x¯**=34.9, ± = 4.2	
**BW2/BH (** **∝)**	1.0–2.3	0.9–1.4	0.8–1.7	1.2
	**x¯**=1.5, ± = 0.4	**x¯**=1.1, ± = 0.1	**x¯**=1.1, ± = 0.3	
**BW3/BH (** **∝)**	0.5–1.4	0.7–1.5	0.8–1.1	0.9
	**x¯**=0.9, ± = 0.3	**x¯**=0.9, ± = 0.2	**x¯**=0.9, ± = 0.1	
**TAW/BW3 (** **∝)**	0.8–1.0	0.8–1.0	0.7–0.8	0.9
	**x¯**=0.8, ± = 0.1	**x¯**=0.9, ± = 0.1	**x¯**=0.8, ± = 0.0	
**HW1/HH2 (** **∝)**	1.3–1.9	1.1–2.0	1.3–1.5	1.4
	**x¯**=1.5, ± = 0.2	**x¯**=1.5, ± = 0.2	**x¯**=1.4, ± = 0.1	
**HW1/HW2 (** **∝)**	0.9–1.0	0.8–1.2	0.8–1.0	1.0
	**x¯**=0.9, ±=0	**x¯**=0.9, ± = 0.1	**x¯**=0.9, ± = 0.1	
**HH2/HH1 (** **∝)**	0.8–1.1	0.8–1.0	0.8–1.0	0.9
	**x¯**=0.9, ± = 0.1	**x¯**=0.9, ± = 0.1	**x¯**=0.9, ± = 0.1	
**HW1/IND (** **∝)**	4.0–5.8	4.4–8.5	4.3–5.0	7.1
	**x¯**=4.7, ± = 0.7	**x¯**=6.0, ± = 1.0	**x¯**=4.6, ± = 0.3	
**IOD/IND (** **∝)**	2.3–3.3	2.5–4.7	2.6–2.9	3.4
	**x¯**=2.8, ± = 0.4	**x¯**=3.3, ± = 0.5	**x¯**=2.8, ± = 0.1	

Larvae of *Amazops* (Stage 35 = 1, Stage 36 = 1, Stage 37 = 2) and *Epicrionops* (Stage 34 = 1, Stage 35 = 2, Stage 36 = 1, Stage 37 = 3, Stage 38 = 2) were similar in general appearance, but at Stage 37, *Amazops* retained the distal portion of the tail fin, whereas it was almost completely resorbed in *Epicrionops*.

**Comparative cranial osteology (Figs. 5–7, Table 5).** Cranial osteological variation was examined in 10 specimens (*Amazops* = 3; *Epicrionops* = 7), following the comparative framework described by Nussbaum (1977) and Wilkinson, Reynolds & Jacobs (2021). The characters described here summarized discrete patterns observed within a limited sample and were intended as a heuristic grouping of continuous variation. In several cases, multiple aspects of shape, proportion, and articulation covary and were therefore treated as composite characters (*sensu* Wilkinson, 1995). Potential influences of ontogeny, size, bilateral symmetry, and intraspecific variation were considered.

**Figure 5:**
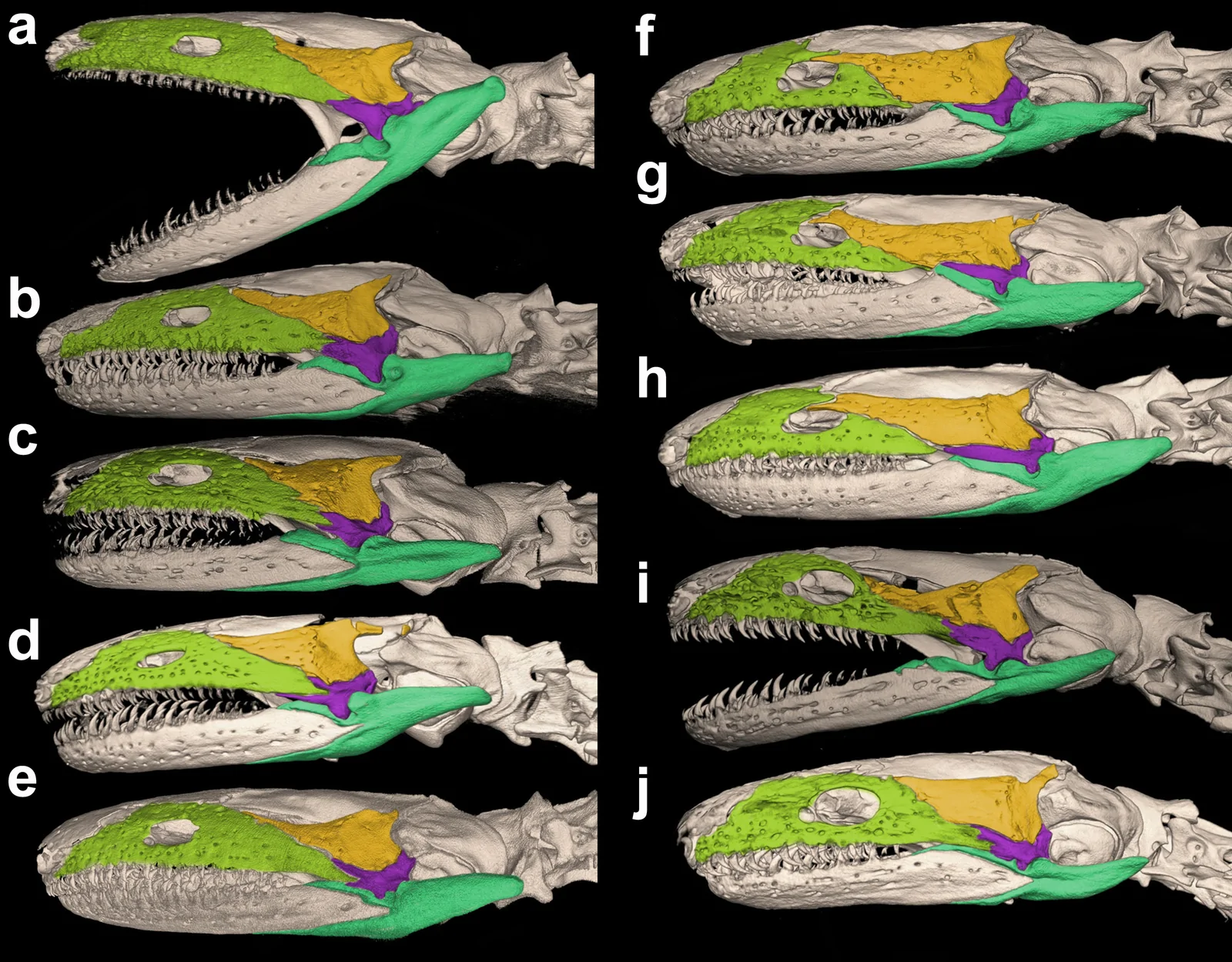
CT-scan images of skulls and mandibles in lateral view of *Epicrionops* (A–E, I, J) and *Amazops* (F–H). (A) *E. bicolor-marmoratus* complex “Santo Domingo” (ZSFQ 2817), (B) *E. bicolor-marmoratus* complex “Santo Domingo” (ZSFQ 2818), (C) *E. bicolor-marmoratus* complex “Cotopaxi” (QCAZ-A 1979), (D) *E. bicolor-marmoratus* complex “Bolivar” (DHMECN 9311), (E) *E. bicolor-marmoratus* complex “El Oro” (DHMECN 10828), (F) *Amazops* sp. “Napo” (QCAZ-A 74837), (G) *Amazops amazops*. “Tena” (DHMECN 15867), (H) *Amazops amazops* “Tena” (ZSFQ 1354), (I) *E. jameskenti* sp. nov. “Morona Santiago” (ZSFQ 4136), (J) *E. jameskenti* sp. nov. “Zamora Chinchipe” (DHMECN 4600). Colour legend: green = maxillopalatine (1), yellow = squamosal (2), purple = quadrate (3), greenish blue = pseudoangular (4), for further descriptions see Fig. 3. Note: specimen DHMECN 9311 has a fractured skull.

**Figure 6:**
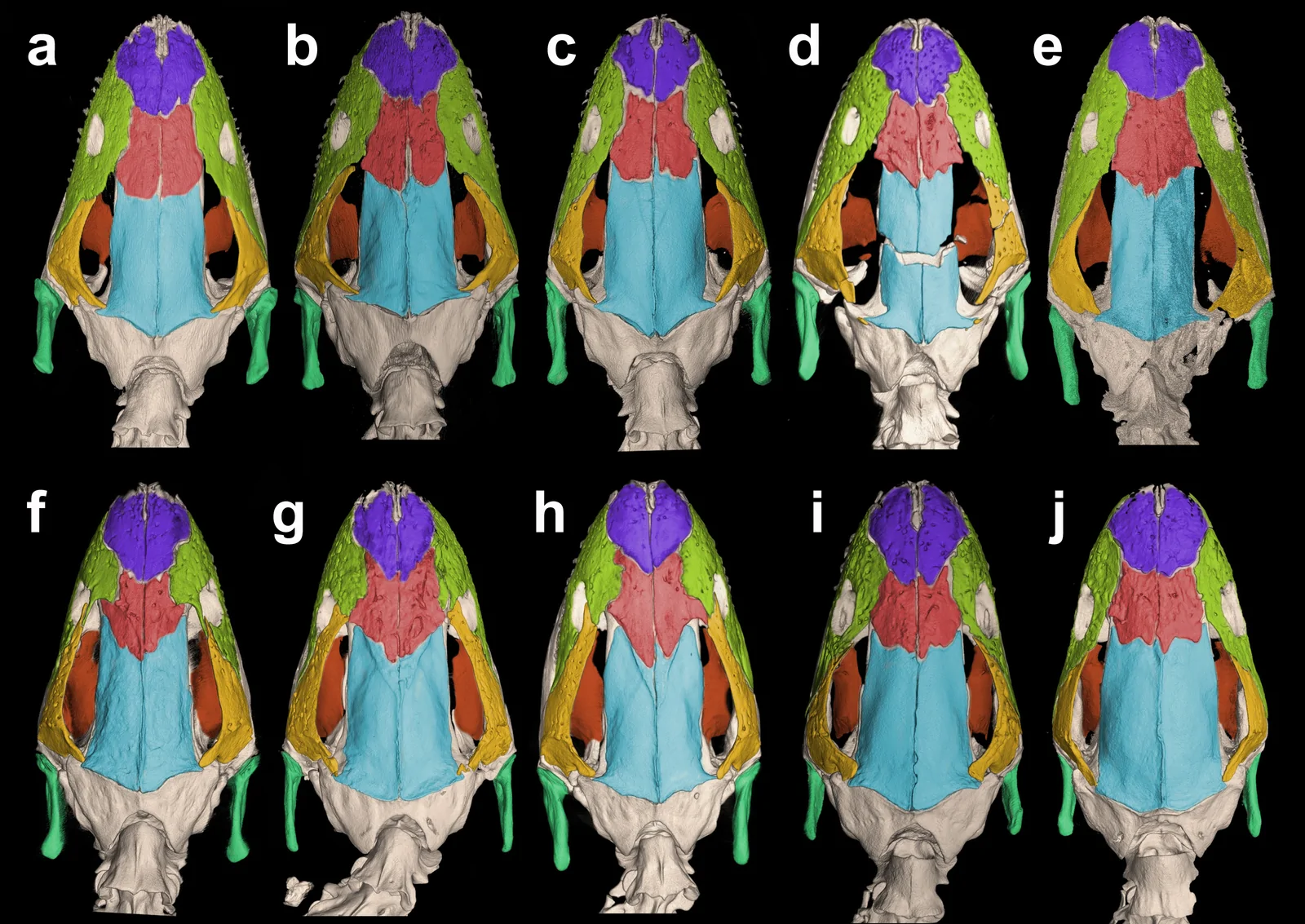
CT-scan images of skulls and mandibles in dorsal view of *Epicrionops* (A–E, I, J) and *Amazops* (F–H). (A) *E. bicolor-marmoratus* complex “Santo Domingo” (ZSFQ 2817), (B) *E. bicolor-marmoratus* complex “Santo Domingo” (ZSFQ 2818), (C) *E. bicolor -marmoratus* complex “Bolivar” (QCAZ-A 1979), (D) *E. bicolor-marmoratus* complex “Bolivar” (DHMECN 9311), (E) *E. bicolor-marmoratus* complex “El Oro” (DHMECN 10828), (F) *Amazops* sp. “Napo” (QCAZ-A 74837), (G) *Amazops amazops* “Tena” (DHMECN 15867), (H) *Amazops amazops* “Tena” (ZSFQ 1354), (I) *E. jameskenti* sp. nov. “Morona Santiago” (ZSFQ 4136), (J) *E. jameskenti* sp. nov. “Zamora Chinchipe” (DHMECN 4600). Colour legends: purple blue = nasal (1), green = maxillopalatine (2), red = frontal (3), yellow = squamosal (4), sky blue = parietal (5), greenish blue = pseudoangular (6), for further descriptions see Fig. 3. Note: specimen DHMECN 9311 has a fractured skull.

**Figure 7:**
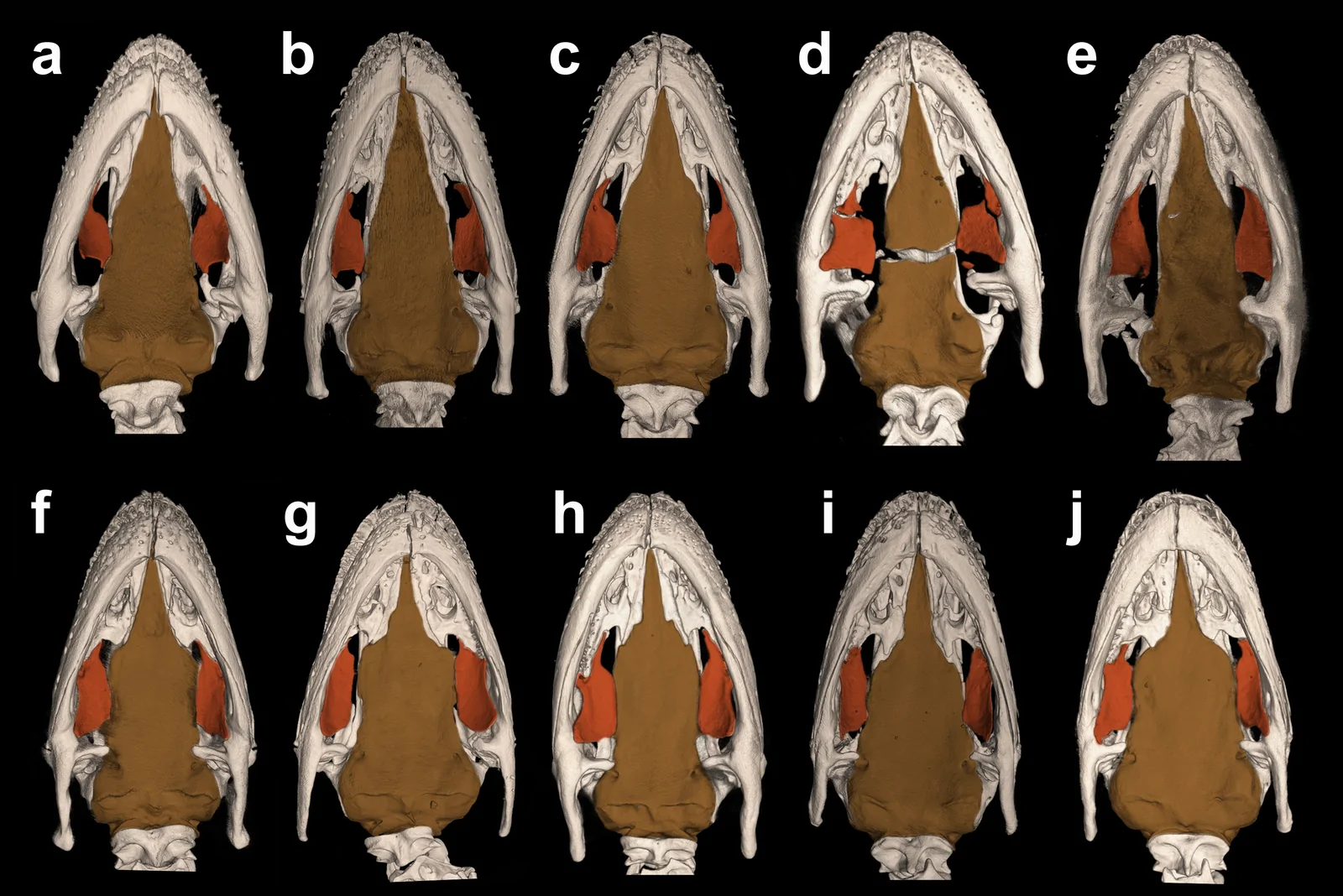
CT-scan images of skulls and mandibles in ventral view of *Epicrionops* (A–E, I, J) and *Amazops* (F–H). (A) *E.bicolor-marmoratus* complex “Santo Domingo” (ZSFQ 2817), (B) *E. bicolor-marmoratus* complex “Santo Domingo” (ZSFQ 2818), (C) *E. bicolor -marmoratus* complex “Bolivar” (QCAZ-A 1979), (D) *E. bicolor-marmoratus* complex “Bolivar” (DHMECN 9311), (E) *E. bicolor-marmoratus* complex “El Oro” (DHMECN 10828), (F) *Amazops* sp. “Napo” (QCAZ-A 74837), (G) *Amazops amazops* “Tena” (DHMECN 15867), (H) *Amazops amazops* “Tena” (ZSFQ 1354), (I) *E. jameskenti* sp. nov. “Morona Santiago” (ZSFQ 4136), (J) *E. jameskenti* sp. nov. “Zamora Chinchipe” (DHMECN 4600). Colour legends: dark orange = pterygoid (1), brown = os basale (2), for further descriptions see Fig. 3. Note: specimen DHMECN 9311 has a fractured skull.

All specimens shared a zygokrotaphic skull, premaxillae and nasals as separate bones, squamosals not in contact with, and distant from, the frontals; parasphenoid portion of os basale prolonged and cultriform anteriorly. Two general cranial conditions were recognised: (i) the orbit entirely enclosed by the maxillopalatine, with the quadrate articulating with the posterior end of the maxillopalatine (Figs. 5A–5E, 5I–5J); and (ii) the orbit enclosed jointly by the maxillopalatine and squamosal, with the quadrate not contacting the maxillopalatine (Figs. 5F–5H).

**Table 5:** Skull characters from species of the genera *Epicrionops* and *Amazops*. Museum numbers and localities provinces: DHMECN-15867: Pastaza, QCAZ-74837: Napo, QCAZ-01979: Cotopaxi, ZSFQ-02818-7: Santo Domingo de los Tsáchilas, DHMECN-09311: Bolívar, DHMECN-10828: El Oro, DHMECN-04600: Zamora Chinchipe, ZSFQ-04138: Morona Santiago.

**Museum number**	**Species**	**Sex**	**Development st.**	**Maxillopalatine (orbital enclosure)**	**Squamosal (postero-dorsal process shape)**	**Maxillopalatine– squamosal suture (type)**	**Quadrate– maxillopalatine relationship (shape)**	**Quadrate articular facet (type)**	**Frontal (shape)**	**Os basale (shape)**	**Pterygoid (shape)**
DHMECN-15867	*Amazops amazops*	Male	Fully metamorphosed	Partial	C	2	E	3	C	C	C
QCAZ-74837	*Amazops* aff. *amazops*	Female	Adult	Partial	C	2	E	3	C	C	C
QCAZ-01979	*Epicrionops bicolor- marmoratus* complex	Male	Adult	Complete	A	1	B	1	A	A	A
ZSFQ-02818	*E. bicolor- marmoratus* complex	Male	Adult	Complete	A	1	A	1	A	A	A
ZSFQ-02817	*E. bicolor- marmoratus* complex	Female	Adult	Complete	A	1	A	1	A	A	A
DHMECN-09311	*Epicrionops bicolor- marmoratus* complex	Female	Adult	Complete	A	1	B	2	B	B	B
DHMECN-10828	*Epicrionops bicolor- marmoratus* complex	Male	Adult	Complete	B	1	D	3	B	B	B
DHMECN-04600	*Epicrionops jameskenti* sp. n.	Male	Fully metamorphosed	Complete	D	3	D	1	D	D	D
ZSFQ-04136	*Epicrionops jameskenti* sp. n.	Male	Fully metamorphosed	Complete	D	3	D	2	D	D	D

The squamosals exhibited consistently curved dorsal margins and concave posterior margins, but differed in the relative length, curvature, and distal expansion of the postero-dorsal process, allowing recognition of four shapes: shape A, the postero-dorsal process is long, slender, and evenly curved, tapering gradually to a pointed distal tip (Figs. 5A–5D); shape B, the process is distinctly shorter, strongly curved, and blunt distally (Fig. 5E); shape C, the process is short, weakly curved, and triangular or spear-like in outline, lacking distal elongation (Figs. 5F–5H); shape D, the process is long and robust, with a spatulate distal expansion rather than a pointed tip (Figs. 5I–5J). The distinction between shapes A and C was based primarily on proportional length relative to squamosal height, degree of curvature, and distal morphology: shape A was elongate and tapering, whereas shape C was short, minimally curved, and triangular.

The configuration of the suture between maxillopalatine and squamosal varied in extent and form and was described as three types: type 1, an extensive, slightly curved contact along most of the anteroventral margin (Figs. 5A–5E); type 2, two short and discrete points of contact (Figs. 5F–5H); and type 3, a moderately extensive, angular contact restricted to the middle of the anteroventral margin (Figs. 5I–5J).

The quadrate articulated dorsally with the squamosal in all specimens, but varied in its relationship with the posterior end of the maxillopalatine. Five shapes summarised this variation: shape A, the quadrate was in inferior contact with the posterior end of the maxillopalatine, which was embedded between the squamosal and quadrate (Figs. 5A–5B); shape B, the quadrate anchored in a serrated manner to the posterior end of the maxillopalatine, with the upper tip embedded between squamosal and quadrate (Fig. 5C); shape C, the quadrate covered both edges of the posterior end of the maxillopalatine, with no contact between that end and the squamosal (Fig. 5D); shape D, the quadrate contacted the posterior end of the maxillopalatine dorsally, without contact between that end and the squamosal (Figs. 5E, 5I–5J); shape E, the quadrate did not contact the maxillopalatine; within this condition, variation in quadrate length (short to elongate; Figs. 5F–5H) was interpreted as continuous and potentially influenced by size or ontogeny rather than treated as discrete states.

The articular facet of the quadrate showed variation in its articulation with the pseudoangular and was summarized into three types: type 1, the anterior vertex was well developed and covered most of the articular facet of the pseudoangular (Figs. 5A–5B, 5D); type 2, the anterior vertex was reduced and covered only part of the articular facet (Fig. 5C), representing an extreme within the observed range; type 3, the anterior vertex was small and weakly projected, with variable orientation (Figs. 5E–5H, 5I–5J). In all specimens, the configuration of the articular facet was bilaterally symmetrical.

The retroarticular process of the pseudoangular varied in both curvature and relative length. Curvature referred specifically to the dorsal margin of the retroarticular process posterior to the processus condyloides, as seen in lateral view. Three shapes were recognized: shape A, dorsal and ventral margins convex (Figs. 5A–5C, 5E–5H); shape B, dorsal margin convex and ventral margin concave (Fig. 5D); and shape C, dorsal margin concave and ventral margin convex (Figs. 5I–5J).

Relative length of the retroarticular process was described using three types, assessed in lateral view to avoid projection artifacts between views: type 1, process reached approximately the posterior margin of the occipital condyle (Figs. 5A–5C, 5G, 5J); type 2, process did not reach that margin (Figs. 5D, 5I); and type 3, process extended distinctly beyond the posterior margin of the occipital condyle (Figs. 5E, 5F, 5H). Apparent discrepancies between lateral and dorsal views reflected differences in skull orientation rather than true asymmetry.

The paired frontals, viewed dorsally, showed four composite shapes integrating variation in the anterior and posterior margins and lateral divergence: shape A, the anterior margin bore a slight medial notch and the lateral margins were gently oblique (Figs. 6A–6C); shape B, the anterior margin also bore a slight medial notch but the lateral margins form well-defined posterior angles (Figs. 6D–6E); shape C, anterior margin was strongly angulated and projecting, with sharply divergent lateral margins bearing pointed processes (Figs. 6F–6H); shape D, both anterior and posterior margins showed very slight medial notches and the lateral margins were smoothly curved (Figs. 6I–6J). Although a more reductive approach, separating individual margins, was possible, the features described here covary consistently within the examined sample and were treated as composite characters, with the acknowledgement that some subjectivity is unavoidable given the limited sample size.

The os basale forming the palatal floor varied in shape and relative width: shape A, the cultriform process narrowed progressively; lateral borders were slightly concave from basipterygoid process and convex between the carotid foramen and basipterygoid process; the palatal border of the otic capsule was slightly wavy (Figs. 7A–7C); shape B, the cultriform process also narrows progressively, lateral borders were slightly concave throughout, and the palatal border of the otic capsule was weakly wavy (Figs. 7D, 7E); shape C, the cultriform process narrowed abruptly; lateral borders were slightly concave anteriorly and straight posteriorly, and the palatal border of the otic capsule was distinctly wavy (Figs. 7F–7H); shape D, the cultriform process narrows abruptly; lateral borders were slightly concave anteriorly and convex posteriorly, and both the palatal border of the otic capsule and lateral margins were rounded (Figs. 7I–7J). Overlap among some conditions (*e.g.*, Figs. 7B and 7D) reflected continuous variation, and the states defined here were intended as descriptive summaries rather than rigid categories.

The pterygoids exhibited four shapes: shape A, the pterygoid was small, with long and slender anterior processes (approximately two-thirds of total pterygoid length) contacting the maxillopalatine; dorsolateral processes were very close but not fused with the quadrate, and the area adjacent to the os basale was rounded and in posterior contact (Figs. 7A–7C); shape B, the pterygoid was small, with short and slender anterior processes (approximately one-fifth of total length), dorsolateral processes distant from the quadrate, and a rounded posterior in contact with the os basale (Figs. 7D, 7E); shape C, the pterygoid was large, with short anterior processes (one-fifth to one-third of total length), dorsolateral processes closely attached to the quadrates, and the area adjacent to the os basale slightly curved and nearly in contact (Figs. 7F–7H); shape D, the pterygoid was large and broadly attached to the maxillopalatine; dorsolateral processes were barely in contact with the quadrate, and the medial margin adjacent to the os basale was slightly emarginated and in posteromedial contact (Figs. 7I–7J). Variation in pterygoid morphology partly covaried with os basale shape, particularly in the area of contact.

The combined morphological data (Tables 3–5, Appendix II) for *Amazops* and *Epicrionops* expanded upon the cranial differences previously identified by Wilkinson, Reynolds & Jacobs (2021). These included differences in orbital enclosure by the maxillopalatine (complete *versus* partial), variation in length, curvature, and distal expansion of the squamosal postero-dorsal processes, and differences in the extent and configuration of contact between the maxillopalatine and quadrate. Additional variation was observed in the composite shape of the paired frontals, the relative proportions and articulation of the pterygoids, and annular counts, including fewer vent annuli (3–4 *vs* > 4) and fewer post-vent annuli (10–14 *vs* > 14). Although several of these characters showed overlap or continuous variation, the overall combination of cranial and postcranial features supported the distinction between *Amazops* and *Epicrionops*.

Although numerous osteological characters were described, only a subset consistently distinguished *Amazops* from *Epicrionops*, particularly those related to orbital enclosure, squamosal morphology, and quadrate–maxillopalatine relationships. Other observed variation should be interpreted cautiously, as it may reflect ontogenetic or intraspecific variation rather than fixed diagnostic differences. Accordingly, these characters are not interpreted as strictly discrete states but as structured descriptors of morphological continua within the sampled material.

### Species-level analyses

**Comparative external morphology.** Eastern species of *Epicrionops* showed distinctive combinations of meristic and proportional characters: *Epicrionops peruvianus* and *E. petersi noblei* had the highest counts of total annuli (367 and 316, respectively). Tail length relative to total length (**TL/TOL)** ranged from higher to lower values in the following order: *E. petersi noblei*, *E. p. petersi* (1.5–4.6), *E. lativittatus* (6.9), *E. bicolor subcaudalis* (6.5), *E. peruvianus* (6.3), and the specimens of *Epicrionops* sp. nov. from Southeastern Ecuador (5.1–6.1). The **BW2/TOL** ratios were: *E. p. petersi* (3.7–5.0), *E. lativittatus* (6.4), *Epicrionops* sp. nov. from Southeastern Ecuador (4.4–6.9), *E. p. noblei* (4.9), *E. bicolor subcaudalis* (4.8), and *E. peruvianus* (4.2).

*Epicrionops parkeri* had the highest number of **TA1,** and *E. columbianus* had the lowest. *Epicrionops bicolor subcaudalis* attained the highest number of **PVA,** and *E. columbianus* the lowest. The **VA** was highest in *E. marmoratus* and lowest in *E. lativittatus*. *Epicrionops marmoratus* had the greatest **TOL** and *E. columbianus* the smallest. *Epicrionops petersi noblei* had the shortest **TL**. Detailed morphological and morphometric data from Ecuadorian species are shown in Tables 3 and 4. These patterns indicate some overlap in meristic and proportional traits among species, limiting their diagnostic value when used in isolation.

**Colouration.** All examined *Amazops* specimens were uniformly dark, similar to *E. columbianus*, *E. peruvianus*, and *E. petersi. Epicrionops lativittatus*, *E. parkeri*, the type material of *E. bicolor,* and the specimens from the new species were all striped. The holotype of *E. marmoratus* is speckled. Our specimens from western Ecuador showed an apparently continuous variation from striped, mottled and speckled (see more information on the *E. bicolor-marmoratus* complex below). Overall, colouration patterns showed high variability and overlap among species, limiting their reliability as diagnostic characters when used in isolation.

### *Amazops amazops* Wilkinson, Reynolds & Jacobs, 2021

We examined five specimens that show characters mostly consistent with the holotype description, including a low number of **TA** (261–287 in dorsal count and 249–280 in ventral count, only slightly higher than the holotype with 247) and **PVA** (10–14, holotype 10). Two specimens (QCAZ 03210 from Cosanga and DHMECN 14152 from Sumak Kawsay) differ in having higher TA (TA1 292, TA2 298), and their specific identification as *A. amazops* requires further study. Two other specimens (MEPN 07801 and QCAZ 74837 from Jumandi) exhibit higher annuli counts, with TA exceeding 300 (**TA1** 319–350, **TA2** 310–347, **PVA** 12–13, and **VA** 5), differences in cranial osteology and phylogenetic distance (see below) suggesting that they may correspond to an undescribed species of *Amazops*.

Based on morphological or phylogenetic evidence, we assign the specimens DHMECN 15867, QCAZ-A 03210, 42405, 42407, 56870, 70228, 74837, and ZSFQ 01354 to *Amazops* (Fig. 7, Tables 2–5). *Amazops* has a broader distribution than previously recognized, with new records in the provinces of Napo and Pastaza. The specimens from the province of Pastaza (DHMECN 15867, QCAZ-A 42405, 56870, 70228, ZSFQ 01354) show morphological and osteological characters, including a low number of **PVA** (10–13), four Vent annuli, postero-dorsal processus of squamosal spear (shape C), quadrate not in contact and elongated (type E), frontal with starry shape, that are consistent with their identification as *Amazops amazops*.

### Epicrionops petersi

Northern populations of *E. petersi* (Napo and Pastaza) from Taylor (1968) and Taylor & Peters (1974) differed from those of Morona Santiago in having higher counts of total annuli (287–316, $\bar {x}$ =298.0 *vs.* 267–295, $\bar {x}$ =277) and principally lower counts of caudal annuli (<12, except JAP7101 *vs.* >16), resembling the general morphology of *Amazops*. The Loja population had higher counts in total annuli (318), 16 post-vent annuli and six to seven collar grooves. We consider that northern populations assigned by Taylor to *E. petersi* are more consistent with *Amazops* based on annular counts and cranial features, although this interpretation remains provisional pending examination of the material and broader sampling. A recent record of *E. petersi* from Colombia (Chaves-Portilla, López-Ordóñez & Fernández-Roldán, 2021) is more consistent with *Amazops*, based on the low number of post-vent annuli (*n* = 12) and vent annuli (*n* = 5).

Therefore, the currently available evidence suggests that *E. petersi* can be confidently confirmed only from its type locality, Agua Rica, Morona Santiago Province, Ecuador (Taylor, 1968), based on the holotype and paratopotypes, and from a second locality in the Cordillera del Cóndor, Tundayme, Zamora Chinchipe Province, represented by a specimen examined herein (QCAZ 76506). This second record is located approximately 65 km southeast of the type locality. Given the taxonomic uncertainty affecting several populations historically assigned to *Epicrionops*, we recommend re-examination of museum specimens currently identified as *E. petersi* to evaluate their identification.

### *Epicrionops bicolor-marmoratus* complex

The morphological dataset revealed extensive overlap among specimens from western Ecuador that would be identified as *E. bicolor* and *E. marmoratus* following Taylor (1968) and Taylor & Peters (1974), as well as among specimens reported in the literature (Cisneros-Heredia, 2004; Arteaga, Bustamante-Enríquez & Guayasamin, 2013; Ron, Merino-Viteri & Ortiz, 2024). We found no discrete meristic, morphometric or colouration character that unequivocally separated our specimens into the two taxa. **TA** counts and **PVA** fell within broadly overlapping ranges, and proportional measurements showed continuous variation without bimodal distributions or gaps suggestive of independent morphological clusters. Qualitative external features, including colouration patterns traditionally associated with these taxa, also appeared variable and inconsistently correlated with meristic differences.

Nine specimens from localities at altitudes between 515 and 2,400 m in the provinces of Santo Domingo de los Tsáchilas and Cotopaxi (northwestern Ecuador) showed a striped pattern (**TA1** 279–316; **TA2** 254–305). Six specimens from the same localities in Cotopaxi showed a mottled pattern (**TA1** 254–305, **TA2** 239–293). Two specimens (DHMECN 10828, MEPN 05198) from El Oro province (southwestern Ecuador), at elevations between 1,713 and 2,354 m, exhibited a striped pattern on a lighter brown background when compared with specimens from northwestern Ecuador (**TA1** 288–324, **TA2** 285–309). A specimen (DHMECN 19465) from the Reserva Drácula, at 2,400 m in the Carchi Province (extreme northwestern Ecuador, near the border with Colombia), showed a mottled pattern over a darker background (**TA1** 293, **TA2** 289). Three specimens from localities between 751 and 1,660 m in Imbabura, Pichincha, and Bolivar (north and central-western Ecuador) exhibited a speckled pattern (**TA1** 323–335; **TA2** 311–326). The striped specimens could tentatively be assigned to *E. bicolor*, but their apparent sympatry with mottled specimens (see below) led us to question whether two species co-occur in the same territories, or whether these specimens represent a single species exhibiting greater variation in colouration than previously reported by Taylor (1968) and Taylor & Peters (1974), considering that colouration has been treated as a key diagnostic character of *E. bicolor*. Conversely, the speckled specimens with high **TA** counts could tentatively be assigned to *E. marmoratus*. Notably, one of these specimens (QCAZ 08723) was collected at Junin, just a few kilometers from the type locality of *E. bicolor*.

The type locality of *E. bicolor* is Intag, at approximately 3,900 ft elevation (≈1,190 m), whereas the type locality of *E. marmoratus* is Santo Domingo de los Tsáchilas, at approximately 670 m elevation. This difference has historically suggested that *E. marmoratus* may represent a lowland species, whereas *E. bicolor* may be associated with higher elevations. However, our data do not support this pattern. The specimen from Junín, which shows the colouration pattern typically attributed to *E. marmoratus*, was collected at high elevation near Intag, whereas at least one striped specimen resembling *E. bicolor* was collected at Jardín de los Sueños (La Maná), at approximately 500 m in the lowlands. These observations indicate that colouration patterns did not consistently correspond to elevation and further support the interpretation of extensive phenotypic overlap between these taxa.

We examined 25 specimens of *Epicrionops* from western Ecuador, including all those held in the country’s major herpetological collections (QCAZ, ZSFQ, MZUTI, DHMECN and MEPN). Ten QCAZ specimens were reported as originating from the locality “Pampas and surroundings”, at 1,600 m, in the province of Cotopaxi, and were collected between 1987 and 1989 by “Giovanni Onore”. These specimens include both striped and mottled individuals. However, it has been evidenced that “Las Pampas”, or “San Francisco de Las Pampas” and its surroundings, is a locality of dubious certainty. Hundreds of specimens collected by the aforementioned collector and his collaborators were coded under the same locality, despite being collected at various, even distant, locations, at altitudes ranging from the lowlands at 600 m to montane forests at 2,000 m (Coloma, 1995; Cisneros-Heredia & Touzet, 2004). Notably, the two *Epicrionops* specimens (UMMZ 190478 and UMMZ 185825) that have been included in published phylogenies (Hass, Nussbaum & Maxson, 1993; Hedges, Nussbaum & Maxson, 1993; Hay et al., 1995; San Mauro et al., 2014; Maciel et al., 2018) are also reported as having been collected at the same locality, during the same period and by the same collector. They are separated by a *p*-uncorrected distance for the gene 16S rRNA of 2.36% suggesting that they represent separate species. Also, the specimens mentioned as “*Epicrionops lativittatus*” in the catalogue of the Muséum d’Histoire Naturelle de Genève also come from Las Pampas (MHNG, 2026).

Rather than forming clearly distinct morphological entities, these specimens were interspersed across morphological character space, indicating a pattern of morphological continuity. This lack of diagnostic exclusivity suggested either pronounced intraspecific variation or incomplete morphological differentiation among lineages and highlighted the need for integrative taxonomic assessment before maintaining them as clearly separable taxa based solely on external morphology. Therefore, pending further studies, we provisionally considered these specimens to represent a species complex, the *E. bicolor-marmoratus* complex. This pattern is consistent with scenarios of recent divergence, incomplete lineage sorting, or phenotypic plasticity, but cannot be resolved without additional genomic, taxonomic and ecological data. These findings further support the interpretation that traditional diagnostic characters are insufficient to reliably delimit these taxa.

Given the current taxonomic uncertainties surrounding *E. bicolor* and *E. marmoratus*, and for the purposes of diagnosing the new species presented below, we adopt a dual comparative framework. We first follow the original species definitions of *E. bicolor* and *E. marmoratus* as provided by Taylor (1968) and Taylor & Peters (1974) to establish formal diagnostic comparisons. In parallel, we include explicit comparisons with the specimens examined herein, which we treat as part of the *E. bicolor-marmoratus* complex.

### *Epicrionops* sp. from Southeastern Ecuador

The specimens collected at Río Negro-Sopladora National Park, Dave Goucher farm, Tapichalaca reserve, and Cordillera del Cóndor, showed morphological and osteological characteristics and phylogenetic positions indicating that they belong to the genus *Epicrionops* and represent an undescribed species that we describe in the following section.

## Systematic Account

**Table:** 

***Epicrionops jameskenti* sp. nov.**
**LSID: urn:lsid:zoobank.org:act:858A2D4A-11C8-4B2A-B771-78BE3112C2E5**
**English common name:** Río Negro Beaked Caecilian
**Spanish common name:** Cecilia hocicuda de Río Negro

**Holotype.** (Figs. 3, 8–10) Fully metamorphosed male (ZSFQ 04136, field series JCS-1675) collected at Ruta del Padre Albino, sector El Rosario, Parque Nacional Río Negro-Sopladora (−2.7724, −78.4776, 1,490 m), canton El Copal, Morona Santiago province, Ecuador, 03 March 2020, by Juan Carlos Sánchez-Nivicela, Juan Manuel Falcón and Tarquino Valverde.

**Figure 8:**
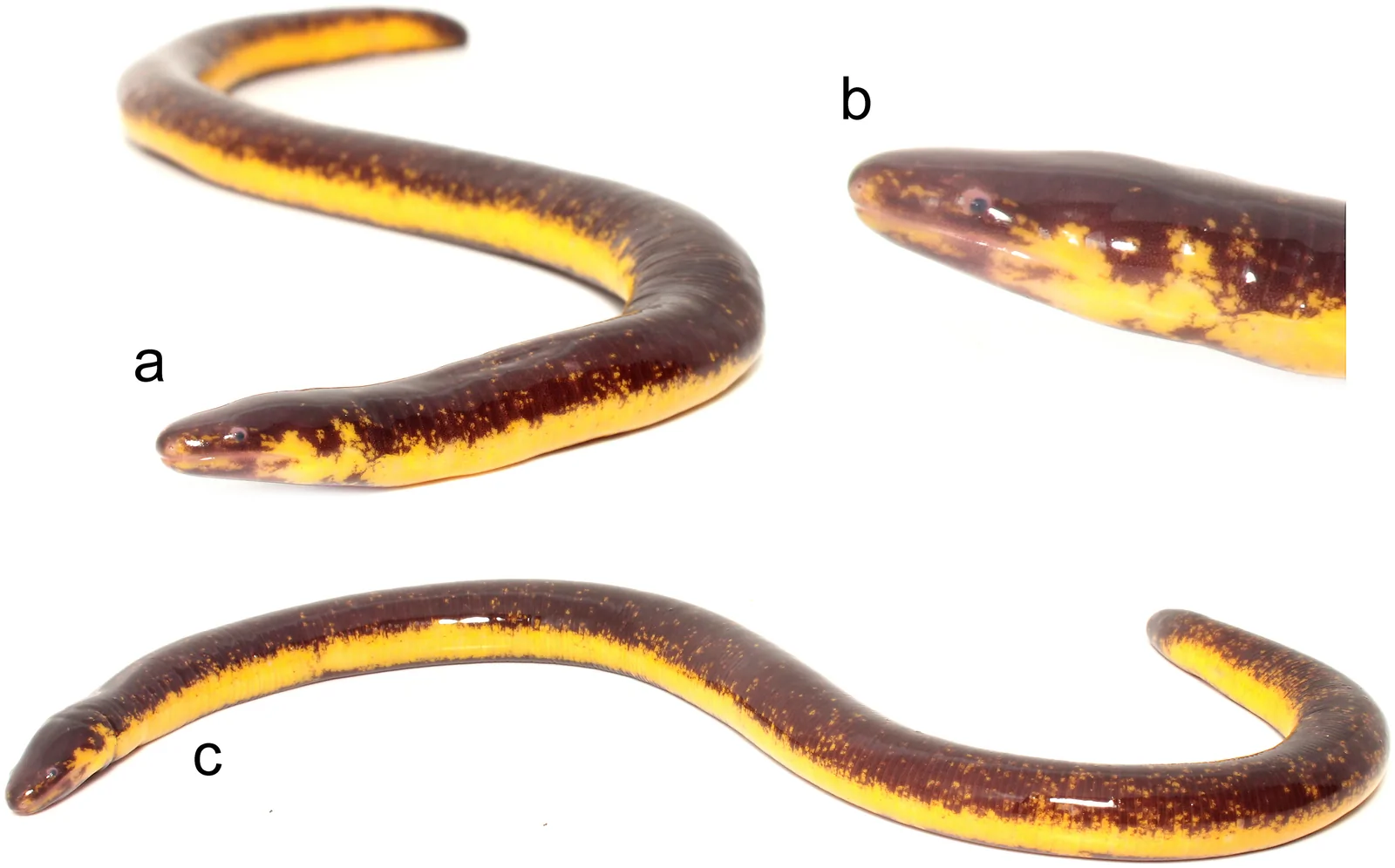
Holotype (ZSFQ 4136) of *Epicrionops jameskenti* sp. nov., male, in life. (A) Fronto-lateral view; (B) lateral view of head; (C) dorso-lateral view. Total length = 221 mm. Photographs by Juan C. Sánchez-Nivicela.

**Figure 9:**
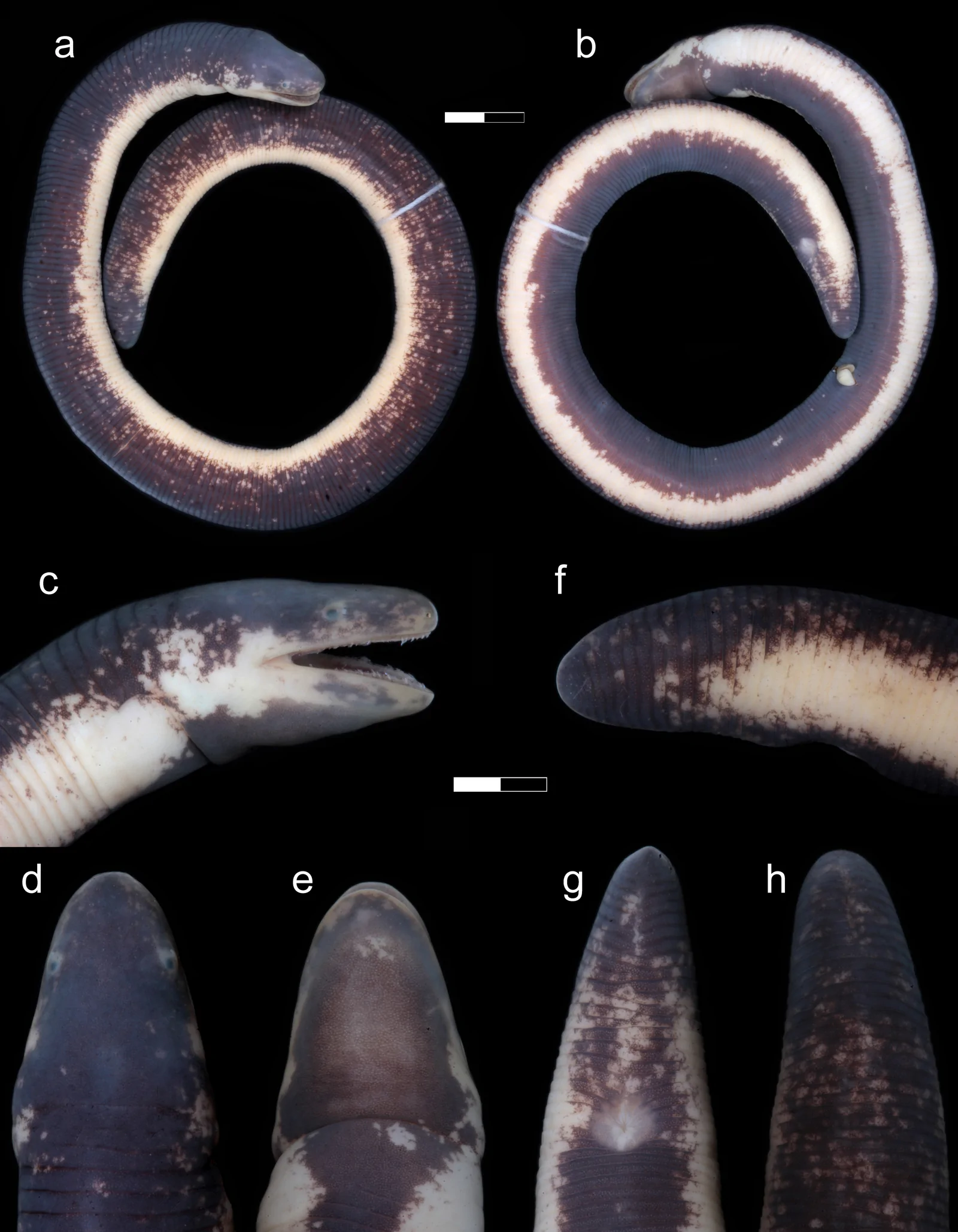
Holotype (ZSFQ 4136) of *Epicrionops jameskenti* sp. nov., male, in preservative. (A) Dorsolateral view, and (B) ventrolateral view (Scale bar = 10 mm). (C) lateral view of head, (D) dorsal view of head, (E) ventral view of head, (F) lateral view of tail, (G) ventral view of tail, and (H) dorsal view of tail (Scale bar = four mm). Photographs by Juan C. Sánchez-Nivicela.

**Paratypes.** (Figs. 11, 12) Eight paratypes: Adult female (QCAZ 79921), Concesión de Proyecto Lundingold “Emperador-Río Blanco” (−3.9101, −78.4892, 1,615 m), canton Yantzaza, parish Los Encuentros, Zamora Chinchipe province, Ecuador, 23 October 2024, by Valentina Posse; fully metamorphosed female **(**MZUA 02854), Dave Goucher farm (−3.1521, −78.5610, 1,600 m), canton San Juan Bosco, Morona Santiago province, Ecuador, 29 August 2018, by Henry Garzón; fully metamorphosed male (DHMECN 04600), Reserva Tapichalaca (−4.4729, −79.1469, 1,929 m), canton Palanda, Zamora Chinchipe province, Ecuador, 2007, by Juan Pablo Reyes; fully metamorphosed male (MUTPL 03998), and larva (MUTPL 03997), Centro Shuar Yawi (−3.1962, −78.2879, 1,030 m), parish San Antonio, canton Limón Indanza, Morona Santiago province, Ecuador, 27 April 2025, by Emili Ochoa; larvae (ZSFQ 04138, 04137), same data as holotype; larva (MZUA 02161), collected at the type locality, 15 July 2017, by Juan Carlos Sánchez-Nivicela and Eduardo Toral.

**Figure 10:**
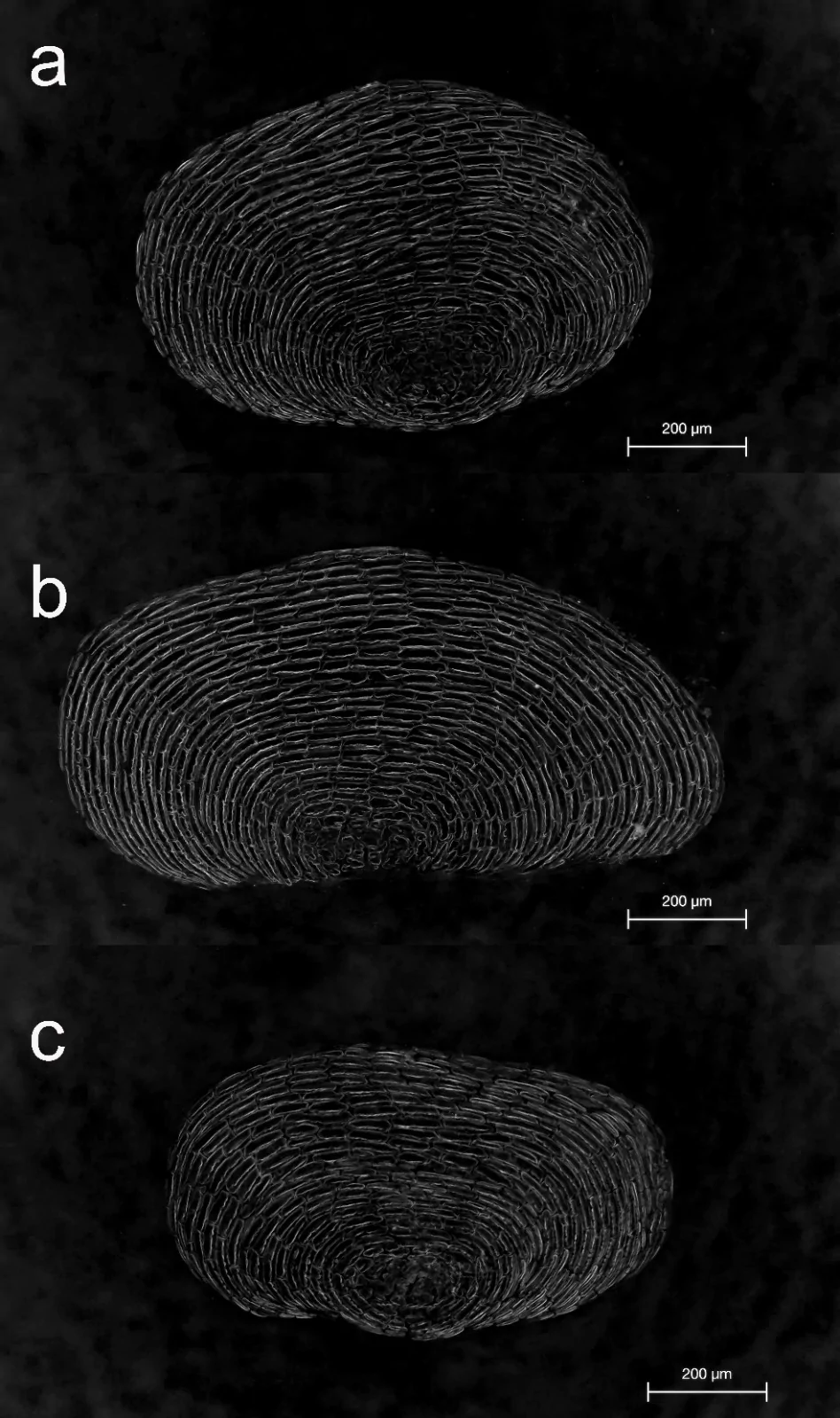
Details of the dermal scales of *Epicrionops jameskenti* sp. nov. (A) Anterior third scale, (B) middle scale, and (C) posterior third scale.

**Figure 11:**
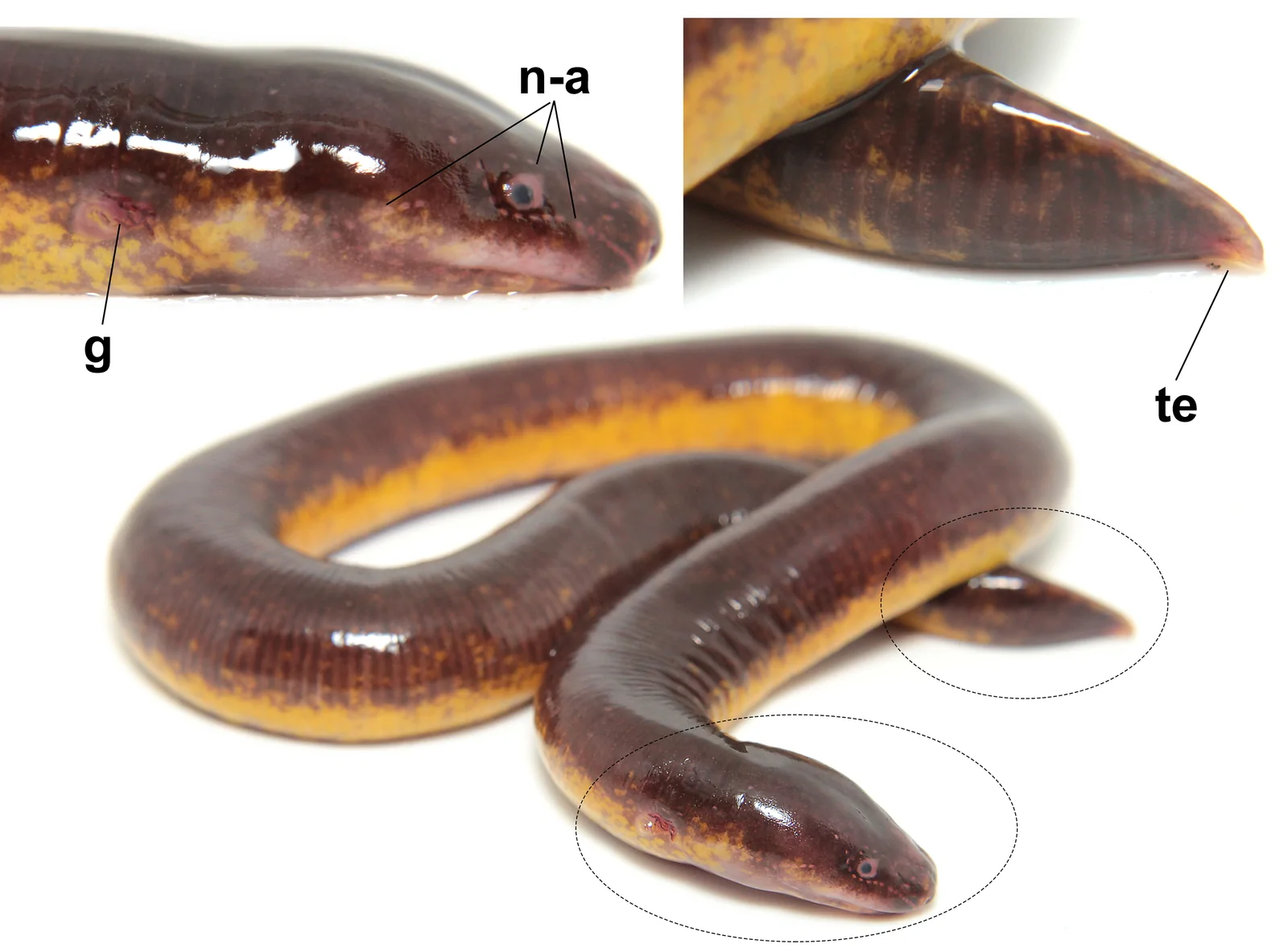
Details of the paratype larvae in stage 37 (ZSFQ 4137) of *Epicrionops jameskenti* sp. nov. Above left, lateral view of head; above right, lateral view of tail; below, fronto-lateral view of body. Larvae characters: (n–a) neuromast and ampullary organs, (g) external gills and gills slit, (te) tail fins. Total length = 189 mm. Photographs by Juan C. Sánchez-Nivicela.

**Figure 12:**
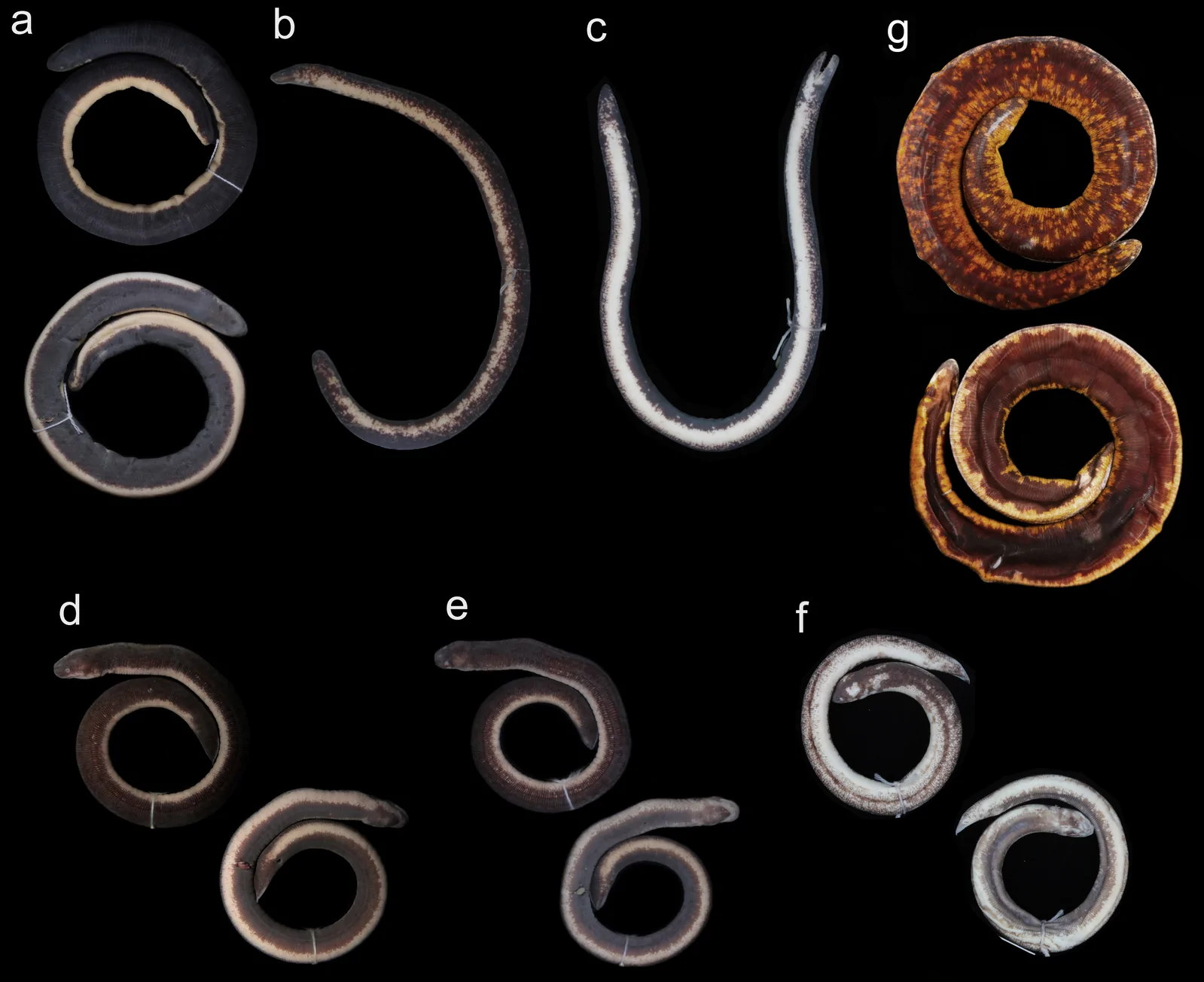
Dorsolateral (above) and ventrolateral (below) views of paratypes of *Epicrionops jameskenti* sp. nov., in preservative. (A) MZUA AN. 2854, fully metamorphosed female (TOL = 215 mm), (B) DHMECN 4600, fully metamorphosed male (TOL = 174 mm), (C) MUTPL 3998 fully metamorphosed male (TOL = 210 mm), (D) ZSFQ 4137, stage 37 larvae (TOL = 184 mm), (E) ZSFQ 4138, stage 37 larvae (TOL = 183 mm), (F) MUTPL 3997 stage 36 larvae (TOL = 154 mm), (G) QCAZ 79921, adult female (TOL = 317 mm). Photographs by Juan C. Sánchez-Nivicela, and Gabriel Morales (QCAZ 79921).

**Diagnosis.** *Epicrionops jameskenti* sp. nov. differs from all other species of *Epicrionops* by the following combination of characters: (1) medium-sized body (**TOL** 174–317 mm; **BW2/TOL** 4.4–6.9%, $\bar {x}$ 5.2, SD 1.0); (2) relatively short tail (**TL/TOL** 5.1–6.1%, $\bar {x}$ 5.4, SD 0.4); (3) short snout (**EN/HL** 31.8–42.0%, $\bar {x}$ 34.9, SD 4.2); (4) vent longitudinal with denticulate margins, with 5–7 vent annuli interrupted by the vent; (5) scales present in the collars, arranged in one anterior row, two middle rows and one posterior row, with elliptical scales wider than long; (6) postero-dorsal process of the squamosal long and spatulate, with the squamosal-maxillopalatine suture extending from the middle of the anteroventral margin and angular in outline (shape D, type 3); (7) quadrate in dorsal contact with the posterior end of the maxillopalatine, without contact between this element and the squamosal, and with the articular facet bearing a small, weakly projected anterior process directed anteriorly (shape D, type 3); (8) pseudoangular with dorsal margin concave and ventral margin convex in lateral view, with retroarticular process reaching or slightly exceeding the posterior edge of the occipital condyle (shape C, type 1, 2); (9) frontals with anterior and posterior margins bearing a very shallow medial notch and gently curved lateral margins (shape D); (10) os basale with an abruptly narrowing cultriform process, lateral margins slightly concave anterior to the basipterygoid process and convex between the carotid foramen and basipterygoid process, and rounded palatal margins of the otic capsules (shape D); (11) pterygoids large, firmly attached to maxillopalatine, with dorsolateral processes weakly contacting but not fused to the quadrate, and a posteromedial area contacting the os basale (shape D); (12) orbits large and rounded, occupying approximately one fifth of the maxillopalatine length; (13) teeth formula: **PMT** = 15–16, **PPT** = 17–18, **DT** = 16–16, **ST** = 9–10; (14) elliptical scales around the body, wider than long; and (15) distinctive bicoloured pattern with yellow lateral bands alternating with brown dorsal and ventral bands along most of the body, with reduced yellow speckling on head, dorsum, and tail (Figs. 3, 8–10).

*Epicrionops jameskenti* sp. nov. differs from *E. columbianus, E. peruvianus* and *E. petersi* by its striped colour pattern (uniformly dark in those species), from *E. marmoratus* by having a striped pattern (mottled pattern in the former). It can be distinguished by having *E. marmoratus* (339 in the holotype), and *E. parkeri* (326 in the holotype). The number of post-vent annuli (PVA = 16–20) overlaps as its lower limit with *E. p. petersi* (16), and lower than *E. peruvianus* (23 in the holotype), *E. marmoratus* (21 in the holotype). It also has a higher number of vent annuli (**VA =** 5–7) than *E. lativittatus* (4) and narrowly overlaps *E. b. bicolor* (5 in the holotype). By its relative length of the tail of **TL/TOL** 5.1–6.1% ($\bar {x}$ 5.4, SD 0.4), it can be distinguished from *E. p. noblei* (1.5–2.1%, $\bar {x}$ 1.8, SD 0.4), *E. bicolor-marmoratus* complex (1.4–6.0%, $\bar {x}$ 4.4, SD 1.5), *E. lativittatus* (6.9%) (Table 4). Cranial osteological differences among Ecuadorian species of *Epicrionops* are summarised in Table 5. *Epicrionops jameskenti* sp. nov. has a thin lateral stripe between 3.6–3.8 mm in width, while *E. lativittatus* has a thick lateral stripe (5.5–7 mm), and the ending of the lateral stripe in *E. jameskenti* sp. nov. extends beyond the limit of the vent and exceeds 2/3 of the tail length, whereas in *E. lativittatus* the stripe unite at the front of the vent and in *E. b. subcaudalis* the stripes terminate at the level of the posterior end of the vent. *Epicrionops jameskenti* sp. nov. shows a lower premaxillary-maxillary tooth count (15/16) than *E. p. petersi* (18/20).

Scales in *E. jameskenti* sp. nov. are elliptical throughout the body and consistently wider than long (anterior: 0.9 × 0.6 mm; midbody: 1.1 × 0.6 mm; posterior: 0.9 × 0.5 mm). In contrast, scales in *E. b. bicolor* are longer than wide (type, BMNH 78.1.25.48: 1.05 × 1.25 mm; 1.0 × 1.2 mm). Scales in *E. b. subcaudalis* are variable in shape and obliquely oriented depending on body region (type, AMNH 17305; largest scale 1.56 × 2.0 mm). In *E. lativittatus*, scales are oblique and nearly as long as wide (type, AMNH 46205; 1.65 × 1.8 mm). Scales in *E. marmoratus* show regional differentiation, with anterior scales larger and laterally expanded, whereas posterior scales are oblique and nearly as wide as long (type, BMNH 1956.1.17.87: 1.5 × 2.2 mm; 1.3 × 2.2 mm). In *E. peruvianus*, scales are predominantly elliptical (type, BMNH 1902.5.29.207; largest scale 1.3 × 2.0 mm). Scales in *E. parkeri* are oblique, ranging from longer than wide to approximately as long as wide (type, BMNH 97.11.12.23: 0.9 × 1.0 mm; 0.8 × 1.25 mm). In *E. p. petersi*, scales are polygonal and nearly as wide as long (JAP 5979: 1.6 × 1.98 mm; 2.05 × 2.5 mm), a condition also observed in *E. p. noblei* (type, AMNH 1454; largest scale 1.4 × 1.95 mm) (Taylor, 1972).

Comparative proportional relationships among head measurements (Table 3) further support differentiation between *Epicrionops jameskenti* sp. nov. and *E. p. petersi*, indicating differences in head shape and relative structure. In *E. jameskenti* sp. nov., the ranges of head width (**HW1**: 6.1–10.2 mm; **HW2**: 6.6–10.4 mm) and head height (**HH2**: 4.6–6.6 mm) are proportionally smaller relative to dimensions in *E. p. petersi* (QCAZ 76506: **HW1** = 10.8 mm; **HW2** = 11.2 mm; **HH2** = 7.5 mm, JAP 4077, 7096–7101: **HW** = 8.5–12.0 mm), suggesting a comparatively narrower and dorsoventrally compressed head. Similarly, distances associated with sensory and feeding structures, including **EJ** (3.8–6.2 mm *vs.* 6.9 mm), **TLP** (0.7–1.5 mm *vs.* 1.6 mm), and **NLP** (0.6–0.9 mm *vs.* 1.1 mm), are consistently reduced in the new species, reinforcing a more compact cranial configuration. Additional proportional differences are evident in mandibular and collar-associated measurements: **LN** (9.1–13.3 mm *vs.* 14.9 mm), **LJL** (7.5–11.6 mm *vs.* 12 mm), and collar lengths (**C1**: 1.4–2.2 mm *vs.* 2.4 mm; **C2**: 4 –6.7 mm *vs.* 7.6 mm) are all relatively smaller in *E. jameskenti* sp. nov. Taken together, these non-overlapping or minimally overlapping proportional ranges indicate that *E. jameskenti* sp. nov. possesses a proportionally smaller head compared to *E. petersi*.

**Description of the holotype (Figs. 3, 8–10)**. Fully metamorphosed male; mouth preserved open. **TOL** 211 mm; **TA1** 291. Body subcylindrical, slightly dorsoventrally compressed anteriorly (**BW2**/**BH** 1.2, **CM** 32 mm), becoming less compressed posteriorly (**BW3**/**BH** 0.9); orthoplicate grooves; dorsal annuli well defined; nuchal region with three grooves: first nuchal groove faint dorsally and not visible ventrally, second nuchal groove visible ventrally only, third nuchal groove complete; nuchal collars conspicuous and thickened, first nuchal collar shorter (2.2 mm) than second (4.0 mm); four transverse dorsal grooves present on the second collar, bowing slightly anteromedially and terminating dorsolaterally; posterior to collars, 284 dorsal annuli. Scales present in the collars in discrete rows within annuli, one row anteriorly, two rows in middle and one row posteriorly, elliptical to weakly polygonal scales, wider than long, embedded in the dermis, and do not differ significantly in shape and size (the bigger scales in mm, anterior: width = 0.9, length = 0.6; middle: width = 1.1, length = 0.6; posterior: width = 0.9, length = 0.5), tail moderately short (**TL** 10.8 mm) with 19 **PVA**, slightly laterally compressed (**TAW/BW3** 0.8), tapering more strongly in dorsal than in lateral view, tail tip broadly blunt in lateral view, dorsal and ventral margins symmetrical in lateral view, ventral surface not flat; vent longitudinal with denticulate lateral margins, surrounded by 7 **VA**.

Head short (**HL** 10.8 mm), U-shaped in dorsal view, dorsoventrally compressed (**HW1/HH2** 1.3), approximately as wide and deep as adjacent nuchal region posteriorly (**HW1/HW2** 0.8, **HH2/HH1** 1.0), narrowing gently anteriorly to about a fifth of its width (**HW1/IND** 4.6); the lower jaws are almost as wide as the head in ventral view, rounded tip, mouth is marginally subterminal (**LJL** 8.0 mm); head tapers very gently anterior to eye level, sharply from level of nares; lips straight, slightly downturned at corner of mouth; corner of mouth slightly further from top than from bottom of head; lower jaws robust, deep as upper jaws; eyes are small and circular (**ED** 0.6 mm), black with grey periphery, and tiny tentacle in the anterior margin of the eye orbit, visible through skin, elevated above adjacent skin, equidistant between the head top and lip in lateral view, and completely noticeable in dorsal view; naris small (**ND** 0.3 mm), subcircular, notable in dorsal view, almost following the contour of the snout, slightly closer to tip than to top or bottom of snout, closer to lips (**NPL** 0.7 mm) than are eyes or tentacles (**TLP** 1.3 mm). Distance between nostrils (**IND** 1.6 mm) slightly less than half the distance from nostril to eye (**EN** 3.4 mm). Tongue triangular with acuminate tip; lingual and palatal mucosa pale and plicae, without papillae; choanae elongate, longer than wide; distance between choanae approximately 2.5 times their transverse diameter.

Based on the CT scan, skull zygokrotaphic; premaxillae and nasals unfused; squamosals not contacting frontals; orbits rounded, fully enclosed by the maxillopalatine and occupying approximately one fifth of its length, positioned slightly posteriorly relative to the maxillopalatine margins; foramen for tentacular duct small, about a third of the orbit, parallel to the center of the orbit; choanae elongate, longer than wide; distance between choanae 2.5 times their transverse diameter; dorsal edge of squamosal curved and posterior edge are concave, with postero-dorsal processus spatulated and the sutures between maxillopalatine and the squamosal joined from the middle of the anteroventral border (shape D, type 3); quadrate in superior contact with the posterior end of the maxillopalatine bone, with no contact between this end and the squamosal bone, and the articular facet of the quadrate with anterior vertex small, barely projected, directed anteriorly (shape D, type 3); processus retroarticularis in dorsal profile concave, in ventral profile convex, and does not reach the posterior edge of the occipital condyle (shape C, type 2); frontals with anterior and posterior margin with a very slight medial notch, and curved lateral margins (shape D); os basale abrupt narrowing of the cultriform process, lateral borders of the os basale slightly concave from the basipterygoid process and convex between the carotid foramen and basipterygoid process, the palatal border of the otic capsule and lateral borders rounded (shape D); pterygoids large and attached to maxillopalatine, dorsolateral processes barely in contact with quadrate, not fused, area adjacent to os basale slightly concave and in contact posteromedially (shape D).

Teeth oriented posteriorly, not strongly recurved, between 30° to 45° from the jaws, bicuspid, anterior and posterior teeth smaller than those in between, none hypertrophied; maxillopalatine teeth larger and more robust than premaxillary and prevomerine teeth; dentary teeth larger and more robust than splenial teeth; premaxillary teeth 4–4, maxillary teeth 11–12 (**PMT** 15–16); prevomer teeth 11–9, palatine teeth 7–8 (**PPT** 18–17); **DT** 16–16; **ST** 10–9; **VN** 75–79.

**Colouration of holotype in life.** Longitudinally oriented bicolour pattern, with a middorsal region with a thick dark brown band with yellow splashes, two thick dark yellow ventro-lateral bands and irregular edges that are lost on the tail. Venter brown; tail brown with yellow splashes; lips and around the eyes yellowish pink (Figs. 2 and 8). The ventrolateral bands are 3.6–3.7 mm wide.

**Colouration of holotype in preservative.** Middorsal region on head, body and tail, dark greyish brown with pinkish cream splashes. Ventrolateral stripes on the head, body and tail are pinkish cream. Tip of tail in dorsal, ventral and lateral views dark greyish brown (Fig. 9).

**Etymology.** The specific epithet of this new taxon, *jameskenti*, is given named in honours of Mr James Kent as a gift by our benefactress Anne Dunne, who funded this research.

**Larva (**Fig. 11**)**. Four larvae at early developmental stages, equivalent to the Stage 36 (MUTPL 03997, 154 mm **TOL**), Stage 37 (ZSFQ 04138–04137, 183–184 mm **TOL**) and Stage 38 (MZUA 02161, 213 mm **TOL**) in Dünker, Wake & Olson (2000), with reduction of the external gills, in MUTPL 03997 still visible externally, in ZSFQ 04136, 04137, and MZUA 02161 are degenerated and internalised in the gill chamber. Neuromast and ampullary organs were still noticeable in the head (nasal, supra, infra, postorbitals, supraspiracular, mandibular, and oral); greatly reduced in stages 37–38, a single row of trunk neuromasts in a specimen of stage 36, which approximately reached the first third. MUTPL 03997 with tail fins, extended from the sixth post-vent annuli; tail fins reduced in ZSFQ 04136, 04137, more rounded, less broad, extended from the ninth post-vent annuli, poorly defined and less extended on the ventral; narrow tail fin curved dorsally in MZUA 02161, poorly defined at the end of the tail and on the ventral, extending from the fourteenth post-vent annuli.

**Variation (**Fig. 12**)**. Fully metamorphosed female, MZUA AN.2854, solid bicoloured, the middorsal and ventral stripes darker than the other specimens; fully metamorphosed males, DHMECN 4600 and MUTPL 3998, bicoloured and the middorsal stripe brown with yellow splashes, more densely than the ventral stripes. The adult female, QCAZ 79921, is more yellowish than the other specimens, the lateral bands have a more yellowish tone and the middorsal stripe is not clearly distinguishable and a mostly mottled pattern; the ventral stripe is clearly distinguishable in brown; the lateral regions of the commissures of the mouth and vent are pale pink. Larvae ZSFQ 4138 and ZSFQ 4137, bicoloured and the middorsal stripe brown with yellow splashes, more densely than the ventral stripes. The larva MUTPL 03997 is less pigmented, but the darker colour pattern on the back and belly is recognizable. Dorsally, it becomes a more solid dark brown and is seen as a splash from the first anterior third.

**Distribution, natural history and conservation status (Figs. 13–14).** *Epicrionops jameskenti* sp. nov. is known from the southeastern slopes of the Eastern Mountain Range (Cordillera Oriental) of the Andes of Ecuador, in the provinces of Morona Santiago and Zamora Chinchipe. The species is known from five localities: Parque Nacional Río Negro-Sopladora, Dave Goucher Farm, Centro Shuar Yawi, Los Encuentros, and Reserva Tapichalaca.

**Figure 13:**
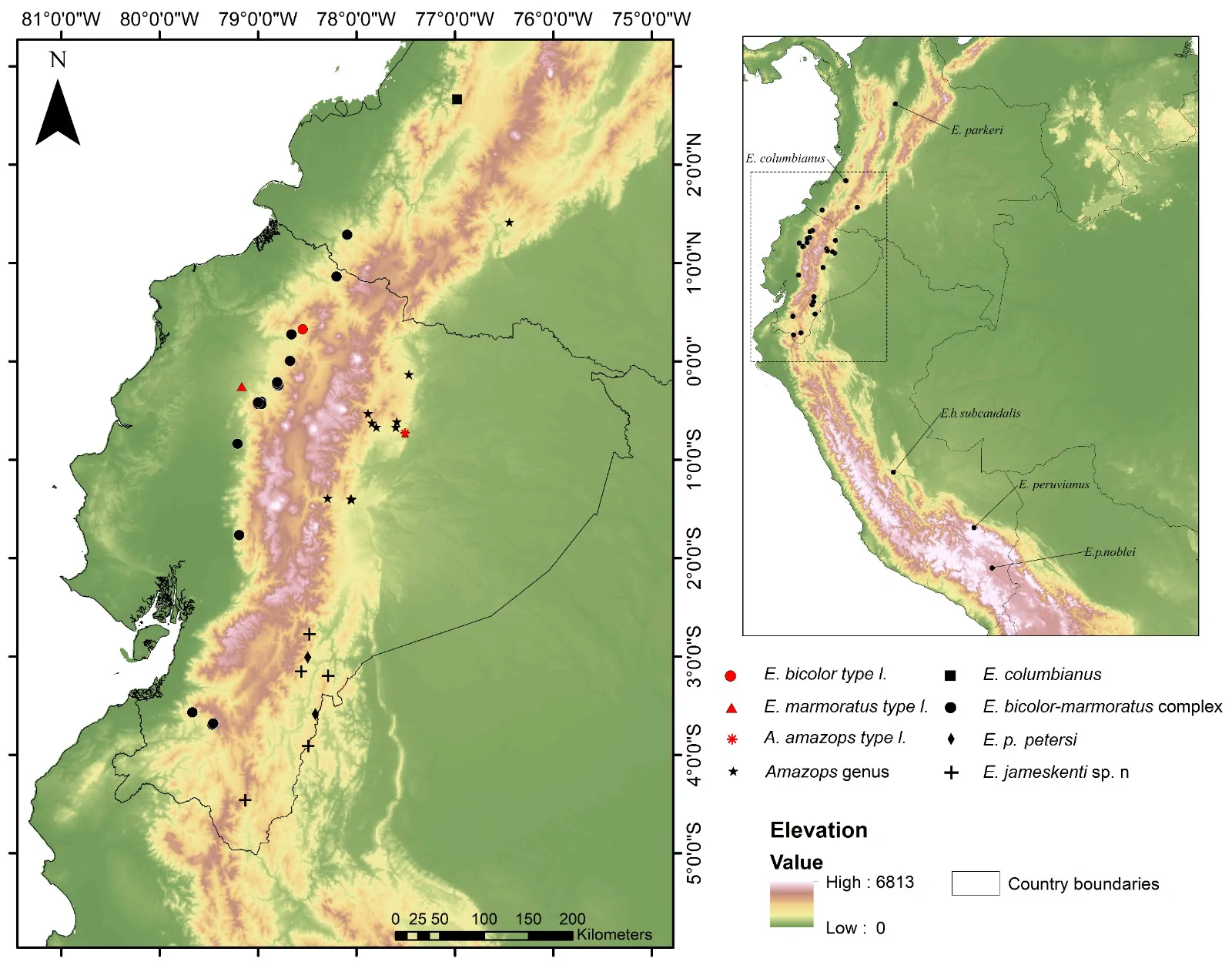
Distribution map of the genera *Epicrionops* and *Amazops* in Colombia, Ecuador and Peru. Area enclosed by dashed lines in the right map is enlarged in the left map. The red marks are the type localities. *Epicrionops jameskenti* sp. nov. from Morona Santiago and Zamora Chinchipe provinces, Ecuador (black cross).

**Figure 14:**
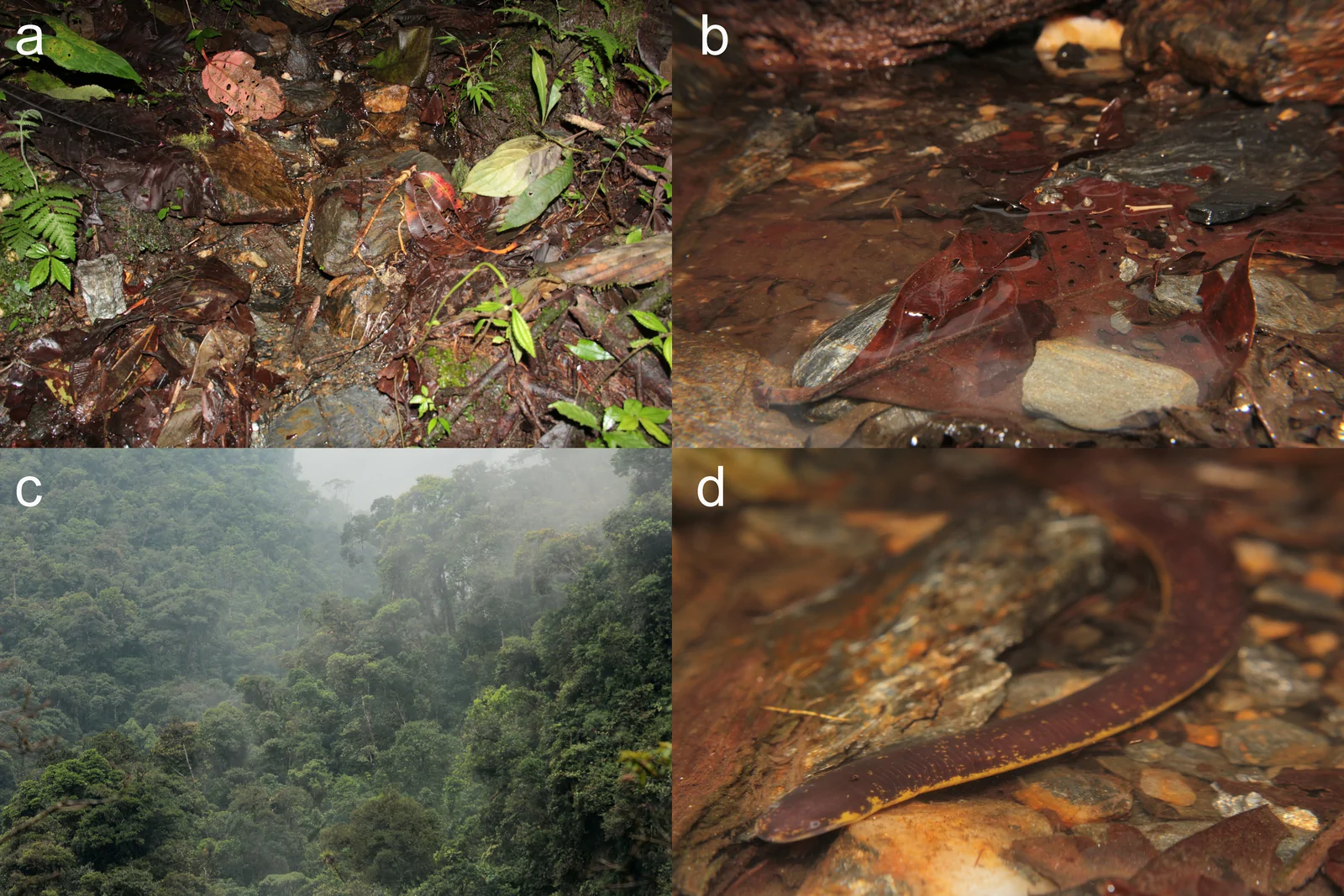
Habitat of *Epicrionops jameskenti* sp. nov. (A, B) Small stream with rocks and leaves in which the types were found; (C) the cloud forest where *Epicrionops* has its habitat. (D) holotype (ZSFQ 4136) swimming at the time of capture. Photographs by Juan C. Sánchez-Nivicela & José Manuel Falcón.

At the type locality of *E. jameskenti* sp. nov., three larvae and one adult were recorded during approximately two person-hours of search effort, suggesting that larval detectability may be higher due to their aquatic habits. The holotype and larvae (ZSFQ 04137, 04138, MZUA 02161) were found at 21:00, in a small stream inside the forest on the main trail called Ruta del Padre Albino, in the El Rosario sector of Río Negro-Sopladora National Park. All individuals were found in the water along a stream, between rocks and logs, and among litter accumulated at the edges. The stream was small, with a maximum depth of 40 cm and with a width not exceeding 100 cm (Fig. 14). MZUA 02854 was found dead in a small stream that crosses a third-order path, next to a forest in a private conservation area (Dave Groucher Farm), and this small stream did not exceed 30 cm in depth and 70 cm in width. DHMECN 04600 was found on a trail under a log, near a pasture inside the forest, with no nearby streams or rivers. MUTPL 03998 and 03997 were found at midday, on a very sunny day after torrential rain the day before, in a swampy area. MUTPL 03997 was found in a small pool of water about 15 m from a small stream, and MUTPL 03998 was about 10 m from a river. QCAZ 79921 was found on a path inside a forest at 10:56 p.m., moving among the leaf litter after a day of heavy rain.

Population size and demographic trends are unknown. However, we suggest that its association with stream habitats may make it vulnerable to deforestation, mining, pollution, climate change, and loss of aquatic integrity, which could affect its conservation status. Given the uncertainties about its population status, geographic distribution and ecology, we suggest that *E. jameskenti* sp. nov. could be considered under the IUCN’s Data Deficient category.

**Remarks.** Taylor & Peters (1974) reported one larva from Morona Santiago (JAP 6690) with similar colour patterns to *E. jameskenti* sp. nov., whose information from its collection locality coincides with the local names very close to the type locality of *E. jameskenti* sp. nov. and for this reason, we tentatively assigned this specimen to the new species.

## Discussion

We assembled and examined the largest available set of *Epicrionops* and *Amazops* specimens currently housed in Ecuadorian collections, substantially expanding the empirical basis for understanding morphological variation, geographic distribution, and taxonomic diversity within these genera. In total, we examined three adults, six fully metamorphosed individuals, and four larvae of *Amazops* and twenty adults, eight fully metamorphosed individuals and nine larvae of *Epicrionops* from Ecuador.

Our review expands the known distribution of *Amazops amazops* and provides new evidence that the genus is more widespread in the Ecuadorian Amazon than previously recognized. A key outcome of our study is the reassessment of specimens previously assigned to *Epicrionops petersi*. Several of these specimens, particularly from northern Amazonian localities and recent records from Colombia, are more consistent with *Amazops* based on the number of post-vent annuli and vent annuli. This suggests that the distribution of *E. petersi* may have been overestimated and that some historical records require re-evaluation. These results highlight the importance of revisiting museum collections using updated diagnostic frameworks. In addition, both morphological and molecular evidence suggest the presence of one potentially undescribed species of *Amazops*. These findings indicate that the diversity of the genus has likely been underestimated and that its current taxonomic representation remains incomplete. However, these interpretations must be considered in light of the absence of direct comparisons with all name-bearing type specimens, which may affect the accuracy of some historical identifications and underscores the need for future revisionary work incorporating type material.

Our findings underscore the critical role of museum collections as reservoirs of undocumented biodiversity. The reidentification of specimens and the recognition of previously unappreciated variation highlight how existing collections can yield novel taxonomic and biogeographic insights when examined using integrative approaches. In groups such as caecilians, where field detection is difficult and specimens are scarce, museum-based research remains an essential component of biodiversity discovery and taxonomic revision.

Our results also reveal substantial taxonomic complexity within *Epicrionops* from western Ecuador. The *E. bicolor–marmoratus* complex exemplifies the difficulty of diagnosing species using external morphology alone. Extensive overlap in colouration patterns, annular counts, and morphometric proportions prevents the identification of discrete diagnostic characters and suggests either high intraspecific variation or incomplete morphological differentiation among lineages. These findings call into question the reliability of some traditional diagnostic characters and may have implications for the validity of subspecies designations proposed by Taylor (1968) and Taylor & Peters (1974).

More broadly, most rhinatrematids exhibit colour patterns combining yellow and brown tones, including members of the genera *Rhinatrema* and *Epicrionops*, whereas uniform dark colouration occurs in *Amazops* and some species of *Epicrionops* (Taylor, 1968; Maciel et al., 2018; Wilkinson, Reynolds & Jacobs, 2021). However, colouration appears to be variable within species and may slightly change with size and ontogeny. High convergence in colour patterns, together with overlapping annulus range counts, contributes to uncertainty in species delimitation within *Epicrionops* (Müller, 2007; Yánez-Muñoz et al., 2007) and may also call into question the validity of some subspecies designations (Taylor, 1968; Taylor & Peters, 1974). Our results reinforce that even relatively large samples may be insufficient to establish species boundaries based solely on external morphology.

In this context, osteological characters provide an important and relatively underutilized source of taxonomic information (Wilkinson, Reynolds & Jacobs, 2021). Several cranial traits differ between *Amazops* and *Epicrionops*, including the maxillopalatine not fully enclosing the orbit, which provides a useful diagnostic distinction between these genera (Wilkinson, Reynolds & Jacobs, 2021). In addition, variation in the shape of the squamosal, quadrate, pseudoangular, frontal, os basale, and pterygoid may help distinguish among some Ecuadorian species of *Epicrionops*. These features are particularly informative in groups where external morphology is highly conserved. The description of *Epicrionops jameskenti* sp. nov. highlights the value of integrating osteological data into species diagnoses and expands the known diversity of the genus in eastern Ecuador, where only *E. petersi* had previously been reported (Nussbaum, 1977). We emphasize that the osteological characters used here are intended as comparative descriptors rather than discrete phylogenetic characters, and their diagnostic value should be interpreted cautiously given the limited sample size and potential for intraspecific variation.

Our molecular phylogeny provides a preliminary framework for interpreting these patterns. The topology suggests potential paraphyly of *Epicrionops* with respect to *Amazops*. However, this result must be interpreted with caution because our analysis includes only a small fraction of the known diversity of *Epicrionops*. Limited taxon sampling is well known to affect the recovery of monophyly and may produce artefactual relationships. The phylogenetic position of *Epicrionops parkeri* is weakly supported, and additional sequence data may recover a monophyletic *Epicrionops* as sister to *Amazops*. Alternative rooting scenarios further influence these relationships, and previous work has suggested that *Amazops* may represent the sister lineage to all other rhinatrematids (Wilkinson, Reynolds & Jacobs, 2021). Given these uncertainties, we refrain from proposing taxonomic changes at this stage.

Our findings have several taxonomic implications. First, they support the need for a reassessment of species boundaries within *Epicrionops*, particularly in light of the extensive morphological overlap observed among traditionally recognized taxa. Second, they indicate that some currently recognized species and subspecies may not represent discrete evolutionary lineages. Third, they highlight the importance of re-evaluating historical material and type series to ensure consistent species identification across the geographic range of the group.

At the same time, interpretations of morphological and phylogenetic patterns in Rhinatrematidae remain constrained by several limitations. Taxon sampling remains incomplete, sample sizes are small for most species, and geographic coverage is uneven. In particular, the absence of molecular data for several described *Epicrionops* specie*s* limits our ability to robustly assess monophyly and species boundaries. Furthermore, most specimens available in Ecuadorian collections have been obtained opportunistically rather than through systematic surveys.

Overall, this study highlights both the taxonomic diversity of *Epicrionops* and *Amazops* in Ecuador and the persistent challenges associated with species-level identification in rhinatrematid caecilians. By integrating external morphology, osteology, and molecular data, we provide an updated framework for understanding variation within these genera. Resolving species boundaries and phylogenetic relationships will require expanded geographic sampling, genomic-scale datasets, and direct examination of name-bearing types and specimens from type localities.

## Conclusions

We describe *Epicrionops jameskenti* sp. nov., a new rhinatrematid caecilian from the southeastern Andes of Ecuador, based on a unique combination of external morphology, cranial osteology, colouration, distribution and molecular characters. Our review of Ecuadorian material also expands the known distribution of *Amazops amazops*, supports the reassignment of several specimens previously identified as *E. petersi* to *Amazops*, and suggests that both *Epicrionops* and *Amazops* include additional undescribed diversity. More broadly, our results highlight the taxonomic complexity of rhinatrematid caecilians in Ecuador and the importance of integrating museum collections, osteology, and molecular data to resolve species boundaries and phylogenetic relationships.

## Supplemental Information

10.7717/peerj.21786/supp-1 Supplemental Information 1 Examined specimens and locality coordinates

10.7717/peerj.21786/supp-2 Supplemental Information 2 Counts and measurements of *Epicrionops* and *Amazops* All measurements in mm. Acronyms from Wei et al. (2025): Total length (TOL), Snout-vent length (SVL), Trunk length (TRL), Tail length (TL), Body width 1 (BW1), Body width 2 (BW2), Body width 3 (BW3), Body width 4 (BW4), Body height (BH), Head length (HL), Head width 1 (HW1), Head width 2 (HW2), Head height 1 (HH1), Head height 2 (HH2), Snout to 1st collar groove (STFG), Snout to 2nd collar groove (STSG), Snout to 3rd collar groove (STTG), Interorbital distance (IOD), Eye diameter (ED), Eye-nostril distance (EN), Eye-jaw distance (EJ), Snout-eye distance (SE), Tentacle-lip distance (TLP), Internostril distance (IND), Nostril-snout distance (NS), Nostril-jaw distance (NJ), Naris diameter (ND), Naris to margin of lip distance (NLP), Naris to nuchal groove distance (NFNG), Snout-jaw distance (SJ), Lower jaw to nuchal groove (LN), Lower jaw length (LJL), Jaw to nuchal groove distance (JNG), Tail width at posterior vent (TAW), Vent annuli (VA), Anal disc diameter (ADD), 1st collar length (C1), 2nd collar length (C2), Circumference at midbody (CM), Total annuli 1 (TA1), Total annuli 2 (TA2), Post-vent annuli (PVA).

## Additional Information and Declarations

### Field Study Permissions

The following information was supplied relating to field study approvals (i.e., approving body and any reference numbers): Ministerio de Ambiente, Agua y Transición Ecológica (MAATE). No 019-2018-IC-FAU-DNB/MAE No MAE-DNB-CM-2018-0106.

### Data Availability

The following information was supplied regarding data availability: The data is available in the Supplemental Files. The raw sequence data are available at GenBank: PQ461843, PQ461838, PQ462301

PQ461844, PQ461839, PQ462300, PQ461845, PQ461840, PQ462298, PQ461837, PQ462302

### New Species Registration

The following information was supplied regarding the registration of a newly described species: Publication LSID: urn:lsid:zoobank.org:pub:DF37A1F3-4F65-4B1E-8C16-1852E79E4A4F *Epicrionops jameskenti* LSID: urn:lsid:zoobank.org:act:858A2D4A-11C8-4B2A-B771-78BE3112C2E5.
