# Supplementary material for: A new species of Epicrionops (Amphibia: Gymnophiona: Rhinatrematidae) from Ecuador, with integrative morphological and phylogenetic insights into Ecuadorian Epicrionops Boulenger, 1883 and Amazops Wilkinson, Reynolds & Jacobs, 2021
Source: PeerJ. 2026 Nov 10;14:e21786. doi: 10.7717/peerj.21786 (PMC13762366; doi:10.7717/peerj.21786)
Supplement: Supplementary file 1 [file peerj-14-21786-s001.docx]

**Appendix I.** Examined specimens and locality coordinates.

| **Museum number** | ***Species*** | **Type** | **Province, Country** | **Latitude** | **Longitude** | **Elevation** | **Locality** |
| --- | --- | --- | --- | --- | --- | --- | --- |
| QCAZ 42406 | *Amazops amazops* | Examined specimen | Napo, Ecuador | -0.134 | -77.4671 | 1312 | Bosque Protector Tigre, Bosque Protector La Cascada y Reserva de Biosfera Sumaco |
| QCAZ 42407 | *Amazops amazops* | Examined specimen | Napo, Ecuador | -0.134 | -77.4671 | 1270 | Bosque Protector Tigre, Bosque Protector La Cascada y Reserva de Biosfera Sumaco, Santa Rosa |
| QCAZ 42405 | *Amazops amazops* | Examined specimen | Napo, Ecuador | -0.134 | -77.4671 | 1270 | Bosque Protector Tigre, Bosque Protector La Cascada y Reserva de Biosfera Sumaco, Santa Rosa |
| MEPN 07801 | *Amazops amazops* | Examined specimen | Napo, Ecuador | -0.6684 | -77.7971 | 1706 | Cotundo, Archidona |
| QCAZ 03210 | *Amazops amazops* | Examined specimen | Napo, Ecuador | -0.532 | -77.882 | 1860 | Junto a Río Cosanga, Cosanga-Baeza |
| ZSFQ 00814 | *Amazops amazops* | Examined specimen | Napo, Ecuador | -0.615 | -77.5894 | 1755 | Parque Nacional Sumaco, Quijos |
| ZSFQ 00815 | *Amazops amazops* | Examined specimen | Napo, Ecuador | -0.615 | -77.5894 | 1755 | Parque Nacional Sumaco, Quijos |
| QCAZ 74837 | *Amazops amazops* | Examined specimen | Napo, Ecuador | -0.6268 | -77.8424 | 2240 | Reserva Ecológica Antisana, Sendero Jumandi |
| QCAZ 56870 | *Amazops amazops* | Examined specimen | Napo, Ecuador | -0.6714 | -77.5987 | 1520 | Wildsumaco Wildlife Sactuary |
| QCAZ 70228 | *Amazops amazops* | Examined specimen | Pastaza, Ecuador | -1.3993 | -78.0593 | 1210 | Bloque 28, 8.5 km NE de Mera |
| DHMECN 15867 | *Amazops amazops* | Examined specimen | Pastaza, Ecuador | -1.4014 | -78.0553 | 1193 | Mera |
| DHMECN 14152 | *Amazops amazops* | Examined specimen | Pastaza, Ecuador | -1.4061 | -78.0492 | 1100 | Reserva Sumak Kawsay in situ |
| DHMECN 19951 | *Amazops amazops* | Examined specimen | Tungurahua, Ecuador | -1.3922 | -78.2938 | 2073 | Cerro Mayordomo, Río Verde |
| DHMECN 19465 | *E. bicolor-marmoratus* complex | Examined specimen | Carchi, Ecuador | 0.8632 | -78.2033 | 2400 | Reserva Drácula, Chical |
| MZUTI 05543 | *E. bicolor-marmoratus* complex | Examined specimen | Cotopaxi, Ecuador | -0.8365 | -79.2083 | 495 | Bosque Privado Jardín de los Sueños, La Maná |
| QCAZ 01965 | *E. bicolor-marmoratus* complex | Examined specimen | Cotopaxi, Ecuador | -0.4333 | -78.9667 | 1590 | Las Pampas y alrededores |
| QCAZ 01963 | *E. bicolor-marmoratus* complex | Examined specimen | Cotopaxi, Ecuador | -0.4333 | -78.9667 | 1590 | Las Pampas y alrededores |
| QCAZ 01977 | *E. bicolor-marmoratus* complex | Examined specimen | Cotopaxi, Ecuador | -0.4333 | -78.9667 | 1590 | Las Pampas y alrededores |
| QCAZ 01964 | *E. bicolor-marmoratus* complex | Examined specimen | Cotopaxi, Ecuador | -0.4333 | -78.9667 | 1590 | Las Pampas y alrededores |
| QCAZ 01969 | *E. bicolor-marmoratus* complex | Examined specimen | Cotopaxi, Ecuador | -0.4333 | -78.9667 | 1590 | Las Pampas y alrededores |
| QCAZ 01968 | *E. bicolor-marmoratus* complex | Examined specimen | Cotopaxi, Ecuador | -0.4333 | -78.9667 | 1590 | Las Pampas y alrededores |
| QCAZ 01979 | *E. bicolor-marmoratus* complex | Examined specimen | Cotopaxi, Ecuador | -0.4333 | -78.9667 | 1590 | Las Pampas y alrededores |
| QCAZ 01978 | *E. bicolor-marmoratus* complex | Examined specimen | Cotopaxi, Ecuador | -0.4333 | -78.9667 | 1590 | Las Pampas y alrededores |
| QCAZ 01970 | *E. bicolor-marmoratus* complex | Examined specimen | Cotopaxi, Ecuador | -0.4333 | -78.9667 | 1590 | Las Pampas y alrededores |
| QCAZ 01980 | *E. bicolor-marmoratus* complex | Examined specimen | Cotopaxi, Ecuador | -0.4333 | -78.9667 | 1590 | Las Pampas y alrededores |
| QCAZ 02690 | *E. bicolor-marmoratus* complex | Examined specimen | Cotopaxi, Ecuador | -0.41 | -78.967 | 1740 | Recinto Galápagos |
| QCAZ 08723 | *E. bicolor-marmoratus* complex | Examined specimen | Imbabura, Ecuador | 0.2754 | -78.6603 | 1320 | La Mina, Junín |
| QCAZ 20138 | *E. bicolor-marmoratus* complex | Examined specimen | Santo Domingo de los Tsáchilas, Ecuador | -0.4333 | -79 | 2100 | Bosque Integral Otonga |
| QCAZ 11861 | *E. bicolor-marmoratus* complex | Examined specimen | Santo Domingo de los Tsáchilas, Ecuador | -0.4167 | -79 | 1860 | Bosque Integral Otonga |
| QCAZ 15930 | *E. bicolor-marmoratus* complex | Examined specimen | Santo Domingo de los Tsáchilas, Ecuador | -0.229 | -78.806 | 2000 | Estación Científica Guajalito, Las Palmeras |
| QCAZ 01966 | *E. bicolor-marmoratus* complex | Examined specimen | Santo Domingo de los Tsáchilas, Ecuador | -0.229 | -78.806 | 2220 | Las Palmeras, cerca de Chiriboga |
| QCAZ 01961 | *E. bicolor-marmoratus* complex | Examined specimen | Santo Domingo de los Tsáchilas, Ecuador | -0.244 | -78.794 | 1940 | Palmeras |
| ZSFQ 02818 | *E. bicolor-marmoratus* complex | Examined specimen | Santo Domingo de los Tsáchilas, Ecuador | -0.211 | -78.8043 | 2400 | Reserva Río Guajalito |
| ZSFQ 02817 | *E. bicolor-marmoratus* complex | Examined specimen | Santo Domingo de los Tsáchilas, Ecuador | -0.211 | -78.8043 | 2400 | Reserva Río Guajalito |
| ZSFQ 02819 | *E. bicolor-marmoratus* complex | Examined specimen | Santo Domingo de los Tsáchilas, Ecuador | -0.211 | -78.8043 | 1880 | Reserva Río Guajalito |
| QCAZ 03245 | *E. bicolor-marmoratus* complex | Examined specimen | Pichincha, Ecuador | 0.0059 | -78.6746 | 1660 | Tandayapa |
| DHMECN 09311 | *E. bicolor-marmoratus* complex | Examined specimen | Bolivar, Ecuador | -1.7644 | -79.192 | 751 | Guanujo, Guaranda |
| DHMECN 10828 | *E. bicolor-marmoratus* complex | Examined specimen | El Oro, Ecuador | -3.6909 | -79.4637 | 2354 | Hacienda Chepel |
| DHMECN 01523 | *E. bicolor-marmoratus* complex | Examined specimen | El Oro, Ecuador | -3.5667 | -79.6667 | 1650 | Paccha, Atahualpa |
| MEPN 05198 | *E. bicolor-marmoratus* complex | Examined specimen | El Oro, Ecuador | -3.6794 | -79.4553 | 1699 | San Antonio de Güizagüiña, Zaruma |
| MUTPL 03997 | *E. jameskenti* | Examined specimen | Morona Santiago, Ecuador | -3.1962 | -78.2879 | 1030 | Centro Shuar Yawi |
| MUTPL 03998 | *E. jameskenti* | Examined specimen | Morona Santiago, Ecuador | -3.1962 | -78.2879 | 1030 | Centro Shuar Yawi |
| ZSFQ 04136 | *E. jameskenti* | Examined specimen | Morona Santiago, Ecuador | -2.7724 | -78.4776 | 1490 | Parque Nacional Río Negro-Sopladora, El Rosario |
| ZSFQ 04137 | *E. jameskenti* | Examined specimen | Morona Santiago, Ecuador | -2.7724 | -78.4776 | 1490 | Parque Nacional Río Negro-Sopladora, El Rosario |
| MZUA 02161 | *E. jameskenti* | Examined specimen | Morona Santiago, Ecuador | -2.7724 | -78.4776 | 1490 | Parque Nacional Río Negro-Sopladora, El Rosario |
| ZSFQ 04138 | *E. jameskenti* | Examined specimen | Morona Santiago, Ecuador | -2.7724 | -78.4776 | 1490 | Parque Nacional Río Negro-Sopladora, El Rosario |
| MZUA 02854 | *E. jameskenti* | Examined specimen | Morona Santiago, Ecuador | -3.1521 | -78.561 | 1600 | San Juan Bosco, Dave Goucher farm |
| DHMECN 04600 | *E. jameskenti* | Examined specimen | Zamora Chinchipe, Ecuador | -4.4655 | -78.794 | 1360 | Reserva Biológica Tapichalaca, Quebrada Honda |
| QCAZ 79921 | *E. jameskenti* | Examined specimen | Zamora Chinchipe, Ecuador | -3.9101 | -78.4892 | 1850 | Los Encuentros, Concesión Lundingold (Emperador-Río Blanco) |
| QCAZ 76506 | *E. petersi* | Examined specimen | Zamora Chinchipe, Ecuador | -3.5842 | -78.418 | 1640 | Microcuenca del Río Wawaime, Concesión ECSA |
