# Supplementary material for: A new species of Epicrionops (Amphibia: Gymnophiona: Rhinatrematidae) from Ecuador, with integrative morphological and phylogenetic insights into Ecuadorian Epicrionops Boulenger, 1883 and Amazops Wilkinson, Reynolds & Jacobs, 2021
Source: PeerJ. 2026 Nov 10;14:e21786. doi: 10.7717/peerj.21786 (PMC13762366; doi:10.7717/peerj.21786)
Supplement: Supplementary file 2 [file peerj-14-21786-s002.docx]

**Appendix II.** Counts and measurements of *Epicrionops* and *Amazops*. All measurements in mm. Acronyms from Wei et al. (2025): Total length (**TOL**), Snout-vent length (**SVL**), Trunk length (**TRL**), Tail length (**TL**), Body width 1 (**BW1**), Body width 2 (**BW2**), Body width 3 (**BW3**), Body width 4 (**BW4**), Body height (**BH**), Head length (**HL**), Head width 1 (**HW1**), Head width 2 (**HW2**), Head height 1 (**HH1**), Head height 2 (**HH2**),	Snout to 1st collar groove (**STFG**), Snout to 2nd collar groove (**STSG**), Snout to 3rd collar groove (**STTG**), Interorbital distance (**IOD**), Eye diameter (**ED**), Eye-nostril distance (**EN**), Eye-jaw distance (**EJ**), Snout-eye distance (**SE**), Tentacle-lip distance (**TLP**), Internostril distance (**IND**), Nostril-snout distance (**NS**), Nostril-jaw distance (**NJ**), Naris diameter (**ND**), Naris to margin of lip distance (**NLP**), Naris to nuchal groove distance (**NFNG**), Snout-jaw distance (**SJ**), Lower jaw to nuchal groove (**LN**), Lower jaw length (**LJL**), Jaw to nuchal groove distance (**JNG**), Tail width at posterior vent (**TAW**), Vent annuli (**VA**), Anal disc diameter (**ADD**), 1st collar length (**C1**), 2nd collar length (**C2**), Circumference at midbody (**CM**), Total annuli 1 (**TA1**), Total annuli 2 (**TA2**), Post-vent annuli (**PVA**).

| **Museum number** | **Locality** | **Species** | **Sex** | **Development st.** | **TOL** | **SVL** | **TRL** | **TL** | **BW1** | **BW2** | **BW3** | **BW4** | **BH** | **HL** | **HW1** |
| --- | --- | --- | --- | --- | --- | --- | --- | --- | --- | --- | --- | --- | --- | --- | --- |
| QCAZ-42406 | Napo | *A. amazops* | Larvae | Stage 37 | 140 | 127 | 120 | 8.0 | 5.8 | 5.7 | 4.3 | 5.9 | 4.2 | 8.4 | 5.3 |
| ZSFQ-00814 | Napo | *A. amazops* | Larvae | Stage 37 | 181 | 174 | 159 | 8.5 | 7.1 | 9.2 | 5.7 | 7.2 | 7.0 | 9.5 | 7.0 |
| ZSFQ-00815 | Napo | *A. amazops* | Larvae | Stage 37 | 192 | 167 | 148 | 8.0 | 7.6 | 8.2 | 5.3 | 7.6 | 7.3 | 9.0 | 7.1 |
| DHMECN-19951 | Tungurahua | *A. amazops* | Larvae | Stage 37 | 129 | 125 | 117 | 5.3 | 6.6 | 7.3 | 5.0 | 7.0 | 5.5 | 8.7 | 5.6 |
| QCAZ-56870 | Napo | *A. amazops* | Male | Fully metamorphosed | 184 | 167 | 159 | 8.3 | 6.6 | 9.1 | 5.9 | 7.1 | 4.7 | 9.8 | 6.6 |
| QCAZ-70228 | Pastaza | *A. amazops* | Male | Fully metamorphosed | 193 | 189 | 170 | 7.3 | 6.7 | 8.5 | 5.6 | 7.1 | 7.5 | 8.0 | 6.6 |
| QCAZ-42407 | Napo | *A. amazops* | Male | Adult | 202 | 192 | 183 | 9.2 | 7.6 | 9.7 | 6.1 | 7.9 | 6.7 | 10.6 | 7.6 |
| MEPN-07801 | Napo | *A. amazops* | Male | Fully metamorphosed | 225 | 212 | 204 | 7.8 | 7.5 | 9.2 | 4.9 | 8.4 | 8.9 | 10.6 | 7.0 |
| DHMECN-15867 | Pastaza | *A. amazops* | Male | Fully metamorphosed | 232 | 211 | 193 | 5.6 | 7.5 | 8.9 | 5.5 | 8.1 | 3.9 | 10.5 | 7.4 |
| QCAZ-42405 | Napo | *A. amazops* | Male | Adult | 249 | 232 | 218 | 10.8 | 11.2 | 11.5 | 7.5 | 11.3 | 8.9 | 13.5 | 9.9 |
| DHMECN-14152 | Pastaza | *A. amazops* | Female | Fully metamorphosed | 234 | 206 | 193 | 6.8 | 5.8 | 10.0 | 6.8 | 6.0 | 6.7 | 8.7 | 5.8 |
| QCAZ-03210 | Napo | *A. amazops* | Female | Fully metamorphosed | 255 | 245 | 231 | 8.1 | 8.5 | 9.6 | 5.1 |  | 6.0 | 12.1 | 8.0 |
| QCAZ-74837 | Napo | *A. amazops* | Female | Adult | 295 | 287 | 264 | 10.1 | 9.7 | 12.7 | 8.1 | 9.8 | 11.2 | 14.1 | 8.9 |
| QCAZ-01965 | Cotopaxi | *E. bicolor-marmoratus* complex | Larvae | Stage 37 | 105 | 92 | 88 | 5.8 |  |  |  |  |  |  |  |
| QCAZ-01963 | Cotopaxi | *E. bicolor-marmoratus* complex | Larvae | Stage 37 | 143 | 133 | 125 | 9.5 | 6.8 | 8.5 | 5.3 | 6.6 | 5.4 | 7.8 | 6.1 |
| QCAZ-01977 | Cotopaxi | *E. bicolor-marmoratus* complex | Larvae | Stage 39 | 167 | 159 | 143 | 9.4 | 7.5 | 8.8 | 6.7 | 6.7 | 7.4 | 9.6 | 6.4 |
| QCAZ-01964 | Cotopaxi | *E. bicolor-marmoratus* complex | Larvae | Stage 37 | 188 | 165 | 149 | 10.3 | 6.8 | 8.0 | 5.0 | 6.6 | 7.5 | 9.2 | 6.5 |
| QCAZ-20138 | Santo de los Tsáchilas | *E. bicolor-marmoratus* complex | Male | Fully metamorphosed | 214 | 180 | 177 | 10.6 | 7.8 | 9.8 | 7.2 | 7.9 | 6.9 | 10.1 | 7.0 |
| QCAZ-02690 | Cotopaxi | *E. bicolor-marmoratus* complex | Male | Adult | 244 | 233 | 225 | 14.6 | 8.2 | 13.3 | 9.5 | 9.5 | 6.4 | 13.5 | 8.0 |
| QCAZ-01969 | Cotopaxi | *E. bicolor-marmoratus* complex | Male | Adult | 245 | 200 | 190 | 12.1 | 10.0 | 13.8 | 11.3 | 10.4 | 12.6 | 11.8 | 9.2 |
| QCAZ-01968 | Cotopaxi | *E. bicolor-marmoratus* complex | Male | Adult | 266 | 194 | 240 | 14.8 | 10.9 | 16.1 | 11.4 | 11.5 | 12.3 | 13.3 | 8.9 |
| QCAZ-01979 | Cotopaxi | *E. bicolor-marmoratus* complex | Male | Adult | 289 | 213 | 200 | 15.0 | 9.9 | 16.3 | 12.5 | 10.9 | 13.2 | 12.7 | 9.2 |
| QCAZ-01978 | Cotopaxi | *E. bicolor-marmoratus* complex | Male | Adult | 292 | 222 | 215 | 15.0 | 9.7 | 15.5 | 12.0 | 10.6 | 13.6 | 12.5 | 8.6 |
| ZSFQ-02818 | Santo de los Tsáchilas | *E. bicolor-marmoratus* complex | Male | Adult | 324 | 217 | 301 | 14.4 | 10.4 | 16.2 | 9.9 | 10.8 | 11.6 | 14.2 | 10.2 |
| QCAZ-01961 | Santo de los Tsáchilas | *E. bicolor-marmoratus* complex | Male | Adult | 327 | 255 | 244 | 13.3 | 10.2 | 15.1 | 10.9 | 10.4 | 12.0 | 14.2 | 10.5 |
| QCAZ-01970 | Cotopaxi | *E. bicolor-marmoratus* complex | Female | Adult | 261 | 190 | 180 | 13.0 | 9.5 | 16.4 | 11.9 | 9.9 | 13.3 | 11.5 | 8.1 |
| MZUTI-05543 | Cotopaxi | *E. bicolor-marmoratus* complex | Female | Fully metamorphosed | 270 | 243 | 229 | 13.0 | 7.5 | 11.6 | 7.5 | 7.5 | 9.3 | 11.0 | 7.4 |
| QCAZ-01966 | Santo de los Tsáchilas | *E. bicolor-marmoratus* complex | Female | Adult | 286 | 272 | 252 | 15.1 | 9.3 | 14.1 | 8.8 | 11.0 | 12.5 | 13.2 | 9.2 |
| QCAZ-01980 | Cotopaxi | *E. bicolor-marmoratus* complex | Female | Adult | 305 | 240 | 213 | 17.4 | 11.2 | 16.5 | 12.4 | 10.4 | 13.8 | 13.9 | 9.9 |
| ZSFQ-2817 | Santo de los Tsáchilas | *E. bicolor-marmoratus* complex | Female | Adult | 358 | 277 | 263 | 14.1 | 12.4 | 15.5 | 12.7 | 13.6 | 13.7 | 14.7 | 11.1 |
| DHMECN-19465 | Carchi | *E. bicolor-marmoratus* complex | Male | Adult | 270 | 293 | 285 | 14.2 | 10.3 | 16.0 | 12.0 | 10.6 | 13.7 | 14.1 | 9.9 |
| QCAZ-11861 | Santo de los Tsáchilas | *E. bicolor-marmoratus* complex | Female | Adult | 297 | 244 | 225 | 14.3 | 9.1 | 14.0 | 9.8 | 9.0 | 10.3 | 12.6 | 8.7 |
| QCAZ-15930 | Santo de los Tsáchilas | *E. bicolor-marmoratus* complex | ND | Fully metamorphosed | 243 |  |  |  |  |  |  |  |  |  |  |
| ZSFQ-02819 | Santo de los Tsáchilas | *E. bicolor-marmoratus* complex | ND | Adult | 275 |  |  |  |  |  |  |  |  |  |  |
| QCAZ-08723 | Imbabura | *E. bicolor-marmoratus* complex | Male | Adult | 277 | 266 | 256 | 16.4 | 11.5 | 13.1 | 10.8 | 12.0 | 9.9 | 13.6 | 11.7 |
| QCAZ-03245 | Pichincha | *E. bicolor-marmoratus* complex | Female | Adult | 320 | 315 | 292 | 5.3 | 10.8 | 16.4 | 10.7 | 11.7 | 15.3 | 14.9 | 11.0 |
| DHMECN-09311 | Bolivar | *E. bicolor-marmoratus* complex | Female | Adult | 389 | 335 | 320 | 6.8 | 15.2 | 18.6 | 13.5 | 15.5 | 18.8 | 19.2 | 14.6 |
| DHMECN-01523 | El Oro | *E. bicolor-marmoratus* complex | Larvae | Stage 37 | 135 | 125 | 114 | 10.1 | 6.4 | 7.8 | 5.5 | 6.6 | 5.9 | 7.7 | 5.6 |
| DHMECN-10828 | El Oro | *E. bicolor-marmoratus* complex | Male | Adult | 335 | 284 | 275 | 4.7 | 11.9 | 16.6 | 9.2 | 14.1 | 10.2 | 16.3 | 11.9 |
| MEPN-05198 | El Oro | *E. bicolor-marmoratus* complex | Female | Fully metamorphosed | 214 | 194 | 181 | 3.3 | 8.7 | 10.3 | 7.1 | 9.5 | 7.5 | 11.7 | 8.1 |
| QCAZ-76506 | Zamora Chinchipe | *E. petersi* | Male | Adult | 300 | 264 | 258 | 4.5 | 11.2 | 14.9 | 10.5 | 11.4 | 11.7 | 12.2 | 10.8 |
| QCAZ-79921 | Zamora Chinchipe | *E. jameskenti* | Female | Adult | 317 | 287 | 276 | 6.5 | 10.6 | 21.9 | 12.0 | 11.9 | 14.8 | 13.2 | 10.2 |
| MUTPL-03997 | Morona Santiago | *E. jameskenti* | Larvae | Stage 38 | 154 | 145 | 133 | 10.2 | 6.7 | 8.7 | 5.8 | 6.3 | 6.3 | 8.7 | 6.6 |
| ZSFQ-04136 | Morona Santiago | *E. jameskenti* | Larvae | Stage 39 | 183 | 154 | 136 | 10.3 | 7.6 | 8.8 | 6.1 | 7.6 | 6.3 | 9.2 | 6.6 |
| ZSFQ-04137 | Morona Santiago | *E. jameskenti* | Larvae | Stage 39 | 184 | 140 | 130 | 10.2 | 8.0 | 8.5 | 6.7 | 8.0 | 6.4 | 9.6 | 7.1 |
| MZUA-02161 | Morona Santiago | *E. jameskenti* | Larvae | Stage 37 | 213 | 170 | 153 | 11.7 | 9.3 | 10.0 | 7.6 | 8.8 | 8.5 | 10.9 | 8.7 |
| DHMECN-04600 | Zamora Chinchipe | *E. jameskenti* | Male | Fully metamorphosed | 174 | 157 | 140 | 9.1 | 6.7 | 7.6 | 5.8 | 6.9 | 6.7 | 10.1 | 6.1 |
| MUTPL-03998 | Morona Santiago | *E. jameskenti* | Male | Fully metamorphosed | 210 | 182 | 169 | 12.8 | 8.0 | 10.1 | 7.7 | 8.4 | 7.1 | 10.2 | 7.4 |
| ZSFQ-04138 | Morona Santiago | *E. jameskenti* | Male | Fully metamorphosed | 211 | 206 | 173 | 10.8 | 8.5 | 10.8 | 7.6 | 9.2 | 8.9 | 10.8 | 7.5 |
| MZUA-02854 | Morona Santiago | *E. jameskenti* | Female | Fully metamorphosed | 215 | 193 | 181 | 11.7 | 8.1 | 10.2 | 8.7 | 8.8 | 7.7 | 10.8 | 7.7 |
| **Museum number** | **Locality** | **Species** | **Sex** | **Development st.** | **HW2** | **HH1** | **HH2** | **STFG** | **STSG** | **STTG** | **IOD** | **ED** | **EN** | **EJ** | **SE** |
| QCAZ-42406 | Napo | *A. amazops* | Larvae | Stage 37 | 6.2 | 4.4 | 4.2 |  |  |  | 3.2 |  | 0.5 | 3.1 | 2.5 |
| ZSFQ-00814 | Napo | *A. amazops* | Larvae | Stage 37 | 7.1 | 6.0 | 5.1 | 10.4 | 11.8 | 15.2 | 4.3 | 0.4 | 2.8 | 3.4 | 3.6 |
| ZSFQ-00815 | Napo | *A. amazops* | Larvae | Stage 37 | 7.3 | 6.6 | 6.2 | 10.9 | 12.1 | 14.5 | 4.4 | 0.5 | 2.9 | 3.5 | 3.6 |
| DHMECN-19951 | Tungurahua | *A. amazops* | Larvae | Stage 37 | 6.9 | 5.3 | 4.6 | 8.4 | 8.7 | 11.8 | 3.6 |  | 2.4 | 2.8 | 2.9 |
| QCAZ-56870 | Napo | *A. amazops* | Male | Fully metamorphosed | 6.8 | 5.1 | 4.8 | 10.2 | 12.0 | 14.8 | 3.9 | 0.5 | 3.2 | 4.6 | 4.2 |
| QCAZ-70228 | Pastaza | *A. amazops* | Male | Fully metamorphosed | 6.8 | 5.0 | 4.5 | 9.8 | 11.3 | 16.2 | 4.1 | 0.4 | 3.7 | 4.5 | 4.4 |
| QCAZ-42407 | Napo | *A. amazops* | Male | Adult | 8.0 | 4.9 | 4.6 | 11.1 | 13.4 | 18.4 | 4.7 | 0.4 | 3.5 | 5.5 | 4.7 |
| MEPN-07801 | Napo | *A. amazops* | Male | Fully metamorphosed | 7.6 | 5.8 | 5.4 | 11.1 | 12.6 | 17.9 | 4.0 | 0.5 | 3.9 | 3.7 | 4.7 |
| DHMECN-15867 | Pastaza | *A. amazops* | Male | Fully metamorphosed | 7.7 | 3.7 | 3.9 | 10.4 | 11.5 | 14.9 | 4.3 | 0.5 | 3.4 | 6.1 | 4.3 |
| QCAZ-42405 | Napo | *A. amazops* | Male | Adult | 11.1 | 7.8 | 7.1 | 14.1 | 16.2 | 22.4 | 5.7 | 0.6 | 3.9 | 6.1 | 5.0 |
| DHMECN-14152 | Pastaza | *A. amazops* | Female | Fully metamorphosed | 6.1 | 4.2 | 3.5 | 9.5 | 10.9 | 14.7 | 3.6 | 0.4 | 3.5 | 3.8 | 4.1 |
| QCAZ-03210 | Napo | *A. amazops* | Female | Fully metamorphosed |  | 5.1 | 4.9 | 11.9 | 13.7 | 19.8 | 4.5 | 0.6 | 3.8 | 5.8 | 4.6 |
| QCAZ-74837 | Napo | *A. amazops* | Female | Adult | 9.1 | 7.3 | 6.6 | 13.9 | 17.4 | 23.9 | 5.5 | 0.7 | 4.5 | 5.3 | 5.2 |
| QCAZ-01965 | Cotopaxi | *E. bicolor-marmoratus* complex | Larvae | Stage 37 |  |  |  |  |  |  |  |  |  |  |  |
| QCAZ-01963 | Cotopaxi | *E. bicolor-marmoratus* complex | Larvae | Stage 37 | 6.7 | 5.3 | 4.0 |  |  |  |  |  |  |  |  |
| QCAZ-01977 | Cotopaxi | *E. bicolor-marmoratus* complex | Larvae | Stage 39 | 8.0 | 5.1 | 4.2 | 10.6 | 13.6 | 15.6 | 4.2 | 0.4 | 3.3 | 3.5 | 4.1 |
| QCAZ-01964 | Cotopaxi | *E. bicolor-marmoratus* complex | Larvae | Stage 37 | 6.8 | 5.9 | 3.9 | 9.8 | 11.3 | 13.6 | 3.9 | 0.5 | 3.1 | 3.9 | 3.5 |
| QCAZ-20138 | Santo de los Tsáchilas | *E. bicolor-marmoratus* complex | Male | Fully metamorphosed | 8.0 | 6.1 | 5.7 | 10.7 | 12.2 | 15.8 | 4.3 | 0.6 | 3.8 | 3.1 | 4.4 |
| QCAZ-02690 | Cotopaxi | *E. bicolor-marmoratus* complex | Male | Adult | 8.0 | 5.9 | 5.3 | 13.0 | 13.6 | 18.9 | 4.3 | 0.5 |  | 6.3 | 5.0 |
| QCAZ-01969 | Cotopaxi | *E. bicolor-marmoratus* complex | Male | Adult | 9.6 | 6.7 | 5.8 | 11.7 | 14.3 | 19.0 | 4.7 | 0.5 | 3.8 | 4.6 | 3.7 |
| QCAZ-01968 | Cotopaxi | *E. bicolor-marmoratus* complex | Male | Adult | 10.0 | 6.0 | 5.7 | 13.4 | 14.2 | 19.6 | 5.0 | 0.7 | 4.5 | 4.9 | 5.2 |
| QCAZ-01979 | Cotopaxi | *E. bicolor-marmoratus* complex | Male | Adult | 10.0 | 7.8 | 7.1 | 12.6 | 15.9 | 20.5 | 4.8 | 0.7 | 4.3 | 5.6 | 5.2 |
| QCAZ-01978 | Cotopaxi | *E. bicolor-marmoratus* complex | Male | Adult | 10.2 | 6.6 | 6.0 | 13.0 | 14.9 | 19.9 | 4.8 | 0.4 | 4.3 | 5.0 | 5.3 |
| ZSFQ-02818 | Santo de los Tsáchilas | *E. bicolor-marmoratus* complex | Male | Adult | 10.5 | 7.1 | 7.1 | 15.5 | 17.5 | 25.4 | 5.6 | 0.6 | 5.0 | 7.0 | 6.4 |
| QCAZ-01961 | Santo de los Tsáchilas | *E. bicolor-marmoratus* complex | Male | Adult | 11.2 | 8.5 | 7.8 | 14.8 | 17.6 | 23.8 | 5.8 | 0.7 | 4.9 | 7.5 | 5.7 |
| QCAZ-01970 | Cotopaxi | *E. bicolor-marmoratus* complex | Female | Adult | 8.7 | 5.5 | 5.3 | 11.4 | 13.3 | 19.3 | 4.4 | 0.5 | 3.9 | 5.0 | 4.7 |
| MZUTI-05543 | Cotopaxi | *E. bicolor-marmoratus* complex | Female | Fully metamorphosed | 8.0 | 6.1 | 5.6 | 10.2 | 12.5 | 17.8 | 4.2 | 0.4 | 3.6 | 4.9 | 4.4 |
| QCAZ-01966 | Santo de los Tsáchilas | *E. bicolor-marmoratus* complex | Female | Adult | 10.0 | 8.0 | 6.2 | 13.2 | 15.9 | 20.2 | 5.3 | 0.5 | 4.5 | 5.9 | 5.8 |
| QCAZ-01980 | Cotopaxi | *E. bicolor-marmoratus* complex | Female | Adult | 10.6 | 7.7 | 7.0 | 13.8 | 17.3 | 22.6 | 4.7 | 0.6 | 4.7 | 5.6 | 5.4 |
| ZSFQ-2817 | Santo de los Tsáchilas | *E. bicolor-marmoratus* complex | Female | Adult | 12.0 | 10.1 | 10.1 | 14.7 | 18.9 | 25.4 | 5.9 | 0.6 | 5.3 | 6.9 | 6.5 |
| DHMECN-19465 | Carchi | *E. bicolor-marmoratus* complex | Male | Adult | 10.5 | 8.2 | 7.7 | 14.4 | 17.9 | 22.1 | 5.6 | 0.6 | 4.8 | 6.5 | 5.5 |
| QCAZ-11861 | Santo de los Tsáchilas | *E. bicolor-marmoratus* complex | Female | Adult | 9.3 | 6.6 | 5.6 | 12.7 | 15.1 | 21.2 | 4.9 | 0.7 | 4.5 | 5.8 | 5.2 |
| QCAZ-15930 | Santo de los Tsáchilas | *E. bicolor-marmoratus* complex | ND | Fully metamorphosed |  |  |  |  |  |  |  |  |  |  |  |
| ZSFQ-02819 | Santo de los Tsáchilas | *E. bicolor-marmoratus* complex | ND | Adult |  |  |  |  |  |  |  |  |  |  |  |
| QCAZ-08723 | Imbabura | *E. bicolor-marmoratus* complex | Male | Adult | 9.8 | 6.7 | 5.7 | 13.9 | 14.9 | 23.2 | 4.7 | 0.5 | 4.0 | 8.7 | 5.2 |
| QCAZ-03245 | Pichincha | *E. bicolor-marmoratus* complex | Female | Adult | 11.8 | 7.5 | 6.9 | 14.7 | 17.5 | 24.3 | 6.4 | 0.7 | 5.4 | 7.6 | 6.4 |
| DHMECN-09311 | Bolivar | *E. bicolor-marmoratus* complex | Female | Adult | 15.4 | 13.3 | 11.9 | 20.8 | 23.0 | 31.6 | 7.6 | 0.7 | 6.1 | 9.3 | 7.8 |
| DHMECN-01523 | El Oro | *E. bicolor-marmoratus* complex | Larvae | Stage 37 | 6.4 | 5.4 | 5.3 |  |  |  |  |  |  |  |  |
| DHMECN-10828 | El Oro | *E. bicolor-marmoratus* complex | Male | Adult | 12.4 | 10.0 | 7.9 | 17.3 | 18.8 | 25.7 | 6.6 | 0.7 | 5.2 | 9.3 | 6.5 |
| MEPN-05198 | El Oro | *E. bicolor-marmoratus* complex | Female | Fully metamorphosed | 8.4 | 5.7 | 5.0 | 12.4 | 13.9 | 18.4 | 5.2 | 0.6 | 4.4 | 4.7 | 4.9 |
| QCAZ-76506 | Zamora Chinchipe | *E. petersi* | Male | Adult | 11.2 | 8.3 | 7.5 | 12.7 | 14.7 | 20.2 | 5.3 | 0.5 | 4.1 | 6.9 | 5.3 |
| QCAZ-79921 | Zamora Chinchipe | *E. jameskenti* | Female | Adult | 10.4 | 8.4 | 6.6 | 13.5 | 15.6 | 21.5 | 5.3 | 0.6 | 4.6 | 6.2 | 5.5 |
| MUTPL-03997 | Morona Santiago | *E. jameskenti* | Larvae | Stage 38 | 7.0 | 5.5 | 4.3 | 9.4 | 9.8 | 12.8 | 3.5 | 0.5 | 2.7 | 3.5 | 3.3 |
| ZSFQ-04136 | Morona Santiago | *E. jameskenti* | Larvae | Stage 39 | 7.7 | 5.7 | 5.5 | 10.3 | 11.3 | 13.3 | 4.0 | 0.7 | 3.1 | 4.1 | 2.9 |
| ZSFQ-04137 | Morona Santiago | *E. jameskenti* | Larvae | Stage 39 | 7.6 | 5.7 | 5.3 | 10.3 | 11.4 | 13.8 | 4.2 | 0.6 | 3.2 | 4.6 | 3.9 |
| MZUA-02161 | Morona Santiago | *E. jameskenti* | Larvae | Stage 37 | 8.6 | 6.9 | 5.5 | 11.2 | 12.5 | 15.4 | 5.1 | 0.6 | 3.7 | 4.2 | 4.5 |
| DHMECN-04600 | Zamora Chinchipe | *E. jameskenti* | Male | Fully metamorphosed | 6.6 | 4.8 | 4.6 | 10.0 | 11.4 | 14.8 | 4.0 | 0.6 | 3.2 | 3.8 | 3.7 |
| MUTPL-03998 | Morona Santiago | *E. jameskenti* | Male | Fully metamorphosed | 7.7 | 5.7 | 5.8 | 10.4 | 11.9 | 16.9 | 4.6 | 0.6 | 4.3 | 4.4 | 4.9 |
| ZSFQ-04138 | Morona Santiago | *E. jameskenti* | Male | Fully metamorphosed | 8.9 | 5.7 | 5.8 | 11.0 | 13.2 | 17.2 | 4.5 | 0.6 | 3.4 | 4.1 | 4.5 |
| MZUA-02854 | Morona Santiago | *E. jameskenti* | Female | Fully metamorphosed | 8.1 | 5.9 | 5.0 | 10.8 | 12.4 | 16.1 | 4.7 | 0.5 | 3.6 | 4.8 | 4.6 |
| **Museum number** | **Locality** | **Species** | **Sex** | **Development st.** | **TLP** | **IND** | **NS** | **NJ** | **ND** | **NLP** | **NFNG** | **SJ** | **LN** | **LJL** | **JNG** |
| QCAZ-42406 | Napo | *A. amazops* | Larvae | Stage 37 |  |  |  |  |  |  |  |  |  |  |  |
| ZSFQ-00814 | Napo | *A. amazops* | Larvae | Stage 37 |  | 2.0 | 0.9 | 5.9 | 0.3 | 0.7 | 10.2 | 7.2 | 11.2 | 6.6 | 3.1 |
| ZSFQ-00815 | Napo | *A. amazops* | Larvae | Stage 37 |  | 2.1 | 1.0 | 6.4 | 0.3 | 0.5 | 10.0 | 7.6 | 8.6 | 6.4 | 1.5 |
| DHMECN-19951 | Tungurahua | *A. amazops* | Larvae | Stage 37 |  | 1.8 | 0.6 | 5.6 | 0.2 | 0.6 | 8.7 | 6.6 | 8.4 | 6.1 | 1.6 |
| QCAZ-56870 | Napo | *A. amazops* | Male | Fully metamorphosed | 0.9 | 1.7 | 1.0 | 7.7 | 0.2 | 0.7 | 9.7 | 8.8 | 10.2 | 8.4 | 2.2 |
| QCAZ-70228 | Pastaza | *A. amazops* | Male | Fully metamorphosed | 0.8 | 1.7 | 0.8 | 8.0 | 0.1 | 0.8 | 8.9 | 9.1 | 9.6 | 8.6 | 1.9 |
| QCAZ-42407 | Napo | *A. amazops* | Male | Adult | 0.8 | 1.8 | 0.9 | 8.9 | 0.2 | 0.8 | 10.7 | 10.2 | 12.7 | 9.5 | 3.6 |
| MEPN-07801 | Napo | *A. amazops* | Male | Fully metamorphosed | 0.9 | 1.4 | 0.7 | 7.6 | 0.2 | 0.8 | 9.3 | 9.0 | 10.2 | 8.4 | 1.5 |
| DHMECN-15867 | Pastaza | *A. amazops* | Male | Fully metamorphosed | 0.9 |  | 0.7 | 10.1 |  | 0.5 | 10.3 | 10.3 | 10.2 | 10.1 | 1.5 |
| QCAZ-42405 | Napo | *A. amazops* | Male | Adult | 1.0 | 2.3 | 0.9 | 10.9 | 0.4 | 1.1 | 12.9 | 12.0 | 15.8 | 11.8 | 4.7 |
| DHMECN-14152 | Pastaza | *A. amazops* | Female | Fully metamorphosed | 0.6 |  |  | 6.6 |  |  | 8.3 | 7.7 | 9.9 | 8.2 | 1.7 |
| QCAZ-03210 | Napo | *A. amazops* | Female | Fully metamorphosed | 0.8 | 1.4 | 0.7 | 10.2 | 0.2 | 0.8 | 11.8 | 11.0 | 12.7 | 10.4 | 1.8 |
| QCAZ-74837 | Napo | *A. amazops* | Female | Adult | 1.1 | 1.7 | 0.7 | 10.1 | 0.3 | 0.9 | 31.5 | 11.2 | 11.6 | 10.3 | 3.7 |
| QCAZ-01965 | Cotopaxi | *E. bicolor-marmoratus* complex | Larvae | Stage 37 |  |  |  |  |  |  |  |  |  |  |  |
| QCAZ-01963 | Cotopaxi | *E. bicolor-marmoratus* complex | Larvae | Stage 37 |  |  |  |  |  |  |  |  |  |  |  |
| QCAZ-01977 | Cotopaxi | *E. bicolor-marmoratus* complex | Larvae | Stage 39 |  | 2.1 | 0.7 | 7.3 | 0.3 | 0.6 | 10.1 | 8.3 | 9.9 | 8.1 | 1.6 |
| QCAZ-01964 | Cotopaxi | *E. bicolor-marmoratus* complex | Larvae | Stage 37 |  | 1.7 | 0.8 | 7.0 | 0.2 | 0.9 | 9.4 | 7.3 | 9.3 | 7.1 | 2.4 |
| QCAZ-20138 | Santo de los Tsáchilas | *E. bicolor-marmoratus* complex | Male | Fully metamorphosed | 0.9 | 1.4 | 0.6 | 7.6 | 0.1 | 0.6 | 9.9 | 8.2 | 10.6 | 7.4 | 2.8 |
| QCAZ-02690 | Cotopaxi | *E. bicolor-marmoratus* complex | Male | Adult | 0.9 |  |  |  |  |  |  | 10.4 | 12.5 | 9.7 | 2.4 |
| QCAZ-01969 | Cotopaxi | *E. bicolor-marmoratus* complex | Male | Adult | 1.1 | 1.3 | 0.7 | 9.0 | 0.2 | 0.6 | 11.0 | 9.6 | 11.4 | 9.4 | 11.1 |
| QCAZ-01968 | Cotopaxi | *E. bicolor-marmoratus* complex | Male | Adult | 1.1 | 1.3 | 0.5 | 9.2 | 0.3 | 0.7 | 12.9 | 10.0 | 13.0 | 9.7 | 1.9 |
| QCAZ-01979 | Cotopaxi | *E. bicolor-marmoratus* complex | Male | Adult | 1.4 | 1.5 | 0.6 | 10.2 | 0.3 | 1.1 | 12.2 | 11.2 | 13.2 | 10.4 | 2.1 |
| QCAZ-01978 | Cotopaxi | *E. bicolor-marmoratus* complex | Male | Adult | 1.3 | 1.2 | 0.6 | 9.4 | 0.4 | 0.9 | 12.4 | 10.6 | 12.5 | 10.4 | 2.4 |
| ZSFQ-02818 | Santo de los Tsáchilas | *E. bicolor-marmoratus* complex | Male | Adult | 1.3 | 2.0 | 1.0 | 12.6 | 0.3 | 0.9 | 14.7 | 14.1 | 15.8 | 13.6 | 3.2 |
| QCAZ-01961 | Santo de los Tsáchilas | *E. bicolor-marmoratus* complex | Male | Adult | 1.3 | 1.2 | 0.6 | 12.8 | 0.2 | 0.8 | 14.2 | 13.3 | 14.7 | 13.2 | 2.5 |
| QCAZ-01970 | Cotopaxi | *E. bicolor-marmoratus* complex | Female | Adult | 0.9 | 1.2 | 0.7 | 9.1 | 0.2 | 0.8 | 10.8 | 10.1 | 11.4 | 9.7 | 2.1 |
| MZUTI-05543 | Cotopaxi | *E. bicolor-marmoratus* complex | Female | Fully metamorphosed | 1.3 | 1.7 | 1.0 | 8.7 | 0.2 | 0.6 | 11.5 | 9.8 | 10.2 | 9.0 | 1.8 |
| QCAZ-01966 | Santo de los Tsáchilas | *E. bicolor-marmoratus* complex | Female | Adult | 1.3 | 1.4 | 0.7 | 10.9 | 0.1 | 0.9 | 13.0 | 12.0 | 13.7 | 11.4 | 2.6 |
| QCAZ-01980 | Cotopaxi | *E. bicolor-marmoratus* complex | Female | Adult | 1.3 | 1.6 | 0.9 | 13.8 | 0.2 | 1.1 | 13.3 | 14.7 | 14.9 | 12.3 | 3.0 |
| ZSFQ-2817 | Santo de los Tsáchilas | *E. bicolor-marmoratus* complex | Female | Adult | 1.6 | 1.7 | 0.8 | 12.4 | 0.3 | 0.9 | 14.5 | 13.1 | 16.3 | 12.3 | 3.9 |
| DHMECN-19465 | Carchi | *E. bicolor-marmoratus* complex | Male | Adult | 1.3 | 1.6 | 0.9 | 11.8 | 0.3 | 1.3 | 13.9 | 15.0 | 14.4 | 12.6 | 2.7 |
| QCAZ-11861 | Santo de los Tsáchilas | *E. bicolor-marmoratus* complex | Female | Adult | 1.0 | 1.6 | 0.8 | 10.6 | 0.3 | 0.9 | 12.3 | 11.6 | 13.6 | 11.1 | 2.5 |
| QCAZ-15930 | Santo de los Tsáchilas | *E. bicolor-marmoratus* complex | ND | Fully metamorphosed |  |  |  |  |  |  |  |  |  |  |  |
| ZSFQ-02819 | Santo de los Tsáchilas | *E. bicolor-marmoratus* complex | ND | Adult |  |  |  |  |  |  |  |  |  |  |  |
| QCAZ-08723 | Imbabura | *E. bicolor-marmoratus* complex | Male | Adult | 1.4 | 1.8 | 1.1 | 10.2 | 0.3 | 1.1 | 13.9 | 12.2 | 14.2 | 11.8 | 2.5 |
| QCAZ-03245 | Pichincha | *E. bicolor-marmoratus* complex | Female | Adult | 1.8 | 2.2 | 1.4 | 12.9 | 0.3 | 1.2 | 15.0 | 13.9 | 16.5 | 13.5 | 3.4 |
| DHMECN-09311 | Bolivar | *E. bicolor-marmoratus* complex | Female | Adult | 2.2 | 2.5 | 1.6 | 14.9 | 0.4 | 1.4 | 19.1 | 17.0 | 19.9 | 15.6 | 5.6 |
| DHMECN-01523 | El Oro | *E. bicolor-marmoratus* complex | Larvae | Stage 37 |  |  |  |  |  | 2.5 |  |  |  |  |  |
| DHMECN-10828 | El Oro | *E. bicolor-marmoratus* complex | Male | Adult | 1.4 | 2.4 | 1.1 | 14.3 | 0.3 | 1.2 | 19.1 | 15.1 | 17.3 | 14.7 | 2.4 |
| MEPN-05198 | El Oro | *E. bicolor-marmoratus* complex | Female | Fully metamorphosed | 1.2 | 1.7 | 0.7 | 9.5 | 0.3 | 0.5 | 11.3 | 9.9 | 11.6 | 9.6 | 2.3 |
| QCAZ-76506 | Zamora Chinchipe | *E. petersi* | Male | Adult | 1.6 | 1.5 | 0.7 | 10.8 | 0.3 | 1.1 | 11.9 | 12.3 | 14.9 | 12.0 | 3.4 |
| QCAZ-79921 | Zamora Chinchipe | *E. jameskenti* | Female | Adult | 1.5 | 2.0 | 1.4 | 11.8 | 0.2 | 0.9 | 12.3 | 12.5 | 13.3 | 11.6 | 3.8 |
| MUTPL-03997 | Morona Santiago | *E. jameskenti* | Larvae | Stage 38 |  | 1.8 | 0.7 | 5.6 | 0.3 | 0.5 | 8.8 | 6.3 | 8.4 | 6.1 | 1.9 |
| ZSFQ-04136 | Morona Santiago | *E. jameskenti* | Larvae | Stage 39 |  | 2.3 | 1.1 | 7.0 | 0.3 | 0.4 | 9.4 | 8.2 | 9.8 | 7.3 | 1.6 |
| ZSFQ-04137 | Morona Santiago | *E. jameskenti* | Larvae | Stage 39 |  | 2.2 | 0.9 | 6.7 | 0.3 | 0.4 | 9.2 | 8.1 | 10.9 | 9.3 | 2.5 |
| MZUA-02161 | Morona Santiago | *E. jameskenti* | Larvae | Stage 37 |  | 2.4 | 1.1 | 7.7 | 0.3 | 0.5 | 10.6 | 9.2 | 10.7 | 8.6 | 2.5 |
| DHMECN-04600 | Zamora Chinchipe | *E. jameskenti* | Male | Fully metamorphosed | 0.7 | 1.4 | 0.6 | 7.3 | 0.1 | 0.6 | 9.0 | 7.5 | 9.1 | 7.5 | 1.3 |
| MUTPL-03998 | Morona Santiago | *E. jameskenti* | Male | Fully metamorphosed | 1.3 | 1.7 | 0.7 | 8.3 | 0.2 | 0.7 | 10.0 | 8.9 | 11.0 | 8.9 | 1.8 |
| ZSFQ-04138 | Morona Santiago | *E. jameskenti* | Male | Fully metamorphosed | 1.3 | 1.6 | 0.8 | 7.8 | 0.3 | 0.7 | 10.2 | 8.5 | 11.4 | 8.0 | 2.8 |
| MZUA-02854 | Morona Santiago | *E. jameskenti* | Female | Fully metamorphosed | 1.0 | 1.6 | 0.6 | 9.1 | 0.3 | 0.8 | 10.3 | 9.8 | 13.1 | 9.9 | 2.7 |
| **Museum number** | **Locality** | **Species** | **Sex** | **Development st.** | **TAW** | **VA** | **ADD** | **C1** | **C2** | **CM** | **TA1** | **TA2** | **PVA** |  |  |
| QCAZ-42406 | Napo | *A. amazops* | Larvae | Stage 37 |  |  | 1.5 |  |  |  |  |  |  |  |  |
| ZSFQ-00814 | Napo | *A. amazops* | Larvae | Stage 37 | 4.1 | 5 | 1.8 | 1.4 | 4.2 | 26 | 271 | 265 | 13 |  |  |
| ZSFQ-00815 | Napo | *A. amazops* | Larvae | Stage 37 | 4.3 | 5 | 1.6 | 1.7 | 3.5 | 29 | 278 | 277 | 11 |  |  |
| DHMECN-19951 | Tungurahua | *A. amazops* | Larvae | Stage 37 | 3.2 |  | 2.8 | 1.5 | 3.2 | 22 |  |  |  |  |  |
| QCAZ-56870 | Napo | *A. amazops* | Male | Fully metamorphosed | 5.0 | 4 | 2.9 | 1.9 | 4.2 | 31 | 272 | 270 | 14 |  |  |
| QCAZ-70228 | Pastaza | *A. amazops* | Male | Fully metamorphosed | 4.3 | 4 | 2.0 | 1.5 | 4.9 | 28 | 275 | 264 | 12 |  |  |
| QCAZ-42407 | Napo | *A. amazops* | Male | Adult | 5.0 | 4 | 2.1 | 1.8 | 6.2 | 31 | 287 | 280 | 12 |  |  |
| MEPN-07801 | Napo | *A. amazops* | Male | Fully metamorphosed | 4.2 | 5 | 2.4 | 1.8 | 5.8 | 31 | 319 | 310 | 12 |  |  |
| DHMECN-15867 | Pastaza | *A. amazops* | Male | Fully metamorphosed | 4.5 | 4 | 1.4 | 1.7 | 5.0 | 23 | 276 | 275 | 10 |  |  |
| QCAZ-42405 | Napo | *A. amazops* | Male | Adult | 6.7 | 3 | 3.5 | 2.1 | 6.6 | 39 | 261 | 249 | 10 |  |  |
| DHMECN-14152 | Pastaza | *A. amazops* | Female | Fully metamorphosed | 5.5 | 4 | 2.0 | 1.7 | 3.6 | 28 | 298 | 292 | 11 |  |  |
| QCAZ-03210 | Napo | *A. amazops* | Female | Fully metamorphosed | 5.0 | 4 | 1.8 | 1.7 | 6.1 | 29 | 298 | 292 | 11 |  |  |
| QCAZ-74837 | Napo | *A. amazops* | Female | Adult | 7.1 | 5 | 3.7 | 2.5 | 7.5 | 44 | 350 | 347 | 13 |  |  |
| QCAZ-01965 | Cotopaxi | *E. bicolor-marmoratus* complex | Larvae | Stage 37 |  |  |  |  |  |  |  |  |  |  |  |
| QCAZ-01963 | Cotopaxi | *E. bicolor-marmoratus* complex | Larvae | Stage 37 |  |  |  |  |  |  |  |  |  |  |  |
| QCAZ-01977 | Cotopaxi | *E. bicolor-marmoratus* complex | Larvae | Stage 39 | 4.8 | 7 | 2.0 | 1.7 | 3.9 | 29 | 285 | 275 | 13 |  |  |
| QCAZ-01964 | Cotopaxi | *E. bicolor-marmoratus* complex | Larvae | Stage 37 | 4.7 | 5 | 1.4 | 1.6 | 4.8 | 24 | 276 | 259 | 12 |  |  |
| QCAZ-20138 | Santo de los Tsáchilas | *E. bicolor-marmoratus* complex | Male | Fully metamorphosed | 6.6 | 6 | 2.5 | 1.6 | 4.2 | 31 | 288 | 277 | 19 |  |  |
| QCAZ-02690 | Cotopaxi | *E. bicolor-marmoratus* complex | Male | Adult | 7.3 | 5 | 3.4 | 1.5 | 6.0 | 36 | 304 | 303 | 19 |  |  |
| QCAZ-01969 | Cotopaxi | *E. bicolor-marmoratus* complex | Male | Adult | 10.1 | 6 | 4.3 | 1.8 | 5.5 | 47 | 278 | 273 | 15 |  |  |
| QCAZ-01968 | Cotopaxi | *E. bicolor-marmoratus* complex | Male | Adult | 10.9 | 6 | 4.4 | 1.8 | 5.0 | 53 | 274 | 272 | 19 |  |  |
| QCAZ-01979 | Cotopaxi | *E. bicolor-marmoratus* complex | Male | Adult | 10.1 | 6 | 3.9 | 3.0 | 6.1 | 50 | 288 | 264 | 18 |  |  |
| QCAZ-01978 | Cotopaxi | *E. bicolor-marmoratus* complex | Male | Adult | 10.1 | 6 | 3.5 | 2.5 | 4.9 | 50 | 286 | 272 | 17 |  |  |
| ZSFQ-02818 | Santo de los Tsáchilas | *E. bicolor-marmoratus* complex | Male | Adult | 8.6 | 5 | 3.3 | 2.2 | 5.9 | 50 | 286 | 254 | 16 |  |  |
| QCAZ-01961 | Santo de los Tsáchilas | *E. bicolor-marmoratus* complex | Male | Adult | 8.8 | 6 | 2.7 | 3.1 | 6.5 | 49 | 279 | 266 | 16 |  |  |
| QCAZ-01970 | Cotopaxi | *E. bicolor-marmoratus* complex | Female | Adult | 10.5 | 5 | 3.6 | 2.0 | 5.5 | 51 | 254 | 239 | 17 |  |  |
| MZUTI-05543 | Cotopaxi | *E. bicolor-marmoratus* complex | Female | Fully metamorphosed | 7.2 | 4 | 2.3 | 2.0 | 4.4 | 37 | 311 | 305 | 18 |  |  |
| QCAZ-01966 | Santo de los Tsáchilas | *E. bicolor-marmoratus* complex | Female | Adult | 7.9 | 6 | 3.5 | 2.6 | 6.8 | 48 | 316 | 288 | 16 |  |  |
| QCAZ-01980 | Cotopaxi | *E. bicolor-marmoratus* complex | Female | Adult | 10.3 | 5 | 4.3 | 3.1 | 6.0 | 51 | 262 | 259 | 18 |  |  |
| ZSFQ-2817 | Santo de los Tsáchilas | *E. bicolor-marmoratus* complex | Female | Adult | 12.5 | 6 | 3.7 | 3.5 | 6.9 | 51 | 288 | 260 | 14 |  |  |
| DHMECN-19465 | Carchi | *E. bicolor-marmoratus* complex | Male | Adult | 10.6 | 6 | 4.6 | 2.9 | 5.7 | 51 | 293 | 289 | 17 |  |  |
| QCAZ-11861 | Santo de los Tsáchilas | *E. bicolor-marmoratus* complex | Female | Adult | 9.0 | 5 | 3.5 | 2.7 | 6.0 | 39 | 305 | 293 | 21 |  |  |
| QCAZ-15930 | Santo de los Tsáchilas | *E. bicolor-marmoratus* complex | ND | Fully metamorphosed |  |  |  |  |  |  |  |  |  |  |  |
| ZSFQ-02819 | Santo de los Tsáchilas | *E. bicolor-marmoratus* complex | ND | Adult |  |  |  |  |  |  |  |  |  |  |  |
| QCAZ-08723 | Imbabura | *E. bicolor-marmoratus* complex | Male | Adult | 8.9 |  |  | 2.4 | 7.3 | 40 | 323 | 311 | 21 |  |  |
| QCAZ-03245 | Pichincha | *E. bicolor-marmoratus* complex | Female | Adult | 8.3 | 8 | 4.3 | 2.2 | 6.8 | 53 | 331 | 312 | 20 |  |  |
| DHMECN-09311 | Bolivar | *E. bicolor-marmoratus* complex | Female | Adult | 12.1 | 6 | 5.2 | 4.0 | 8.4 | 68 | 335 | 326 | 21 |  |  |
| DHMECN-01523 | El Oro | *E. bicolor-marmoratus* complex | Larvae | Stage 37 |  |  |  |  |  |  |  |  |  |  |  |
| DHMECN-10828 | El Oro | *E. bicolor-marmoratus* complex | Male | Adult | 7.9 | 7 | 3.7 | 2.2 | 6.3 | 47 | 324 | 309 | 19 |  |  |
| MEPN-05198 | El Oro | *E. bicolor-marmoratus* complex | Female | Fully metamorphosed | 6.3 | 7 | 2.9 | 1.9 | 5.4 | 33 | 288 | 285 | 17 |  |  |
| QCAZ-76506 | Zamora Chinchipe | *E. petersi* | Male | Adult | 9.6 | 5 | 4.2 | 2.4 | 7.6 | 45 | 282 | 270 | 16 |  |  |
| QCAZ-79921 | Zamora Chinchipe | *E. jameskenti* | Female | Adult | 10.1 | 7 | 4.2 | 1.9 | 6.7 | 65 | 291 | 276 | 16 |  |  |
| MUTPL-03997 | Morona Santiago | *E. jameskenti* | Larvae | Stage 38 | 4.7 | 6 | 2.5 | 2.0 | 4.2 | 28 |  |  | 13 |  |  |
| ZSFQ-04136 | Morona Santiago | *E. jameskenti* | Larvae | Stage 39 | 5.3 | 6 | 1.7 | 1.7 | 2.6 | 29 | 279 | 273 | 12 |  |  |
| ZSFQ-04137 | Morona Santiago | *E. jameskenti* | Larvae | Stage 39 | 5.0 | 7 | 1.9 | 1.8 | 2.7 | 27 | 271 | 265 | 15 |  |  |
| MZUA-02161 | Morona Santiago | *E. jameskenti* | Larvae | Stage 37 | 6.5 | 7 | 2.9 | 2.1 | 2.8 | 33 | 298 | 290 | 19 |  |  |
| DHMECN-04600 | Zamora Chinchipe | *E. jameskenti* | Male | Fully metamorphosed | 4.9 | 6 | 1.6 | 1.4 | 4.0 | 25 | 264 | 262 | 17 |  |  |
| MUTPL-03998 | Morona Santiago | *E. jameskenti* | Male | Fully metamorphosed | 6.2 | 5 | 2.6 | 1.7 | 4.1 | 30 | 258 | 257 | 17 |  |  |
| ZSFQ-04138 | Morona Santiago | *E. jameskenti* | Male | Fully metamorphosed | 6.4 | 7 | 2.5 | 2.2 | 5.8 | 32 | 291 | 290 | 19 |  |  |
| MZUA-02854 | Morona Santiago | *E. jameskenti* | Female | Fully metamorphosed | 6.4 | 5 |  | 2.0 | 4.8 | 33 | 269 | 264 | 20 |  |  |
